# Old and New Stories: Revelations from Functional Analysis of the Bovine Mammary Transcriptome during the Lactation Cycle

**DOI:** 10.1371/journal.pone.0033268

**Published:** 2012-03-12

**Authors:** Massimo Bionaz, Kathiravan Periasamy, Sandra L. Rodriguez-Zas, Robin E. Everts, Harris A. Lewin, Walter L. Hurley, Juan J. Loor

**Affiliations:** 1 Department of Animal Sciences, University of Illinois, Urbana, Illinois, United States of America; 2 Institute for Genomic Biology, University of Illinois, Urbana, Illinois, United States of America; 3 Animal Production and Health Section, Seibersdorf Laboratories Joint FAO/IAEA Division of Nuclear Techniques in Food and Agriculture International Atomic Energy Agency, Vienna, Austria; 4 Division of Nutritional Sciences, University of Illinois, Urbana, Illinois, United States of America; University of South Florida College of Medicine, United States of America

## Abstract

The cow mammary transcriptome was explored at −30, −15, 1, 15, 30, 60, 120, 240, and 300 d relative to parturition. A total of 6,382 differentially expressed genes (DEG) at a false discovery rate ≤0.001 were found throughout lactation. The greatest number of DEG (>3,500 DEG) was observed at 60 and 120 d vs. −30 d with the largest change between consecutive time points observed at −15 vs. 1 d and 120 vs. 240 d. Functional analysis of microarray data was performed using the Dynamic Impact Approach (DIA). The DIA analysis of KEGG pathways uncovered as the most impacted and induced ‘Galactose metabolism’, ‘Glycosylphosphatidylinositol (GPI)-anchor biosynthesis’, and ‘PPAR signaling’; whereas, ‘Antigen processing and presentation’ was among the most inhibited. The integrated interpretation of the results suggested an overall increase in metabolism during lactation, particularly synthesis of carbohydrates and lipid. A marked degree of utilization of amino acids as energy source, an increase of protein export, and a decrease of the protein synthesis machinery as well cell cycle also were suggested by the DIA analysis. The DIA analysis of Gene Ontology and other databases uncovered an induction of Golgi apparatus and angiogenesis, and the inhibition of both immune cell activity/migration and chromosome modifications during lactation. All of the highly-impacted and activated functions during lactation were evidently activated at the onset of lactation and inhibited when milk production declined. The overall analysis indicated that the bovine mammary gland relies heavily on a coordinated transcriptional regulation to begin and end lactation. The functional analysis using DIA underscored the importance of genes associated with lactose synthesis, lipid metabolism, protein synthesis, Golgi, transport, cell cycle/death, epigenetic regulation, angiogenesis, and immune function during lactation.

## Introduction

Worldwide human consumption of bovine milk and bovine milk-derived products has increased in the last decades, especially in countries where this food was not part of the traditional diet [1]. The bovine mammary gland is an extraordinary organ able to produce >30,000 kg of milk in a complete lactation cycle, e.g., the top producing US Holstein in 1997 synthesized ca. 100 kg milk/d [2] representing >45-fold of the cow's body weight (ca. 12 kg/d of total milk solid which is equivalent to >5-fold the cow's body weight per year) while the Star of the Breed 2008 produced 42,270 kg of milk in 365 day of lactation (>60-fold the body weight) [2]. The anabolic capacity of this organ in modern dairy cows is so remarkable that some have even suggested that the animal might be considered an appendage of the mammary gland [3].

The past several decades have seen major advances in understanding the physiology of lactating mammary gland driven partly by the desire to improve the efficiency of production but also the quality of the milk (e.g. [3]). Despite those efforts, the cellular adaptations required for the synthesis and secretion of milk by this extraordinary organ remain largely unknown. The lack of suitable techniques has certainly limited a holistic and integral view of the physio-cellular adaptations in bovine mammary tissue. The development of high-throughput tools such as microarray analysis has provided the unique possibility to uncover the molecular networks in mammary tissue during the course of pregnancy, milk synthesis, and involution. Microarray analyses have been conducted in mouse to uncover mammary gland adaptations during the lactation cycle [4], [5]; however, until recently the interpretation of this large amount of data was extremely challenging. The advent of bioinformatics tools has allowed summarizing large microarray data sets, revealing an integral view of cellular phenomena. Such an approach has been performed on mouse mammary tissue microarray data [6].

The objective of our study was to undertake an integrated functional study of the mammary tissue transcriptome in Holstein dairy cows from late pregnancy through the end of the subsequent lactation by means of the validated Dynamic Impact Approach (DIA) (companion paper).

## Results and Discussion

### Milk yield and composition during lactation

The average curve of lactation for the cows used in the present experiment is reported in Figure S1. Briefly, as expected for high-producing Holstein dairy cows the peak of lactation was reached at ca. 40 days in milk with more than 39 kg of milk per day. This rate of production was maintained, with some variation, through 170 days in milk (Figure S1). The extended peak of lactation was due to the use of bovine somatotropin. Milk composition and fatty acid composition of milk fat were published and discussed previously [7], [8].

### Overall pattern in transcriptome expression

**Figure 1:**
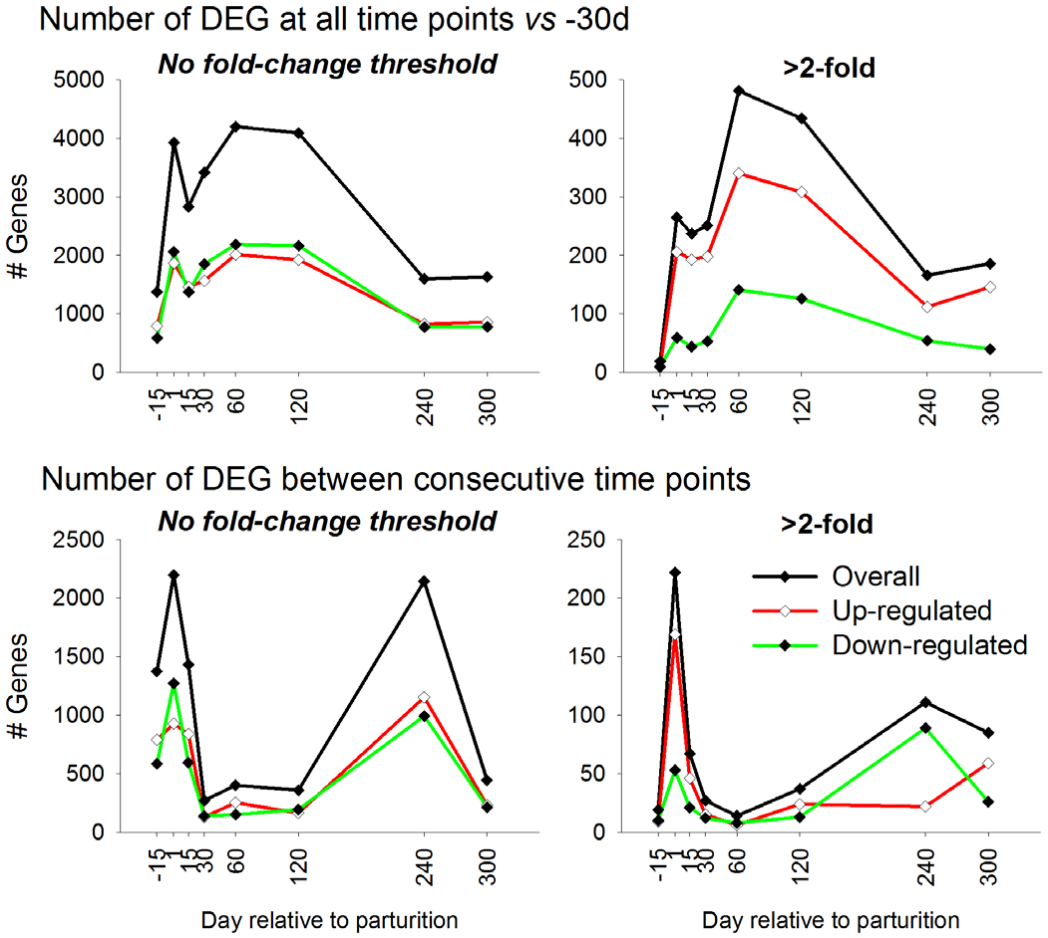
Number of differentially expressed genes with FDR≤0.001 and post-hoc P<0.001 at each time relative to −30 d (pregnancy) (upper left panel), and relative to the previous time (bottom left panel). A 2-fold (100%)-change threshold was applied for both the panel encompassing each time relative to −30 d (pregnancy) (upper right panel) and the comparison of each time relative to the previous time point (bottom right panel). Applying such a threshold highlighted the increase in the proportion of up-regulated genes within DEG that were markedly affected. Additional images with several thresholds for each type of comparison are available in Figure S2).

The correction of P-values using a false discovery rate (FDR)≤0.001 and a post-hoc P-value<0.001 between comparisons (in at least one comparison considered) uncovered 6,382 differentially expressed genes (DEG) throughout lactation (4,956 unique annotated genes; complete dataset is available in the companion paper). The overall number of DEG at each time vs. −30 d with a post-hoc correction of P<0.001 (Figure 1 upper panels) revealed a biphasic pattern of adaptation by stage of lactation, a peak in the number of DEG at the onset of lactation (1 d) and again at 60-to-120 d relative to −30 d. Considering all DEG without fold change cut-off (Figure 1, top left panel), the number of down-regulated (**⇓**) DEG was slightly greater than up-regulated (**⇑**) DEG, especially between 60 and 120 d. The application of a ≥2-fold threshold (i.e., 100% change; Figure 1, top right panel) indicated that most of the genes with greatest changes in expression were ⇑ genes while the magnitude of the effect on ⇓ genes was modest. In addition, at 60 and 120 vs. −30 d there was the greatest number of genes with ≥2-fold change (>300 genes or >13% of DEG), which coincided with the plateau of daily milk production (Figure S1). Additional results by using different cut-offs are reported in Figure S2. From those results it appeared evident that almost all the DEG had a ≥1.3-fold change (or 30% increase/decrease in expression).

The analysis of DEG (post-hoc P<0.001) at a subsequent time relative to the previous sampling (i.e., consecutive time point; Figure 1, bottom left panel) highlighted the switch of a large number of genes (>2,000) at the onset of lactation (i.e., between −15 and 1 d) and again between 120 and 240 d, with relatively few changes in DEG in the remaining comparisons. In the absence of a fold-change cut-off, it was evident that there was a greater number of ⇓ DEG between −15 and 1 d and ⇑ DEG between 120 and 240 d (Figure 1, bottom left panel). When a 2-fold cut-off was applied (Figure 1, bottom right panel), it was clear that the majority of genes with a large fold change was ⇑ between −15 and 1 d, coinciding with the period around parturition, and ⇓ between 120 and 240 d, coinciding with late lactation. The latter corresponded with the beginning of the decline in milk production (Figure S1).

Overall, the results indicated that there was a slightly greater number of ⇓ than ⇑ DEG in bovine mammary tissue during lactation relative to pregnancy; however, few of the ⇓ DEG had a fold change ≥2. Thus, among the genes with fold change ≥2 the majority were ⇑ (ca. 70–80% of total DEG ≥2-fold change). To some extent, the temporal increase from pregnancy through 120 d in the number of DEG followed a similar pattern as the curve of lactation (Figure S1).

With the limitations of species comparisons, these data differed from mouse mammary tissue [6], where the number of DEG increased gradually from early pregnancy through 2 days prior to parturition and at parturition there was only a limited increase in number of DEG. Furthermore, changes observed in our study were more abrupt at the onset of lactation compared with the mouse [6]; however, the first biopsy sample was harvested at 2 weeks prior to expected parturition, and it could be possible that during those 2 weeks there were progressive changes in overall transcription. This point is supported by a previous microarray experiment with bovine mammary tissue around parturition [9] in which biopsy samples were collected closer to parturition and with greater variation in time between samplings (i.e., 5±5 d prior parturition). In that experiment there was no significant increase in expression of casein or alpha-lactalbumin genes from 5 days prior parturition to 10 days in milk [9], a range which was smaller than any of our sampling times around parturition (i.e. 1 vs. −15 d or 15 vs. 1 d). Nevertheless, our results indicated that bovine mammary tissue relies heavily on transcriptional regulation of genes to induce copious milk synthesis and secretion.

An additional consideration is that the rise in milk production in early lactation in the mouse is driven by the energy needs of the pups (i.e. suckling stimulus), with involution commencing soon after pups are removed from the dam. Pups removal induces a large adaptation in mammary gene expression [6]. In the bovine, milk production reaches a peak during the first two months of lactation which is maintained at that level for ca. 3–4 months, and followed by a decline. When cows are treated with exogenous bovine somatotropin the period of peak milk production is extended, as was the case in our study (Figure S1). Our study revealed a dramatic alteration in gene expression at the point when milk production began to decline (i.e., between 120 and 240 d; Figure 1 and Figure S1). This effect supports an active role of the mammary transcriptome in the regulation of milk yield.

### Impact of DEG on KEGG pathways

#### Overall view of KEGG pathways

**Figure 2:**
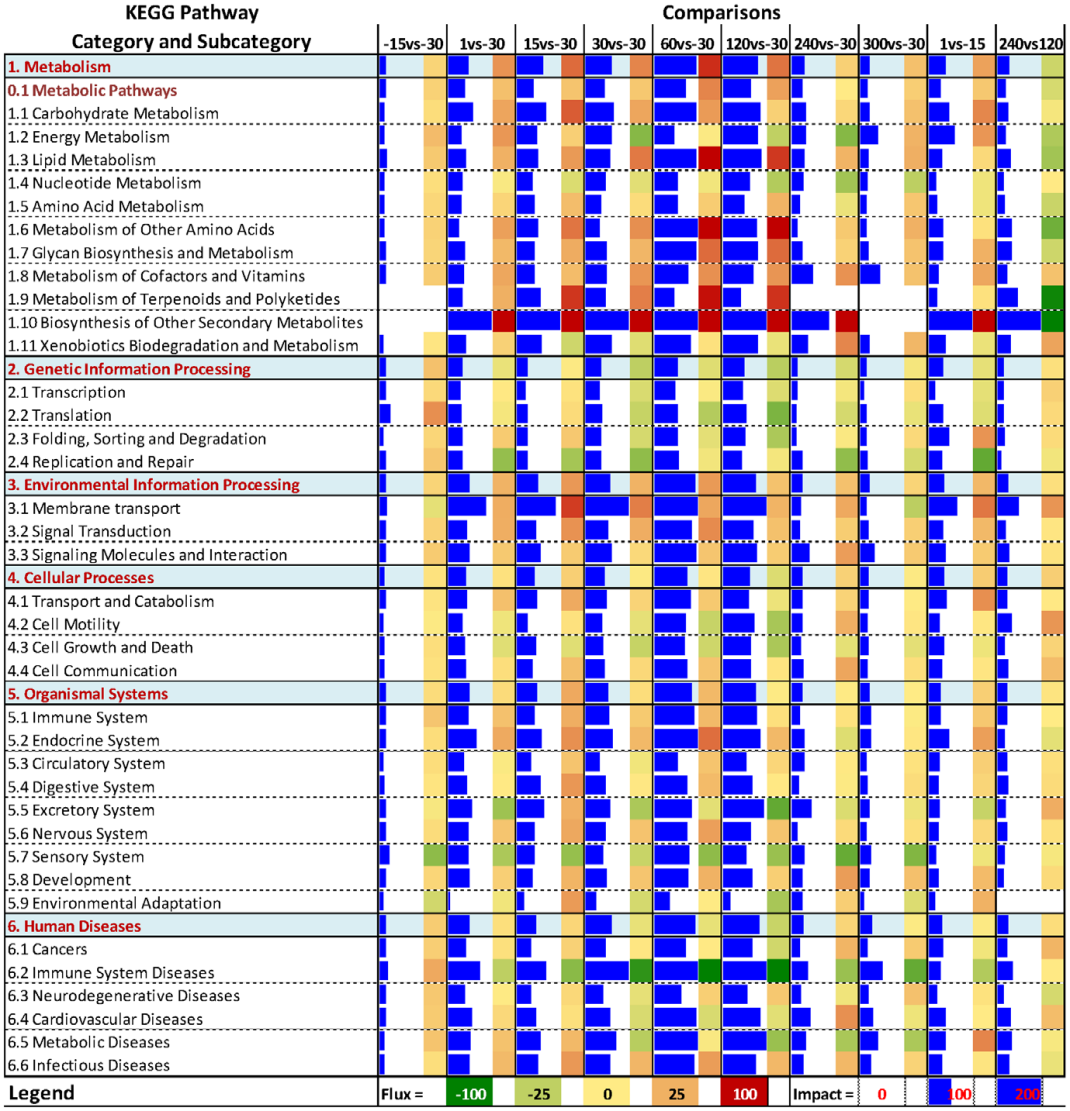
Overall view of the DIA results of the KEGG pathways with main categories and subcategories as provided by KEGG (http://www.genome.jp/kegg/pathway.html). Reported are all times compared with −30 d, and the two consecutive times (1 vs. −15 d and 240 vs. 120 d) with the largest number of differentially expressed genes and overall impact on pathways. Blue horizontal bars denote the impact and the square on their right denote the direction of the impact (red = activation; green = inhibition). See companion paper for interpretation of the DIA output.

The overall view of the impact of DEG during lactation on KEGG pathway categories is shown in Figure 2. The pathways most impacted during lactation were related to the category ‘Metabolism’ (in particular the subcategory ‘Carbohydrate Metabolism’, ‘Lipid Metabolism’, ‘Amino Acid Metabolism’, ‘Glycan Metabolism’, and secondary metabolites, such as ‘Metabolism of Other Amino Acids’ and ‘Biosynthesis of Other Secondary Metabolites’, all of them strongly induced), ‘Environmental Information Processing’ (evidently induced), and ‘Organismal System’ (with ‘Immune System” and ‘Endocrine System’ induced and ‘Excretory System’ and ‘Sensory System’ inhibited during lactation). Interestingly, the ‘Metabolism’ category was induced overall at the onset of lactation (i.e., 1 vs. −15 d) and then inhibited at 240 vs. 120 d, when milk yield decreased (Figure 2 and Figure S1). The subcategories of pathways related to ‘Human Diseases’ probably have little to do with bovine mammary and will not be discussed in detail; however, the results indicated that these pathways were strongly impacted overall during lactation and inhibited as lactation progressed. The most inhibited subcategory was the ‘Immune System Diseases’ (Figure 2).

In summary, the examination of KEGG pathway categories and subcategories indicated that during lactation the mammary gland increased overall metabolism, e.g. large increase in carbohydrate, lipid, and metabolism of other secondary molecules (e.g., glycans). Those events were coupled with a large increase in membrane transport and signaling and a strong participation of the immune and endocrine system. It was noted that pathways in the ‘Genetic information processing’ subcategory were slightly inhibited overall as lactation progressed. That was mostly due to an evident inhibition of ‘Translation’ and ‘Replication and Repair’ subcategories (Figure 2).

#### Most impacted KEGG pathways

The detail of each KEGG pathway is reported in Additional file S1 (sheet ‘KEGG pathway’). The top impacted pathways as calculated and sorted by the mean impact from 15 to 120 vs. −30 d (with exclusion of ‘Human Diseases’ category) were ‘Caffeine metabolism’, ‘Galactose metabolism’ (i.e., lactose synthesis), ‘Glycosylphosphatidylinositol(GPI)-anchor biosynthesis’, and ‘PPAR signaling’. Some of those pathways were discussed in the companion paper. Among the top impacted pathways not discussed in the companion paper, the ‘Glycosylphosphatidylinositol (GPI)-anchor biosynthesis’, the “Sterol biosynthesis”, and the “ABC transporters” are novel and of biological interest.

The ‘Glycosylphosphatidylinositol (GPI)-anchor biosynthesis’ was the most impacted among the glycan biosynthesis-related pathways (Figure 2; details in Additional file S1). The pathway is involved in synthesis of GPI which anchors proteins in the membrane of the ER for transport to the Golgi and apical membranes [10]. The importance of the GPI-anchored proteins has not been evaluated in bovine mammary gland and although the concentration of GPI anchored proteins is high in human and pig milk, it is apparently undetectable in bovine milk [11]. GPI-anchors are pivotal for cell survival and affect many functions such as protein sorting in ER-Golgi trafficking, targeting of GPI-anchored proteins in polarized epithelial cells for apical export, cell-to-cell adhesion, signal transduction associated with cholesterol and sphingolipids in membrane rafts, and sorting in endocytic pathways [12]. Except for transport of folic acid through the internalization of its receptor, which is a known GPI-anchored protein, to date there are no known roles of GPI-anchored proteins in milk synthesis [12]. Thus, this finding is novel and more research on GPI-anchored proteins seems warranted.

The DIA analysis of the ‘Steroid biosynthesis’ indicated an overall induction of this pathway (Additional file S1). In addition, the visualization of the pathway (Additional file S2) indicated an overall increase of cholesterol synthesis. Even though it has been demonstrated that the major part of cholesterol present in bovine milk originates from hepatic synthesis [13], and that the synthesis of cholesterol in mammary has been poorly studied in bovine, it has been shown that *de novo* cholesterol synthesis increases after parturition in rat mammary tissue [14]. Linked with cholesterol synthesis also is the ‘ABC transporters’ which was induced overall during lactation underscoring the importance of those transporters in lactating mammary gland (Additional file S1). Several ABC transporters seem to be involved in cholesterol transport and some are down-regulated during lactation in bovine mammary tissue [8], [15]. Others with still unknown function are highly abundant in bovine mammary tissue during lactation (e.g., *ABCG2*, [8]; see also companion paper).

#### Overall interpretation of KEGG pathways: Metabolism

The pathways in the cell are highly interconnected and, for metabolic pathways, quite often the product of one pathway becomes the substrate for another. Here we attempt to provide an interpretative summary of the metabolism in mammary tissue during lactation based upon the results from the DIA analysis (see ‘KEGG Pathways’ sheet in Additional file S1).

The overall metabolism increased during lactation, as shown by the ‘Metabolism’ category (Figure 2). The overall visualization of the data (Additional file S1 and S2) suggested a channeling of glucose towards increasing the synthesis of lactose and decreasing use for energy (i.e., decrease of glycolysis and pyruvate metabolism). The DIA suggested that the ‘Lipid Metabolism’ subcategory was characterized by a large increase in synthesis of triacylglycerol (TAG) (through activation of ‘Biosynthesis of unsaturated fatty acids’, ‘Ether lipid metabolism’, ‘Glycerolipid metabolism’, and ‘Glycerophospholipid metabolism’ pathways, Additional file S1), synthesis of phospholipids (‘Glycerophospholipid metabolism’), cholesterol (‘Steroid biosynthesis’), leukotrienes (through ‘Arachidonic acid metabolism’, see Additional file S2), and sphingolipids (‘Sphingolipid metabolism’) and a reduction of ‘Lipid metabolism’ (i.e., lipid catabolism), ‘Steroid hormone biosynthesis’, and ‘Fatty acid elongation in mitochondria’ (Additional file S1). The suggested reduction of fatty acid metabolism together with an increase in synthesis of TAG indicated a preferential channeling of fatty acids taken up by the mammary tissue [16] towards synthesis of milk fat, including the components of cellular membranes.

Results also indicated an increase in catabolism of several amino acids (AA) (i.e., induction through lactation of ‘Arginine and proline metabolism’, ‘Histidine metabolism’, ‘Lysine degradation’, ‘Tryptophan metabolism’, and ‘Valine, leucine and isoleucine degradation’, Additional file S1). Many of the products of those pathways could be precursors of the TCA cycle pathway (Additional file S2) that the DIA suggested was strongly activated during lactation (Additional file S1). Considering that our data indicated a reduction of flux in the relative proportion of glucose and fatty acids for energy utilization and an increase in the proportion of AA import (see below and [7]) and degradation to potentially provide precursors for the TCA cycle, it appears that the AA are an important pool of energy in mammary tissue during lactation, particularly the class II essential AA [7] (i.e., Arg, Ile, Leu, Lys, and Val). These findings are supported by experiments conducted more than 30 years ago [17], [18]. The ‘Cysteine and methionine metabolism’ pathway was inhibited during lactation and induced at 240 *vs.* 120 d (Additional file S1, ‘KEGG Pathways’ sheet). This result suggests that the mammary gland spared Met for protein synthesis, which is supported by Met being one of the AA with lowest concentrations in blood but one with the highest extraction ratios by the mammary gland [19]. The synthesis of Lys, Phe, Tyr, and Thr was not impacted in any comparison.

The KEGG category ‘Metabolism of Other Amino Acids’ appeared to be relatively impacted and generally induced particularly during mid-lactation. Among those were ‘Beta-Alanine metabolism’, ‘Cyanoamino acid metabolism’, ‘Selenoamino acid metabolism’, ‘Glutathione metabolism’ and ‘Taurine and hypotaurine metabolism’ (Additional file S1 and S2). The large impact and induction of ‘Glutathione metabolism’ during lactation appears to confirm previous data [17], [20], and support a pivotal role of this process in AA availability to the mammary gland. The importance of the other pathways in milk synthesis needs to be addressed with additional investigations.

The DIA uncovered a large increase of TCA cycle activity and generation of energy in general, which is supported by activation of the ‘Oxidative phosphorylation’ and ‘Pentose and glucoronate interconversions’ pathways during lactation (Additional file S1). Changes in pathway related to AA suggested a likely increase in TCA intermediates, hence, TCA activity from AA degradation. Contrary to previous reports [21], [22], our data indicated that the ‘Pentose phosphate pathway’ was not highly impacted and not greatly activated during lactation (Additional file S1 and S2).

The importance of ‘Glycan Biosynthesis and Metabolism’ KEGG subcategory in mammary during lactation was evidenced in DIA by a large impact and induction of ‘GPI-anchor biosynthesis’ as discussed above, but also by induction of several other pathways involved in glycosaminoglycan biosynthesis, including ‘Chondroitin sulfate’, ‘Glycosphingolipid biosynthesis’ pathways, particularly ganglio- series, and ‘N-Glycan biosynthesis’ (see Additional file S1 for details). The importance of those pathways seemed to increase as lactation progressed until 120 vs. −30 d. The components synthesized by those pathways are essential for the structure of the extracellular matrix and the complex interactions which regulate cell-to-cell signaling, coordination of stromal-epithelial development, and the interaction with extracellular signaling molecules [23]. Although their quantity in bovine milk is low, the process of glycosphingolipid synthesis is of particular interest because of the current commercial focus in the production of these bioactive molecules due to their probiotic role in the human intestine [24]. In addition, gangliosides have an important role in membrane function such as modulating enzyme properties, cell signaling, cell adhesion, protein sorting, and formation of caveolae [25]. The globosides are present in bovine milk but at low concentrations [26], which our data appear to support (Additional file S1). A recent characterization of bovine milk oligosaccharides in colostrum and milk revealed a large decrease of their concentration during the transition from colostrum to mature milk as well as during the progression of lactation [27]. Our data, similar to recent transcriptomic data [28], suggested a potential overall increase in glycosphingolipids during lactation; however, the amount of those components decreases as lactation progresses [27].

Among the KEGG subcategory ‘Metabolism of Cofactors and Vitamins’ several pathways appeared to be impacted and largely induced during lactation, with ‘Caffeine metabolism’ being the most impacted among all KEGG pathways (Additional file S1). Most of those pathways were composed of only one gene that passed the selected thresholds (FDR and post-hoc P-value≤0.001 and ≥40% of the pathway covered by the microarray) rendering the interpretation of the data weak. The discussion of the ‘Caffeine metabolism’ was provided in the companion paper.

#### Overall interpretation of pathways: Genetic Information Processing

The KEGG category ‘Genetic Information Processing’ was not highly impacted overall during lactation except at mid-to-late lactation when the impact increased and an overall inhibition was apparent as lactation progressed (Figure 2). Among the pathways in this category the only ones induced during lactation were ‘RNA polymerase’, ‘Aminoacyl-tRNA biosynthesis’ (particularly during the first 60 d post-partum), ‘Protein export’, and ‘Protein processing in endoplasmic reticulum’ (Additional file S1 and S2).The KEGG pathways ‘Ribosome’, ‘Ribosome biogenesis in eukaryotes’, ‘RNA transport’, ‘RNA degradation’, ‘Sulfur relay system’ (particularly the tRNA thiolation for which a function is still unknown in eukaryotes [29]) and all the pathways involved in nucleotide replication and repair subcategory were clearly inhibited during lactation (Additional file S1). The pattern of ‘Ribosome’ had a strong induction and impact at −15 vs. −30 d (i.e., 2 weeks prior parturition), a minimal impact at the beginning of lactation and a consistent increase in impact and inhibition as lactation progressed and ended relative to −30 d (Additional file S1). This latter observation, while consistent with observations in mouse mammary tissue during lactation [6], does not seem to support the apparent increase in protein synthesis inferred by transcriptome analysis suggested by Finucane et al. [9]. The above observations together with the slightly greater number of down-regulated genes (Figure 1) and the significant increase in total amount of RNA during lactation [30], likely due to the large increase in expression of few genes (e.g. caseins and other milk proteins), appear to support the existence of “translational competition” within the mammary gland during lactation [7].

Overall, the data indicated that during lactation the mammary tissue increased processing and export of proteins from the ER and, despite the decrease of the protein synthesis machinery, it also increased AA charging to tRNA and the export of proteins. Those observations appear to be supported by the overall increase in milk protein synthesis and secretion by the mammary gland [31], [32]. As discussed below, the data also indicated a decrease in overall cell cycle (e.g., inhibition of ‘DNA replication’, see Additional file S1), which supports previous observations [9].

#### Overall interpretation of pathways: Environmental Information Processing

This category of KEGG pathways was highly impacted and activated overall during lactation (Figure 2), suggesting an general increase in intra- and extra-cellular signaling during lactation. Among the pathways in this category the most impacted and induced during lactation were ‘Hedgehog signaling’, ‘Jak-STAT signaling’, and ‘TGF-beta signaling’; whereas, ‘Cell adhesion molecules (CAMs)’, ‘mTOR signaling pathway’, and ‘Notch signaling pathway’ were inhibited (Additional file S1 and S2).

The Hedgehog and TGF-beta signaling pathways (together with ‘Wnt signaling’) appear to have a role in mammary stem cell self-renewal and, when deregulated, mammary cancer [33]. The Hedgehog signaling pathway has been previously reported to be induced during lactation in rodents and with an essential, albeit unknown, role in lactation [34]. Our data supported those previous findings. The importance of TGF-beta in mammary gland is still unclear but a consistent increase in its concentration in bovine milk has been demonstrated with a likely beneficial biological effect on human intestinal cells [35]. In addition, the TGF-beta pathway appears to have a negative role on mammary cell proliferation and a positive role on apoptosis [36]. The TGF-beta signaling through Smad is known to play a pivotal role in the cell cycle [37].

The Jak-STAT signaling is essential for the induction of milk protein expression in mammary tissue of non-ruminants, but in ruminants and particularly in the bovine, expression of milk proteins appears to require a basic activity of this pathway but is not directly modulated by the change in expression of Jak and/or Stat genes, rather, it seems to be modulated by down-stream effectors of the pathway (e.g., *ELF5*) [7].

The inhibition of Notch signaling pathway was a novel finding. This pathway is known to be involved in cell development and physical organization of cell into tissues, cell proliferation and apoptosis [38] and in mammary cell lines it has been observed that artificial activation of Notch signaling inhibit mammary branching and lactation [39].

The cell adhesion molecules are important in maintenance of structural architecture of the mammary epithelia [40]. Thus, the apparent inhibition of the ‘Cell adhesion molecules’ KEGG pathway is not clear; however, when the pathway was evaluated in larger details (Additional file S2) it became clear that the inhibition was mostly caused by adhesion molecules related to the immune system, but there was a clear induction of the tight junction molecules (see below).

The reduction of ‘mTOR signaling pathway’ was unexpected given that mTOR seems to play a role in milk protein synthesis. Previous data from our laboratory and from others ([7] and references therein) clearly indicated that mTOR plays an important role in milk protein synthesis; however, at the transcriptomics level the regulation was evident only for few of the mTOR pathway-related genes [7]. The present data appear to support our previous conclusion [7] that the process of translation is basically inhibited (via inhibition of mTOR signaling) and the inhibition is overridden by insulin signaling. In the present analysis insulin signaling was overall slightly inhibited but highly-impacted by lactation (Additional file S1). In the previous analysis [7] we measured few genes involved in insulin signaling and the data indicated enhanced sensitivity of the mammary gland to insulin via the increases in expression of the receptor and few down-stream signaling molecules. Most of those molecules were also found to be up-regulated with the microarray analysis and the overall inhibition was due, for the most part, to a decrease in expression of genes involved in non-mTOR related pathways (Additional file S2).

#### Overall interpretation of pathways: Cellular Processes

This category of pathways also was highly activated during lactation, particularly between 60 and 120 d compared to −30 d, with an overall induction of subcategory ‘Transport and Catabolism’ and ‘Cell Communication’ and an general inhibition of ‘Cell Growth and Death’ (Figure 2). The most impacted and activated pathways in this category during lactation were ‘Peroxisome’ and ‘Tight junctions’; ‘Phagosome’, ‘p53 signaling’, and ‘Gap junctions” appeared to be inhibited during the late lactation, while ‘Cell cycle’ was clearly inhibited during the whole lactation (Additional file S1).

Peroxisomes have been reported to be present in human mammary tissue and their activity is decreased in breast cancer [41]; however, we are unaware of published studies on peroxisomes in bovine mammary tissue. Our data indicated an overall induction of peroxisome activity and proliferation during lactation; particularly induced were peroxisome biogenesis, the prime metabolism (⇑ *XDH*), and the antioxidant activity (⇑ *SOD*), but an evident decrease of fatty acid oxidation was suggested as well (Additional file S2).

The importance of junctions in mammary tissue integrity has been understood for some time [42]. All the junctions appeared to have been induced overall during lactation (Additional file S1 and S2), but particularly the ‘Tight junctions’ and the ‘Adherens junctions’ were induced. Among all junctions, the tight junction has been known to be the most important during lactation, when it blocks the paracellular route allowing the highly-controlled secretion of milk components by the epithelial cells [43].

The inhibition of ‘Cell cycle’ coupled with the low impact of ‘Apoptosis’ (Additional file S1 and S2) indicated a reduction of proliferation but also a stasis from the point of view of number of cells in the mammary gland during lactation, which is in accordance with previous bovine mammary transcriptomics data in early lactation [9]. Measurements of bovine mammary parenchymal tissue apoptosis and proliferation at both the end of pregnancy and lactation have been performed [44], [45]. Data from those work indicated that apoptosis was higher at 48 days prior to parturition, decreased 2 weeks prior to parturition, increased 2 weeks after parturition where it reached a peak, and decreased thereafter with a numerical increase at mid-to-late lactation (90–120 d) [44], [45]. Those work also uncovered that proliferation of mammary cells decreases significantly from 2 weeks prior to parturition and remains low until late lactation, with a slight, albeit significant increase from 2 weeks postpartum to mid-lactation [45]. Our data appeared to be in accord with those previous reports (Additional file S1), indicating a stabilization of cell numbers at parturition and during most of lactation. The DIA results indicated that both apoptosis and proliferation resumed slightly between 120 and 240 d (Additional file S1), suggesting a decrease of cell number when milk yield declined, in accordance with previous data [46].

#### Overall interpretation of pathways: Organismal System

The ‘Organismal System’ category of KEGG pathways also was highly impacted (Figure 2). An apparent large impact and induced activity was observed for “Endocrine System’, ‘Immune System’, ‘Nervous System’, and ‘Circulatory System’ subcategories and an important impact and inhibition was observed for ‘Excretory System’ and ‘Sensory System’ subcategories (Figure 2). Among the pathways included in those subcategories the ‘Antigen processing and presentation’, the ‘Proximal tubule bicarbonate reclamation’, and the ‘PPAR signaling pathway’ were the most impacted. The former were clearly inhibited, while ‘PPAR signaling’ was clearly activated (Additional file S1).

In the ‘Immune System’ subcategory during lactation besides an activation and large impact of ‘Complement and coagulation cascades’ and ‘Hematopoietic cell lineage’ and inhibition of the ‘Antigen processing and presentation’, all the other immune-related pathways were modestly impacted and activated overall. This was most evident in mid-to-late lactation (i.e., after 60 d; see Additional file S1). For the ‘Antigen processing and presentation’ the major histocompatibility complex (MHC) class I was particularly inhibited during the whole lactation (Additional file S1 and S2). From the beginning of lactation to 120 d compared to −30 d, the overall analysis indicated a consistent increase in impact and induction of pathways related to the innate immune system (e.g., ‘Toll-like receptor signaling’, ‘NOD-like receptor signaling’, ‘RIG-I-like receptor signaling’, and ‘Cytosolic DNA-sensing’ pathways). Also pathways related to immune cells (e.g., ‘B cell receptor signaling’, ‘Leukocyte transendothelial migration’, ‘Natural killer cell mediated cytotoxicity’, and ‘T cell receptor signaling’ pathways) were induced and impacted to the same degree during lactation (Additional file S1). Overall, the data indicated a consistent increase of amount/activity of immune cells in mammary tissue until the decline of milk synthesis; however, there was a concomitant large decrease of MHC indicating the absence of hyper-activation of the immune system.

Among the pathways composing the ‘Endocrine System’, the ‘PPAR signaling’ and ‘GnRH signaling’ pathways were among the most impacted and induced during lactation. In contrast, ‘Adipocytokine signaling’ was moderately impacted and mostly inhibited during lactation and ‘Insulin signaling’ appeared slightly inhibited overall during lactation (Additional file S1).

The apparent induction of ‘GnRH signaling’ is novel. The gonadotropin-releasing hormone receptor (GnRHR) is typically expressed in the anterior pituitary to regulate, under stimulus of the GnRH, the production and release of the gonadotropins, luteinizing and follicle-stimulating hormones. The GnRHR, however, has also been found in extrapituitary tissues including mammary gland [47]. The function of GnRHR in extrapituitary tissues is not entirely clear but it appears to play a role in reducing cell proliferation in breast cancer cells [47]. Interestingly, our data indicated that the expression of genes coding for the beta polypeptide of both luteinizing and follicle-stimulating hormones (*LHB* and *FSHB*) was significantly ⇑ during lactation (see companion paper and Additional file S2). An increase in expression of genes coding for those hormones in mammary tissue during lactation also was reported in microarray data from mouse [4], [5]. This finding is novel and, to our knowledge, no extrapituitary protein expression of those genes has been reported previously in bovine mammary tissue. Many hormones have been detected in milk but not LH or FSH [48]. We have not verified such expression using qPCR or Western blot; thus, further investigation is required to verify these findings.

Due to specific functions that are not pertinent to the mammary, the pathways in others subcategories under ‘Organismal System’ are probably not applicable *in toto* to mammary gland (e.g., ‘Digestive System’, ‘Excretory System’); however, a similar function can be envisaged in mammary tissue for several DEG. In particular, a few observations can be made by using Figure 2 and the Additional file S1 and S2. A few pathways among those subcategories were highly impacted and evidently induced by the DEG, including ‘Bile secretion’, ‘Fat digestion and absorption’, and ‘Collecting duct acid secretion’. Others were highly impacted but evidently inhibited during lactation such as ‘Endocrine and other factor-regulated calcium reabsorption’ and ‘Proximal tubule bicarbonate reclamation’. All of those pathways had in common a large number of genes coding for proteins involved in ion transport, particularly the ATP pumps for the exchange of Na/K and Ca and Cl export; in addition, ‘Bile secretion’ was composed of genes associated with steroid transport. The overall data analysis indicated a large degree of transport of Na, K, Ca, and Cl. It is difficult to ascertain if those genes code for proteins involved in export or import of those ions inside the epithelial cells (see below for additional results and discussion).

The subcategory ‘Nervous System’ was clearly impacted and induced during lactation (Figure 2). Overall, the data indicated that the nervous system was highly active during lactation. The importance of the nervous system in the mammary gland is well-known mainly for its role in milk ejection [49] but also for its likely control of blood flow [50], both of which are essential for lactation.

### Impact of DEG on Gene Ontology terms and other databases

The KEGG pathways provided a suitable means to interpret the biological phenomena by relying on established metabolic and signaling pathways, but only 35.5% of the genes in our microarray platform were present in the KEGG dataset. A more holistic interpretation of our data could be provided by using Gene Ontology (GO). The GO is made of three categories: Biological process (BP), Molecular function, and Cellular components (CC). The GO terms were associated with >55% of the annotated genes in our microarray. In the following section we only discuss the seemingly-novel findings from the results of DIA analysis of GO and other databases that were not evident from the KEGG analysis. A detailed presentation and discussion of additional findings from GO categories and other databases is reported in Additional file S3. Most of the findings with GO and other databases confirmed KEGG pathway analysis.

#### Novel findings among most impacted Gene Ontology terms

**Figure 3:**
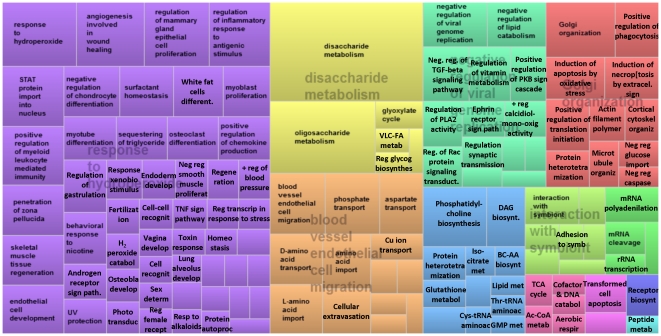
TreeMap visualization obtained by REVIGO analysis (see Materials and Methods for details) of the summary of GO Biological process deemed by DIA to be overall activated during full lactation (from 15 to 120 d) compared to −30 d. Similar colors denote semantic similarity and dimension of the area is proportional to the overall direction of the impact, as calculated by the DIA. Not all the terms are reported due to space constraints (see Additional file S4 for details).

**Figure 4:**
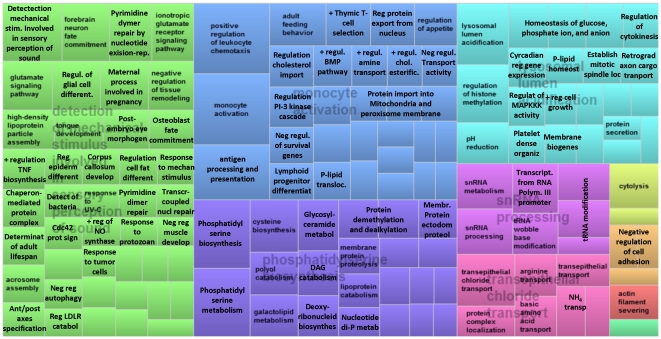
TreeMap visualization obtained by REVIGO analysis (see Materials and Methods for details) of the summary of GO Biological process deemed by DIA to be overall inhibited during full lactation (from 15 to 120 d) compared to −30 d. Similar colors denote semantic similarity and dimension of the area is proportional to the overall direction of the impact, as calculated by the DIA. Not all the terms are reported due to space constraints (see Additional file S4 for details).

The detailed results of the GO analysis are reported in Additional file S1 (“GO Biological process”, “GO Cellular component”, and “GO Molecular function” sheets). We have summarized the data using REVIGO [51] for the interpretation of the main impacted GO terms during lactation (see also Additional file S4). The TreeMap summarizing the direction of the impact in the most impacted categories of terms for the GO-BP are reported in Figure 3 (activated) and Figure 4 (inhibited). Treemap view of the impact for the GO-BP is available in Figure S3 and scatterplot view of the same results is reported in Figure S4 and Figure S5. Results from REVIGO for the GO-CC are shown in Figure S6 and Figure S7 and for GO-MF are reported in Figure S8 and Figure S9 and discussed in Additional file S3.

Among novel findings in GO we observed an induction of several terms related to regulation of inflammation (e.g., ‘Negative regulation of inflammatory response to antigenic stimulus’), transport of several components including amino acids (in particular aspartate and glutamate), phosphate, copper, long-chain fatty acids (LCFA), sodium, and calcium (particularly exocytosis of calcium); proliferation and angiogenesis (e.g., ‘Regulation of mammary gland epithelial cell proliferation’, ‘Angiogenesis involved in wound healing’), Golgi and endoplasmic reticulum (e.g., ‘Golgi organization’, ‘Rough endoplasmic reticulum’), oxidoreductase activity, telomeric region, and epigenetic regulation (i.e,. ‘PgC protein complex’, involved in epigenetic repression of gene expression [52]).Among the novel and most relevant impacted and inhibited terms were several related to immune function (e.g., ‘Positive regulation of leukocyte chemotaxis and monocyte activation’, MHC I), tissue remodeling (including neurons, e.g., ‘Regulation of epidermis differentiation’, ‘Regulation of glial cell differentiation’), tissue sensory capacity including sensing bacteria (e.g., ‘Response to mechanical stimulus’, ‘Detection of bacteria’), chromatin components (e.g., ‘Euchromatin’, ‘Chromocenter’) particularly related to epigenetic modification (e.g., ‘Histone deacetylase complex’), transports of several components such as chloride, arginine, and ammonia, and modification and metabolism of several RNA types (e.g., ‘tRNA modification’, ‘snRNA metabolism’).

Most of the results from GO analysis confirmed previous results from KEGG analysis (see above and Additional file S3) but also provided new insights. For instance, the increased regulation of proliferation suggested that the previously reported decrease in mammary proliferation as lactation begins [45] appeared to be actively regulated at the transcriptional level. The increase in angiogenesis, which also included an increase in ‘Blood vessel endothelial cell migration’ (Additional file S1, ‘GO Biological process’ sheet), suggested an important role of angiogenesis during lactation in bovine mammary gland with a continuous formation of blood vessels. The blood flow across the mammary gland increases dramatically at the onset of lactation [53] with a consistent increase during the first 3 months of lactation, particularly for multiparous cows [54]. It has been well-established that there is a positive correlation between blood flow and milk yield [53]; however, this appears to be true only for cows with less than 3 lactations [54].

The DIA results of our microarray data clearly indicated an inhibition of the immune activation in the mammary gland. This finding, also supported by the results from the DIA analysis of KEGG pathways (see above), is quite striking. In modern high-producing cows, the importance of the immune response in mammary gland is pivotal due to the need for preventing/fighting pathogen-causing mastitis [55]. Our data highlighted several unique aspects of mammary tissue:

- The large inhibition of immune-related functions, particularly MHC class I, together with the predominance of epithelial cells in our core biopsies (see companion paper) indicated that the mammary gland is actively involved in the immune system and supports the suggestion of the mammary gland being an evolutionary product of the innate immune system [56];
- The mammary gland during lactation has an apparent increase in activity/preparation of the immune system, particularly the innate immune system (see above and Additional file S1, sheet ‘KEGG pathway’), but there was a decrease in chemotaxis and activity of immune cells. The infiltration of immune cells, particularly macrophages, into mammary tissue is essential for the initiation and resolution of an inflammatory response [57]. Our data suggested increased accumulation of macrophages or immune cells in general as suggested by the ‘Hematopoietic cell lineage’ (Additional file S1 and S2) and specific immune cell markers (Figure S10). Those data were indicative of a more pronounced increase in macrophage infiltration between 1 through 60 d when it reached a peak, followed by a subsequent decrease. The increase in number of macrophages in mammary tissue during the surge in milk production was similar to observations reported for the rat [58]. In contrast, neutrophil infiltration seemed to increase through the end of lactation (Figure S10);
- Another striking finding was the consistent decrease of the major histocompatibility complex (MHC), which is similar to microarray data of mouse mammary gland during lactation [4] despite the fact that the MHC class II in mouse is not expressed in epithelial cells [59] as it is in bovine [60]. The MHC class I is present in all nucleated cells and it is the mechanism whereby all cells participate in the immune defense, presenting antigens from bacteria and virus to CD8+ T-lymphocytes [61]. An association between Class I alleles and mastitis traits has been reported [62]. The mRNA expression of bovine mammary MHC components was increased due to intramammary challenge with *S. Uberis* strain O140J [63], probably due to the epithelial response, as previously demonstrated *in vitro* [60].

Overall, those data confirmed the lack of mastitis in the mammary tissue used in the present investigation. The reason for the decrease of the MHC pathway, particularly class I, is not readily apparent because the mammary gland is an easily accessible site of bacterial entry which in turn requires a rapid immune response. Under such scenario we propose two hypotheses (see also [64]): 1) genetic selection of cattle for high milk production, which has brought about an increase in mastitis incidence [65], might have inadvertently reduced the expression of the MHC during lactation resulting in lower sensitivity to bacteria and a consequent decrease in energy expenditure for immune responses; 2) the MHC is a vesicle-dependent process which uses ER-Golgi networks [61] as do milk components; thus, the MHC can be considered a competitor of the vesicle-transport system. The MHC inhibition can be considered a way for the mammocytes to spare resources.

The GO results indicated an inhibition of the active components of the epigenetic modification and increase of long term epigenetic stabilizer (e.g., PcG protein complex, [52]). Those data highlighted the seemingly crucial importance of epigenetic regulation in bovine mammary, thus, support recent findings [66]. In particular, the data suggested that bovine mammary once the euchromatic status is established tends to inhibit any epigenetic modification as a means to maintain a consistent transcriptome until milk synthesis declines. This idea is supported by the number of DEG reported in Figure 1, where very few DEG were observed in the comparison between consecutive time points from 15 to 120 d postpartum, but a large number of DEG was observed when milk yield declined (between 120 and 240 d).

### Results from the analysis of additional annotation databases

Among SP_PIR_Keywords (Additional file S1), unique responses were the high impact and activation during lactation of the terms: ‘Molibdenum’, whose importance is supported by the fact that it has been found to be the most transferred microelement in human milk [67]; ‘Antimicrobial’ likely related to the increased expression of several proteins related to the innate immune system (e.g., osteopontin, *XDH* [56]); ‘Growth arrest’ supporting the above conclusion that the regulation of cell growth is actively under transcriptomics control in mammary; and ‘Protein synthesis inhibitor’, which suggested that the decrease in protein synthesis machinery during lactation is an important and tightly regulated phenomena as also discussed above and in a previous work [7].

Besides the terms known to be related to lactating mammary, additional findings from the DIA analysis of other databases (Additional file S1) were the large induction during lactation of several groups of proteins/domains besides caseins and lactoalbumin: ‘Synaptotagmin’, ‘Sad1/UNC-like, C-terminal’, ‘Protein of unknown function DUF775’, ‘Na/Pi-cotransporter’, ‘Spermadhesin’, and ‘Parathyroid hormone-related proteins’. A thorough discussion of those terms is beyond the scope of this manuscript; however, parathyroid hormone has been shown to be involved in the regulation of capillary blood flow [68] and regulation of calcium influx [69] in mammary tissue, the large importance of Na/Pi-cotransporter revealed by the DIA analysis confirms its crucial role in transport of Pi into mammary gland [70]. For the other proteins there is no available information in bovine mammary gland and our data prompt for additional molecular work to clarify their specific roles during lactation. Among those the synaptogamin appears quite interesting. A physical association between this protein with glutathione S-transferases has been found in breast cancer cells [71]. In addition, synaptogamin proteins are involved in intracellular membrane trafficking, particularly linking calcium influx to synaptic exocytosis in neurons [72] and are involved in the membrane fusion through SNARE [73]. Even though those are neuron-specific functions our data point to an important role of synaptogamin in mammary tissue, perhaps in the vesicle trafficking during secretion of milk components.

Lastly, the DIA analysis of COGontology uncovered as the most impacted terms ‘Amino acid transport and metabolism’ (overall induced during lactation) and ‘Chromatin structure and dynamics/Transcription’ (overall inhibited during lactation). Those findings further support the pivotal importance of AA for lactating mammary and the epigenetic stasis (i.e., inhibition of chromatin modifications) of the mammary during lactation as discussed above.

### Limitations of the study

The interpretation of the findings from the present study has several limitations. For instance, the microarray platform used covers less than 50% of the entire annotated bovine genome and no more than 60% of the genes in the bovine genome have a functional annotation. In addition, despite the fact that >80% of microarray results were confirmed using qPCR (Additional file S5) it is likely that ca. 16% of the data used for the analysis are inaccurate.

### Summary and conclusions

**Figure 5:**
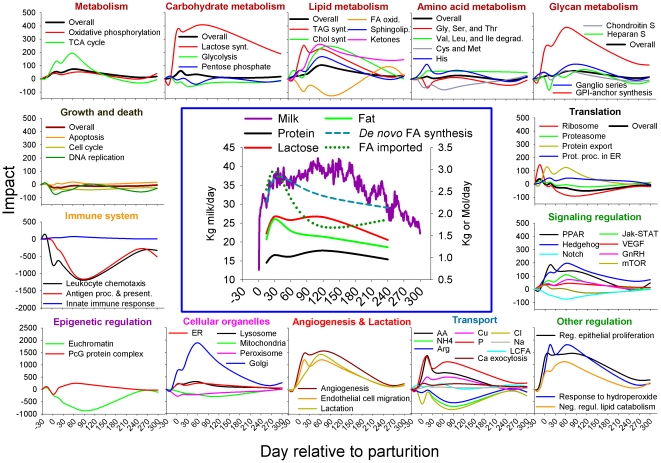
Summary figure of the most relevant biological functions in bovine mammary gland during the lactation cycle as revealed by the Dynamic Impact Approach (DIA) analysis of KEGG pathways and Gene Ontology terms (see Additional file S1 for details). Shown at the center are the curve of lactation, yield of main milk constituents (protein, lactose, and fat) [7], [8], and estimates of fatty acid import and *de novo* synthesis [8]. Red titles are the most relevant metabolism as revealed by the KEGG pathway and Gene Ontology analysis. Other color titles denote main functions impacted during lactation. The large increase at the transcriptomic level of caseins or other milk proteins are not shown. The X-axis depicts the day relative to parturition and the Y-axis the direction of the impact as calculated by DIA. Legend: AA = amino acid; Arg = arginine; Ca = calcium; Chol = cholesterol; Cl = chloride; Cu = copper; Cys = cysteine; ER = endoplasmic reticulum; FA = fatty acids; GnRH = Gonadotropin-releasing hormone pathway; Gly = glycine; GPI = Glycophosphatidylinositol; His = histidine; Ile = isoleucine; LCFA = long-chain fatty acids; Leu = leucine; Met = methionine; mTOR = mTOR = mammalian target of rapamycin signaling pathway; Na = sodium; NH4 = ammonium; Notch = Notch signaling pathway; P = phosphate; PPAR = peroxisome proliferator-activated receptor signaling pathway; S = sulfate; Ser = serine; TAG = triacylglycerol; Thr = threonine; Val = valine; VEGF = vascular endothelial growth factor signaling pathway.

Figure 5 summarizes the main findings of the present study using the DIA. Among the most relevant biological phenomena in lactating bovine mammary our findings suggest based on KEGG pathways analysis:

- an overall increase in mammary metabolism with large increase in anabolism, particularly involving glucose and lipids (likely LCFA and cholesterol) but also energy production with an increase in utilization of amino acids as an energy source for the TCA cycle. The synthesis of glycan was increased overall during lactation, particularly the GPI-anchor, which might have a role in milk secretion;
- the protein synthesis machinery, together with the metabolism of RNA and DNA, appeared to be inhibited during lactation but the export of proteins was increased as well as the transport of sterols;
- mammary cells appeared to have increased formation of junctions, particularly tight junctions, and the cellular signaling cascades during lactation, particularly through the Hedgehog and Jak-STAT pathways;
- the cell cycle as well as apoptosis were inhibited overall during lactation but slightly resumed when lactation declined;
- central to mammary tissue adaptations to lactation was PPAR signaling, mostly likely through PPARγ. Besides PPAR the GnRH and the Hedgehog signaling pathways also appeared to be important, but their functional role remains unclear. The DIA also suggests an overall inhibition of mTOR and Notch signaling pathways;
- the mammary gland appeared to have placed substantial effort in preparing the immune system but also prevented oversensitivity to bacteria/virus invasion through a large inhibition of MHC components expression;

The analysis of GO and other databases confirmed most of the findings with KEGG but also highlighted the importance of lactose synthesis, Golgi, angiogenesis, negative regulation of lipid catabolism, transport of AA, phosphate and other minerals, and epigenetic regulation. For the latter, the data suggested a general inhibition of chromatin modification during lactation.

The present transcriptome analysis provided insights into the biological adaptation of the bovine mammary gland during end of pregnancy and during the whole lactation. The data highlighted a strong transcriptional regulation of metabolism (particularly lipid and glucose), immune response, epigenetics, protein synthesis machinery, angiogenesis, and transport among others. From a practical standpoint the discovery of which factors regulate such adaptation is of even higher relevance. Few regulatory pathways appear to play a pivotal role in such adaptations, e.g. PPAR and Hedgehog signaling, but it remains to be determined which transcription factors or networks of transcription factors are controlling this orchestrated and dynamic adaptations to allow mammary cells to synthesize and secrete milk.

## Materials and Methods

### Ethics Statement

All procedures for animal handling prior to and after mammary biopsy were conducted under protocols approved by the University of Illinois Institutional Animal Care and Use Committee under protocol 04117.

### Animals and sampling

Eight multiparous Holstein dairy cows from the University of Illinois Dairy Cattle Research Unit were used. Percutaneous biopsies were obtained at −30 (−28±3 days; n = 7), −15 (−13±3 days; n = 8), 1 (n = 8), 15 (n = 8), 30 (n = 8), 60 (n = 6), 120 (n = 6), 240 (n = 5) and 300 (n = 5) days relative to parturition (d) as previously described [30].

### RNA extraction, Microarray, qPCR, Milk yield and Milk Composition Analyses

The details for RNA extraction, microarray, qPCR protocol, and milk yield and composition are reported in Additional file S3. Briefly, we used an annotated bovine oligonucleotide microarray containing >10,000 unique elements [74]. Hybridizations were performed in a dye-swap reference design. The microarray data presented in this manuscript have been deposited at NCBI's Gene Expression Omnibus [75] and are accessible through GEO Series accession number GSE19055.

### Statistical analysis

Data from a total of 120 microarrays were normalized for dye and array effects (i.e., Lowess normalization and array centering and scaling) before statistical analysis. As previously described [74], a mixed effects model was fitted to the adjusted ratios (mammary/reference) of each oligonucleotide using Proc MIXED (SAS Inst., Cary, NC). The model consisted of the classification factors time (i.e., −30, −15, 1, 15, 30, 60, 120, 240, and 300 d) and dye as fixed effect, and cow as a random variable. The overall effect of time and pair-wise comparisons among time points were computed. The mean of the two spots for each oligonucleotide within each array and between dye-swap arrays was not averaged prior statistical analysis. The significant probability values for the time effect were adjusted for the number of comparisons using Benjamini and Hochberg's false discovery rate (FDR) [76]. Data from statistical analysis were further filtered to obtain highly-reliable data. The filter was based on the number of observations available to test for time effect (i.e., interaction). Results from oligonucleotides with more than 93% of the data are discussed (≥40 out of 43 degrees of freedom). This criterion resulted in the elimination of results from 17.7% (2,345) of the total number of oligonucleotides analyzed.

### Dynamic Impact Approach for data mining

The description of the rationale, procedures, criteria, and validation of the Dynamic Impact Approach or DIA are reported in the companion paper.

### KEGG pathway visualization

KEGG pathway visualization for the comparison 60 vs. −30 d was performed using the application KeggArray available in KEGG (Kyoto Encyclopedia of Genes and Genomes) website at http://www.genome.jp/kegg/download/kegtools.html. The results from analysis using KeggArray is not fully comparable with the DIA because the former only uses the fold change as input while the DIA accounts for the proportion of DEG compared to the genes present in the microarray, the P-value of the change, and the fold change. See Additional file S2 for additional details. The images are shown to help in data interpretation but should be used with caution.

### Gene Ontology result visualization

The use of DIA results in a large number of terms for the Gene Ontology (GO) analysis, particularly in the Biological process category. This can render the interpretation of data very difficult also due to a redundancy of similar terms. To provide a more comprehensive interpretation and visualization of GO data we have used the freely available web-based software REVIGO at http://revigo.irb.hr/index.jsp [51]. See Additional file S3 for details.

## Supporting Information

Figure S1 **Curve of lactation (mean kg milk yield/day) during the 300 day lactation in the 8 Holstein cows used for mammary biopsies and microarray analysis.**
(TIF)

Figure S2 **Number of DEG with FDR≤0.001 and post-hoc P<0.001 in each time point relative to −30 d (pregnancy) and in each time point relative to the previous time point (e.g., 30 = 30 vs. 15 d, 120 = 120 vs. 60 d).** Several fold-change thresholds were applied (1.3 = 30% change; 1.5 = 50% change) which highlighted the increase in the proportion of up-regulated genes when a greater fold-change threshold was applied.
(TIF)

Figure S3 **TreeMap results from REVIGO of GO Biological process terms induced (A) and inhibited (B) during lactation (from 15 to 120 vs. −30 d).** Shown are the results of **the impact**. The size of the shape denotes overall impact (the larger the size the greater the impact). Similar colors denote semantic similarity. See detailed table in Additional file S4.
(TIF)

Figure S4 **Scatterplot results from REVIGO of GO Biological process terms induced (A) and inhibited (B) during lactation (from 15 to 120 vs. −30 d).** Shown are the results of the **direction of the impact**. The size and color of the bubbles denote overall direction of the impact (from dark blue to red = larger direction of the impact), and the larger the size the greater the activation in the A panel and inhibition in the B panel. See detailed table in Additional file S4.
(TIF)

Figure S5 **Scatterplot results from REVIGO of GO Biological process terms induced (A) and inhibited (B) during lactation (from 15 to 120 vs. −30 d).** Shown are the results of **the impact**. The size and color of the bubbles denote overall impact (from dark blue to red = larger impact), and the larger the size the greater the impact. See detailed table in Additional file S4.
(TIF)

Figure S6 **Scatterplot results from REVIGO of GO Cellular Components terms induced (A) and inhibited (B) during lactation (from 15 to 120 vs. −30 d).** Shown are the results of the **direction of the impact**. The size and color of the bubbles denote the overall direction of the impact (from dark blue to red = larger direction of the impact), and the larger the size and color from blue to red greater the activation in the upper panel; larger the size and color from red to blue greater the inhibition in the lower panel. See detailed table in Additional file S4.
(TIF)

Figure S7 **TreeMap results from REVIGO of GO Cellular component terms induced (A) and inhibited (B) during lactation (from 15 to 120 vs. −30 d).** Shown are the results of **direction of the impact**. The size of the shape denotes the overall impact (larger the size greater the impact). Similar colors denote semantic similarity. See detailed table in Additional file S4.
(TIF)

Figure S8 **Scatterplot results from REVIGO of GO Molecular function terms induced (A) and inhibited (B) during lactation (from 15 to 120 vs. −30 d).** Shown are the results of the **direction of the impact**. The size and color of the bubbles denote the overall direction of the impact (from dark blue to red = larger direction of the impact), and the larger the size and color from blue to red greater the activation in the upper panel; larger the size and color from red to blue greater the inhibition in the lower panel. See detailed table in Additional file S4.
(TIF)

Figure S9 **TreeMap results from REVIGO of GO Molecular function terms induced (A) and inhibited (B) during lactation (from 15 to 120 vs. −30 d).** Shown are the results of **the direction of the impact**. Size of the shape denotes overall impact (larger the size larger the impact). Similar colors denote semantic similarity. See detailed table in Additional file S4.
(TIF)

Figure S10 **Ratio expression of specific macrophage and neutrophil markers (from** http://www.antibodybeyond.com/index.htm **) in each time point relative to −30 d in bovine mammary tissue during lactation.** In the image are reported the specific markers present in the bovine oligo microarray which were significantly affected by time at an FDR≤0.001. The symbols denote: monocyte differentiation antigen CD14 (CD14), integrin, alpha X (complement component 3 receptor 4 subunit or leukocyte surface antigen p150,95, alpha subunit) (ITGAX), lactotransferrin (LTF), elastase 2 neutrophil (ELA2), transforming growth factor beta 2 (TGFB2), and matrix metallopeptidase 2 (MMP2). Image generated with GeneSpring GX7.
(TIF)

File S1 **Complete results from the time course experiment using Dynamic Impact Approach (DIA) for DEG between each time point relative to −30 d and comparison between consecutive time points.** Reported are the results from the KEGG pathways, Gene Ontology (Biological process, Cellular component, and Molecular function), SP PIR Keyword, Interpro, COGontology, PIRsuperfamily, SMART, SSF, and UP_Seq_Feature.
(XLSX)

File S2 **Visualization of KEGG pathways for the comparison 60 vs. −30 d using the application KeggArray available in KEGG: Kyoto Encyclopedia of Genes and Genomes website at** http://www.genome.jp/kegg/download/kegtools.html **.**
(DOCX)

File S3 **Additional Materials & Methods, results, and discussion.**
(DOC)

File S4 **Tabulated results from the REVIGO analysis of Gene Ontology terms using output from the DIA as described in Additional file S3.**
(XLSX)

File S5 **Tabulated results of microarray** ***vs.*** **quantitative RT-PCR data for 89 genes.**
(XLSX)
