# Supplementary figures and images for: Old and New Stories: Revelations from Functional Analysis of the Bovine Mammary Transcriptome during the Lactation Cycle
Source: PLoS One. 2012 Mar 12;7(3):e33268. doi: 10.1371/journal.pone.0033268 (PMC3299771; doi:10.1371/journal.pone.0033268)

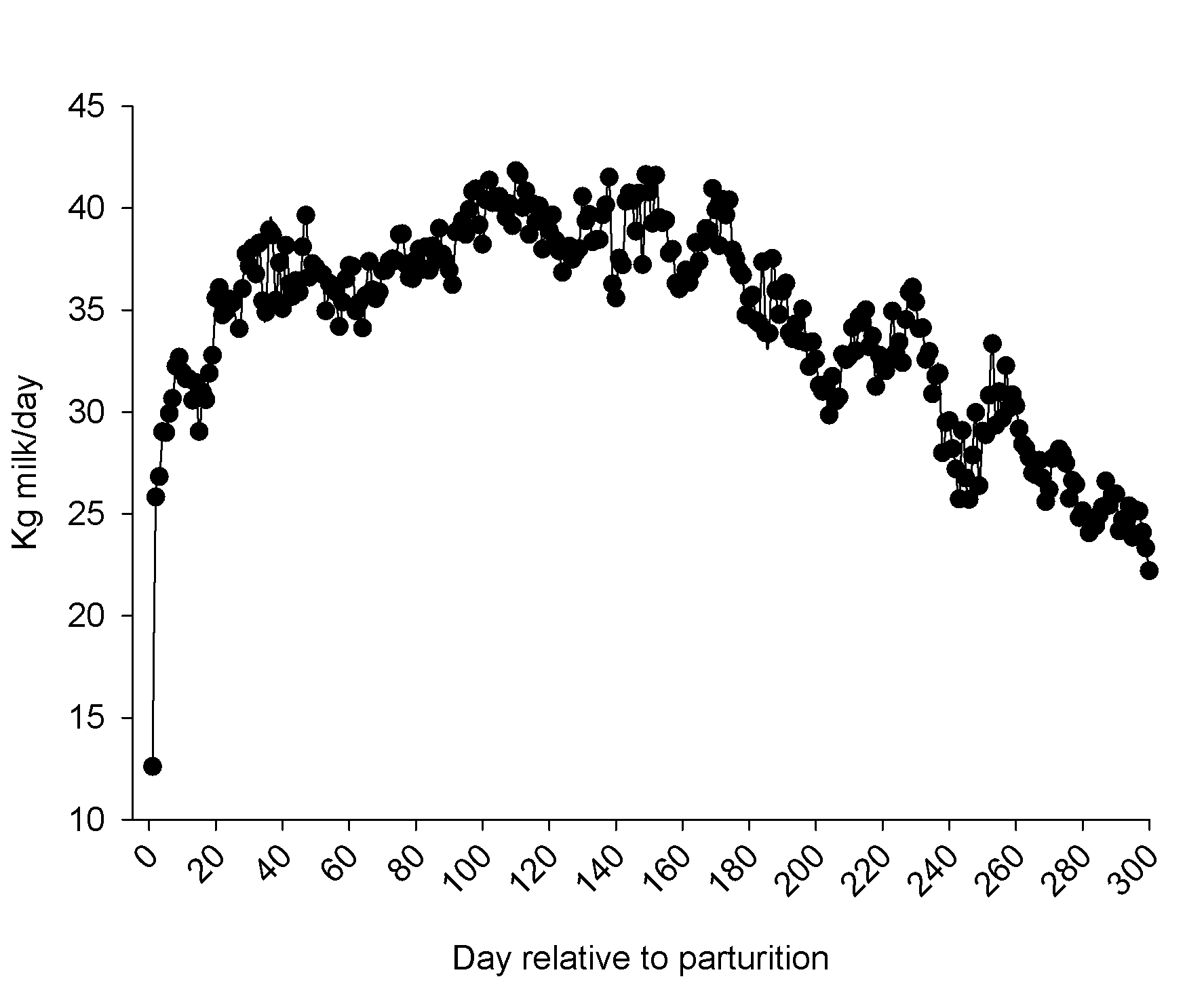

Supplement: Figure S1 — Curve of lactation (mean kg milk yield/day) during the 300 day lactation in the 8 Holstein cows used for mammary biopsies and microarray analysis. (TIF) [file pone.0033268.s001.tif]

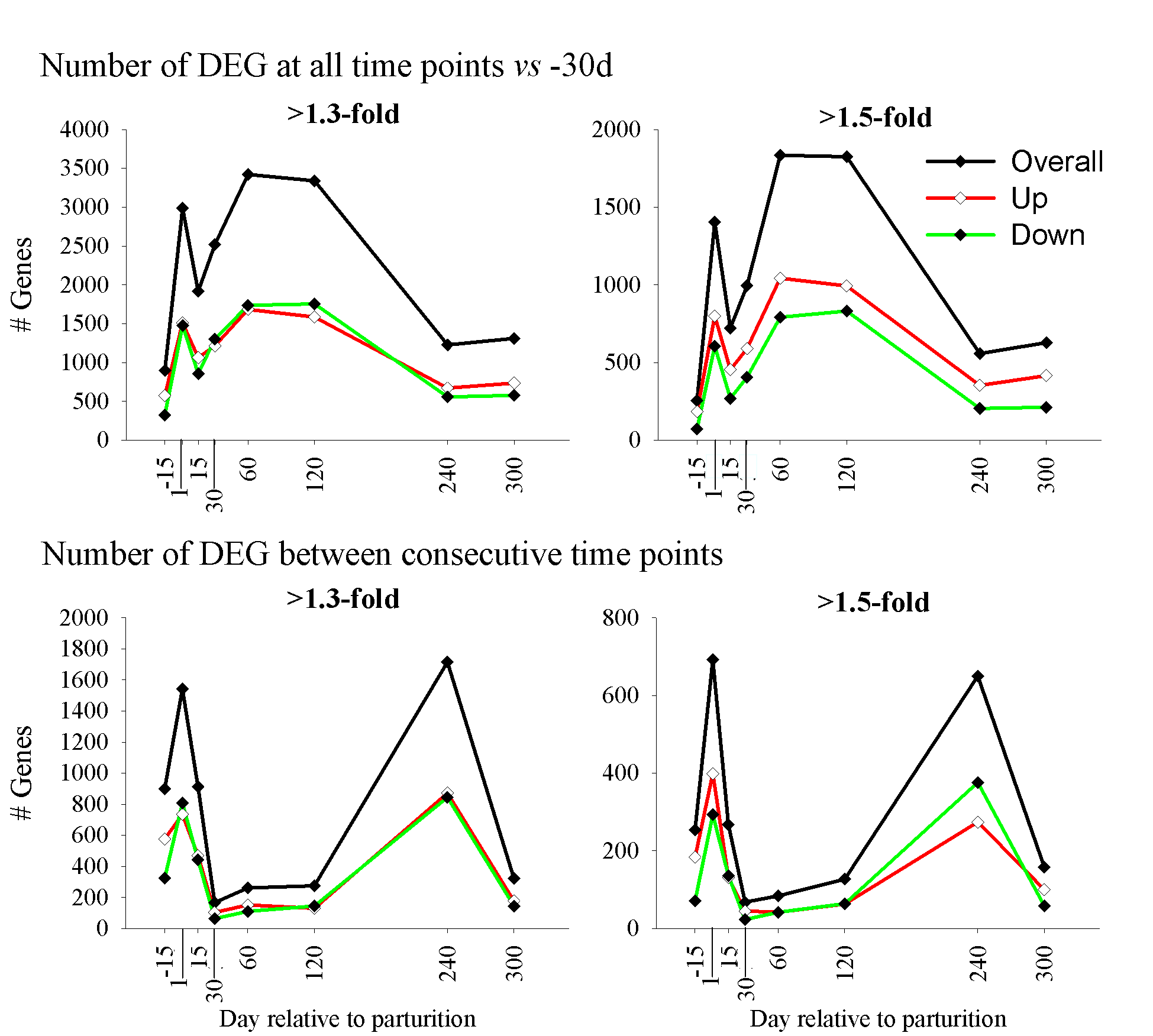

Supplement: Figure S2 — Number of DEG with FDR≤0.001 and post-hoc P<0.001 in each time point relative to −30 d (pregnancy) and in each time point relative to the previous time point (e.g., 30 = 30 vs. 15 d, 120 = 120 vs. 60 d). Several fold-change thresholds were applied (1.3 = 30% change; 1.5 = 50% change) which highlighted the increase in the proportion of up-regulated genes when a greater fold-change threshold was applied. (TIF) [file pone.0033268.s002.tif]

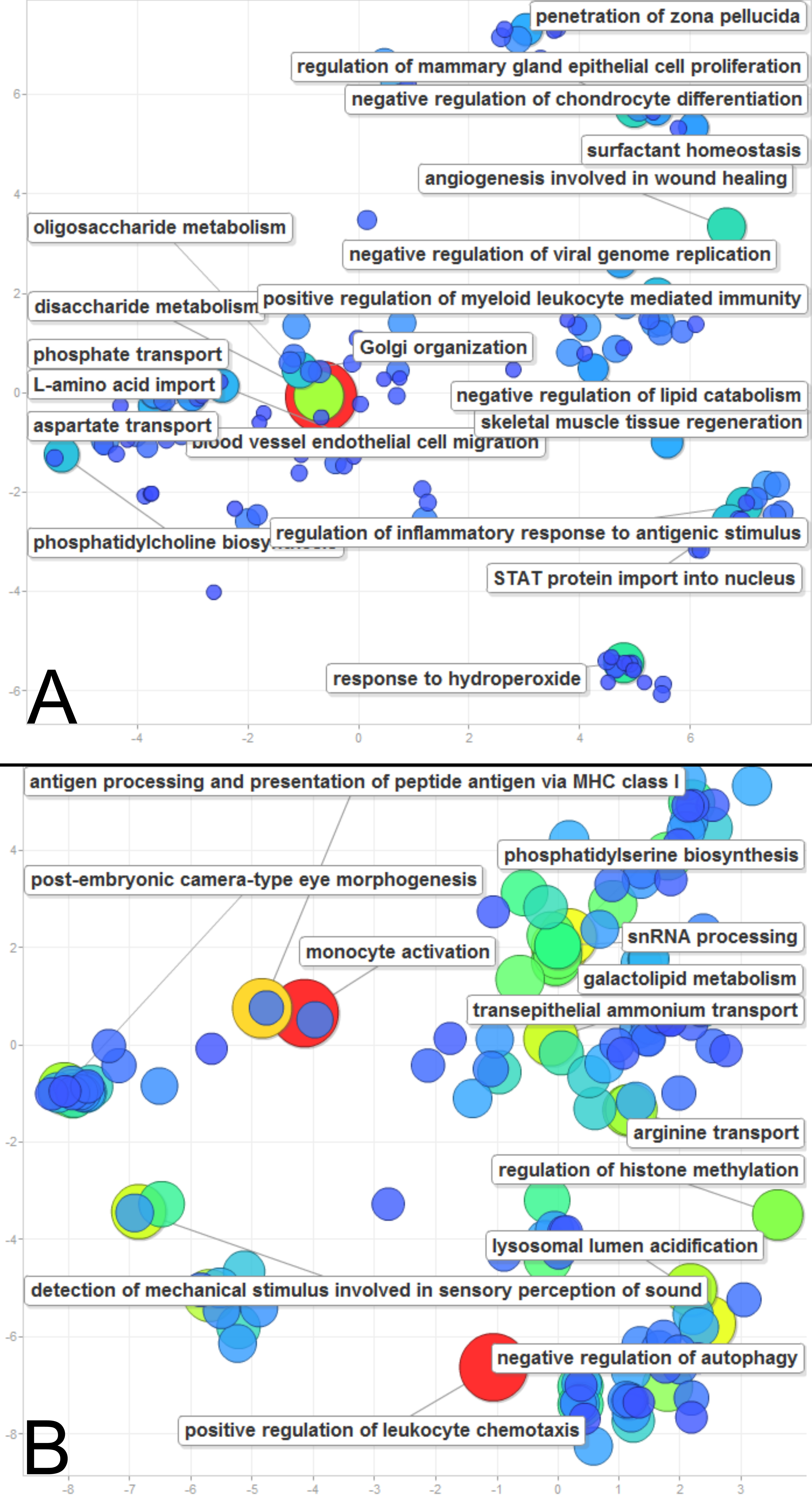

Supplement: Figure S3 — TreeMap results from REVIGO of GO Biological process terms induced (A) and inhibited (B) during lactation (from 15 to 120 vs. −30 d). Shown are the results of the impact. The size of the shape denotes overall impact (the larger the size the greater the impact). Similar colors denote semantic similarity. See detailed table in Additional file S4. (TIF) [file pone.0033268.s003.tif]

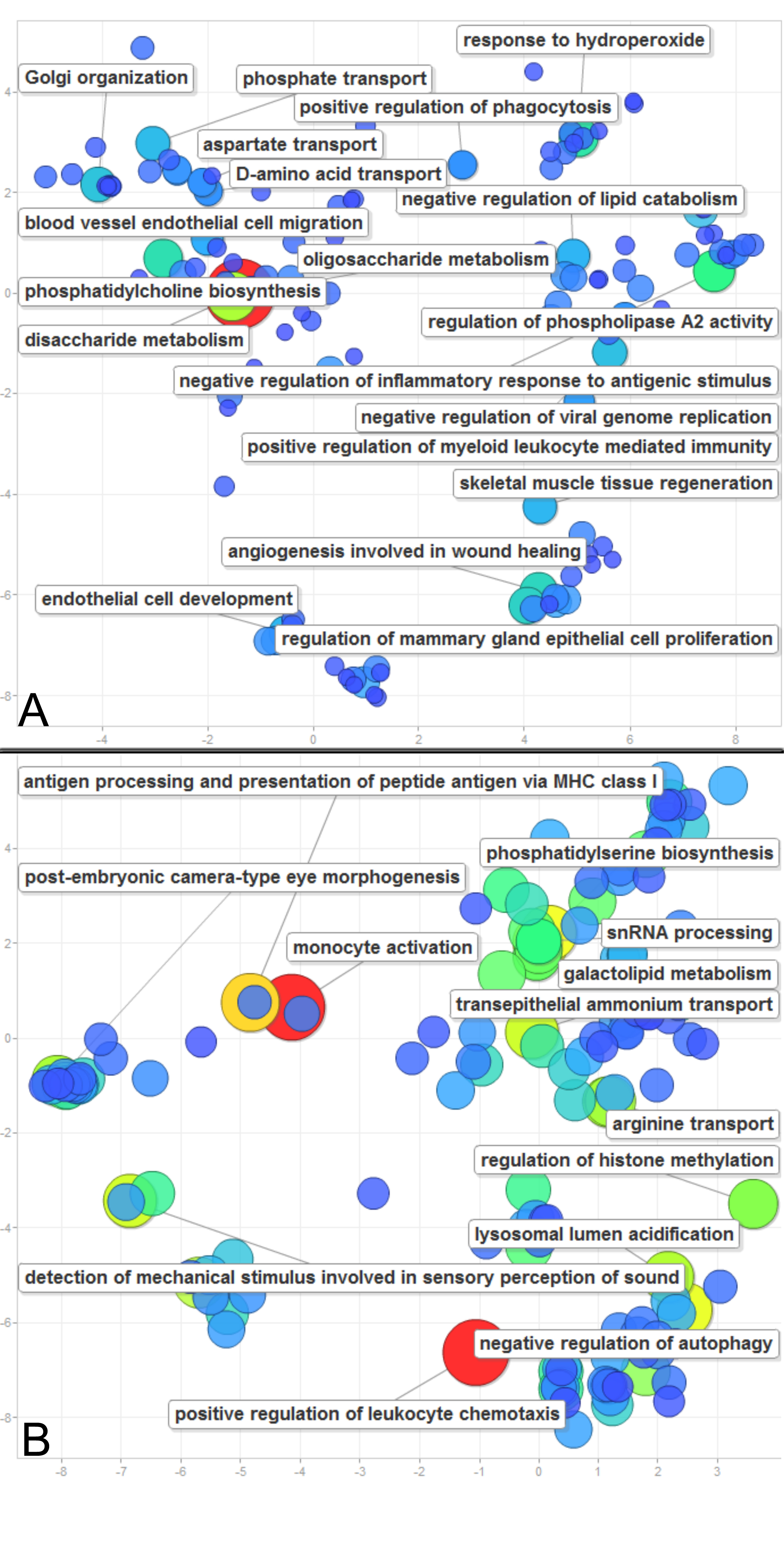

Supplement: Figure S4 — Scatterplot results from REVIGO of GO Biological process terms induced (A) and inhibited (B) during lactation (from 15 to 120 vs. −30 d). Shown are the results of the direction of the impact. The size and color of the bubbles denote overall direction of the impact (from dark blue to red = larger direction of the impact), and the larger the size the greater the activation in the A panel and inhibition in the B panel. See detailed table in Additional file S4. (TIF) [file pone.0033268.s004.tif]

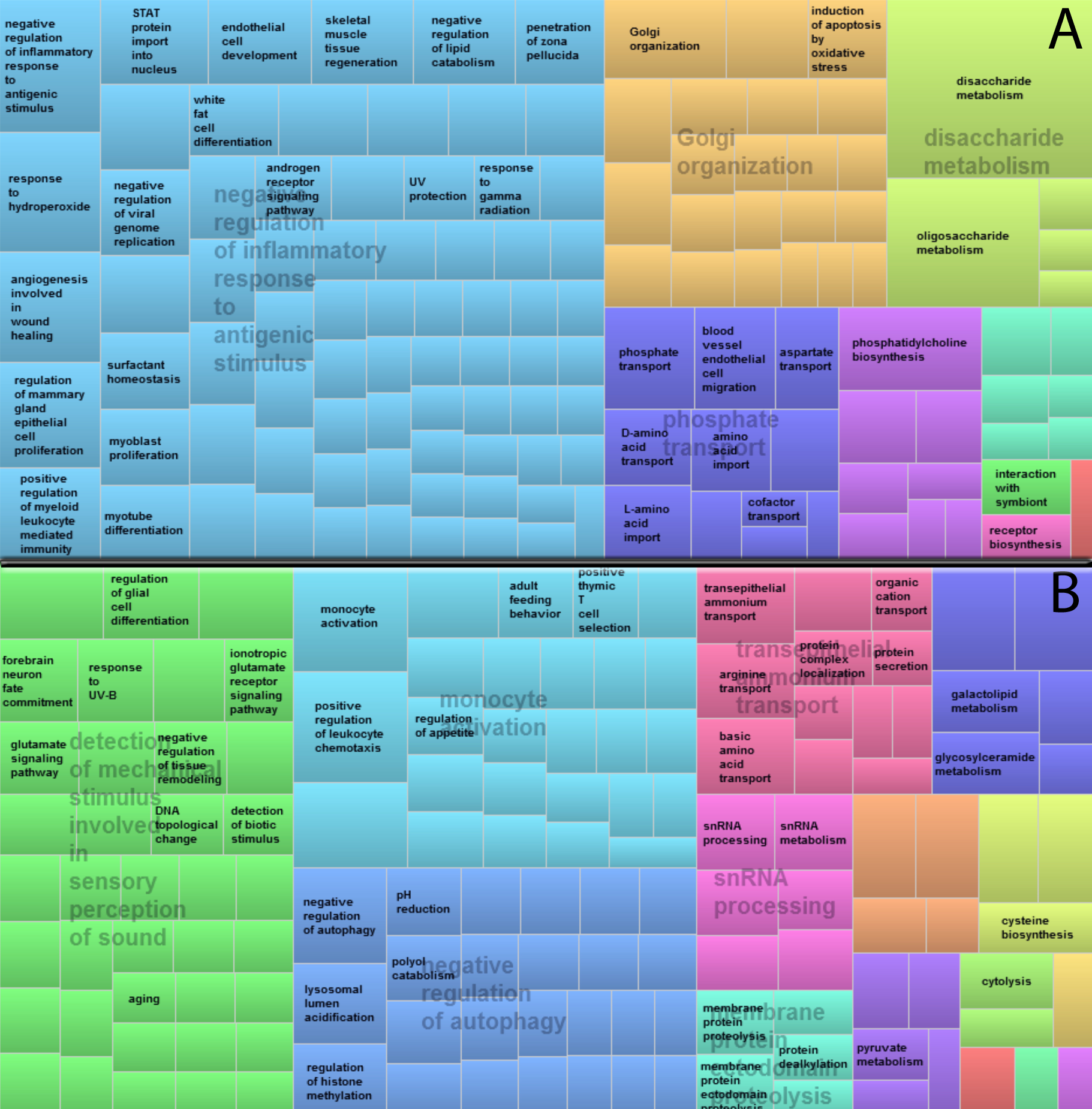

Supplement: Figure S5 — Scatterplot results from REVIGO of GO Biological process terms induced (A) and inhibited (B) during lactation (from 15 to 120 vs. −30 d). Shown are the results of the impact. The size and color of the bubbles denote overall impact (from dark blue to red = larger impact), and the larger the size the greater the impact. See detailed table in Additional file S4. (TIF) [file pone.0033268.s005.tif]

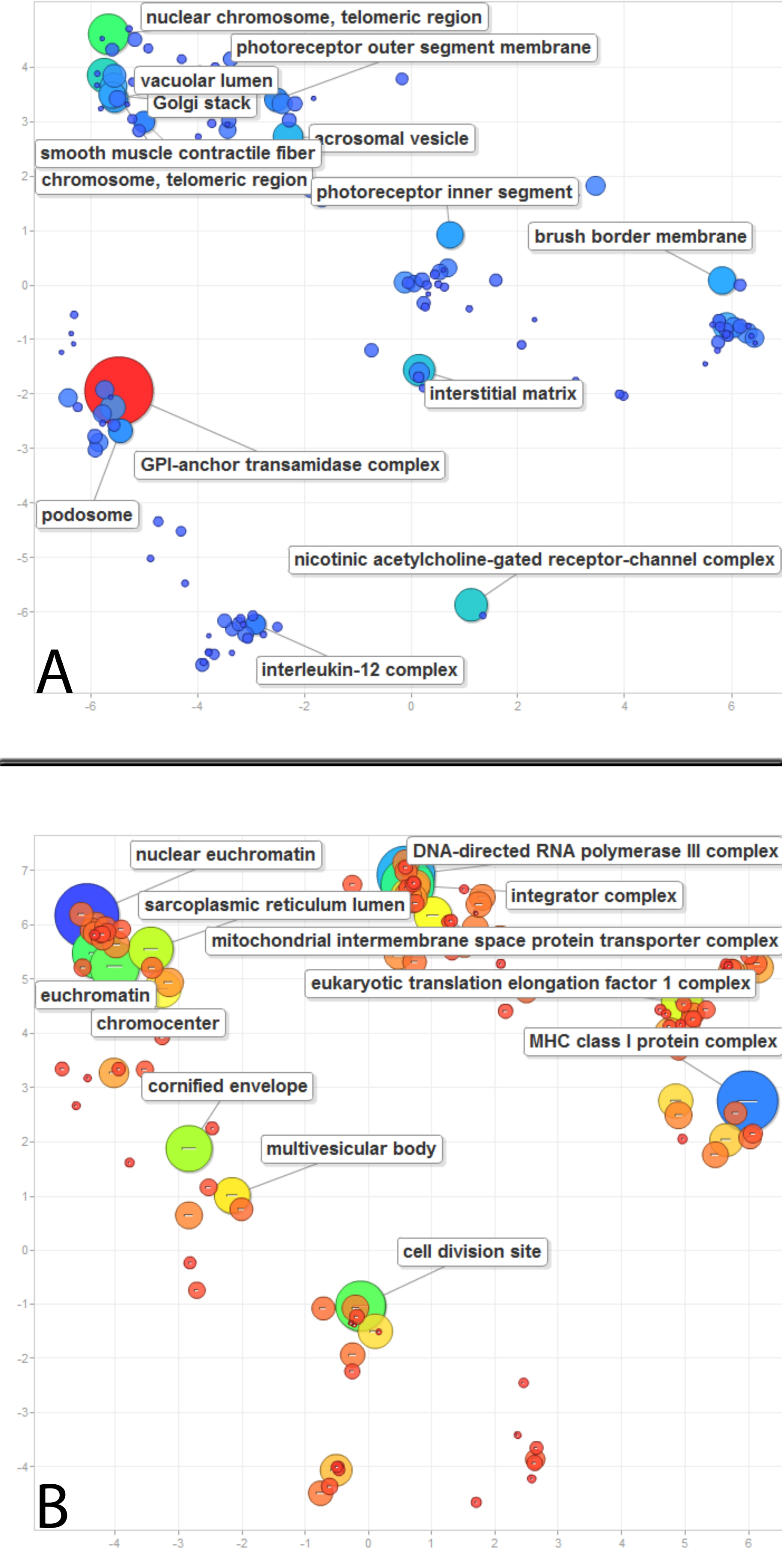

Supplement: Figure S6 — Scatterplot results from REVIGO of GO Cellular Components terms induced (A) and inhibited (B) during lactation (from 15 to 120 vs. −30 d). Shown are the results of the direction of the impact. The size and color of the bubbles denote the overall direction of the impact (from dark blue to red = larger direction of the impact), and the larger the size and color from blue to red greater the activation in the upper panel; larger the size and color from red to blue greater the inhibition in the lower panel. See detailed table in Additional file S4. (TIF) [file pone.0033268.s006.tif]

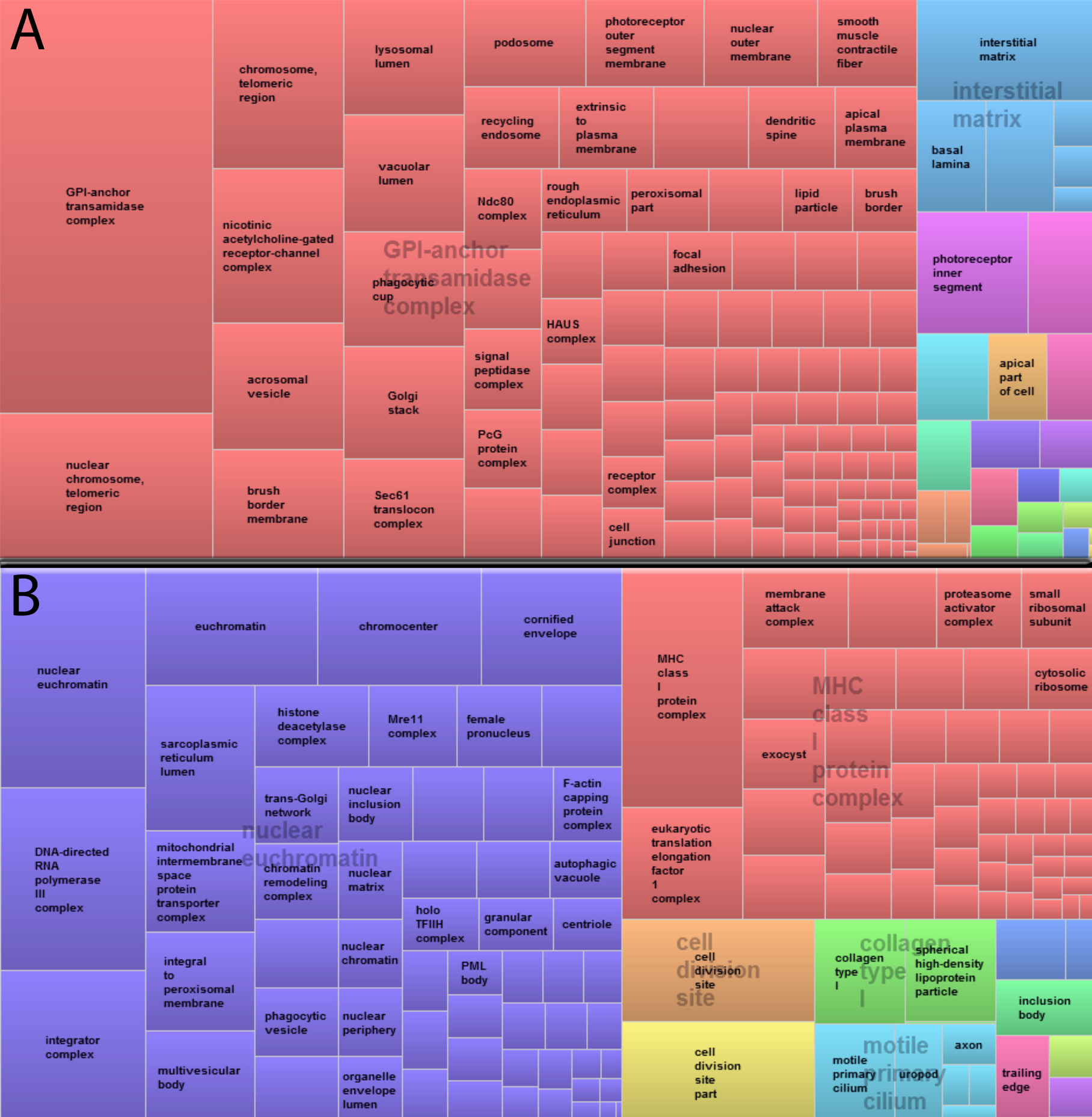

Supplement: Figure S7 — TreeMap results from REVIGO of GO Cellular component terms induced (A) and inhibited (B) during lactation (from 15 to 120 vs. −30 d). Shown are the results of direction of the impact. The size of the shape denotes the overall impact (larger the size greater the impact). Similar colors denote semantic similarity. See detailed table in Additional file S4. (TIF) [file pone.0033268.s007.tif]

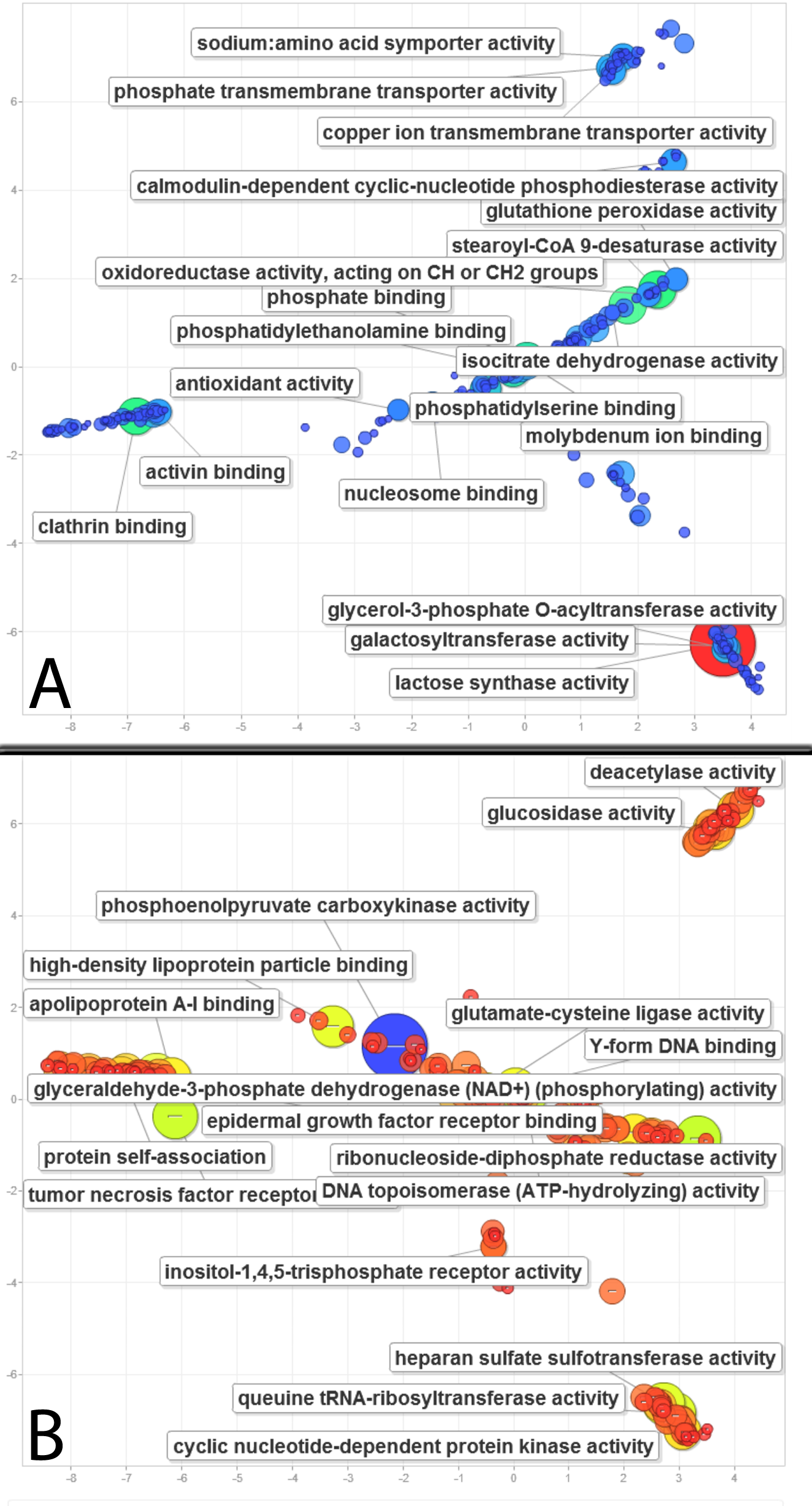

Supplement: Figure S8 — Scatterplot results from REVIGO of GO Molecular function terms induced (A) and inhibited (B) during lactation (from 15 to 120 vs. −30 d). Shown are the results of the direction of the impact. The size and color of the bubbles denote the overall direction of the impact (from dark blue to red = larger direction of the impact), and the larger the size and color from blue to red greater the activation in the upper panel; larger the size and color from red to blue greater the inhibition in the lower panel. See detailed table in Additional file S4. (TIF) [file pone.0033268.s008.tif]

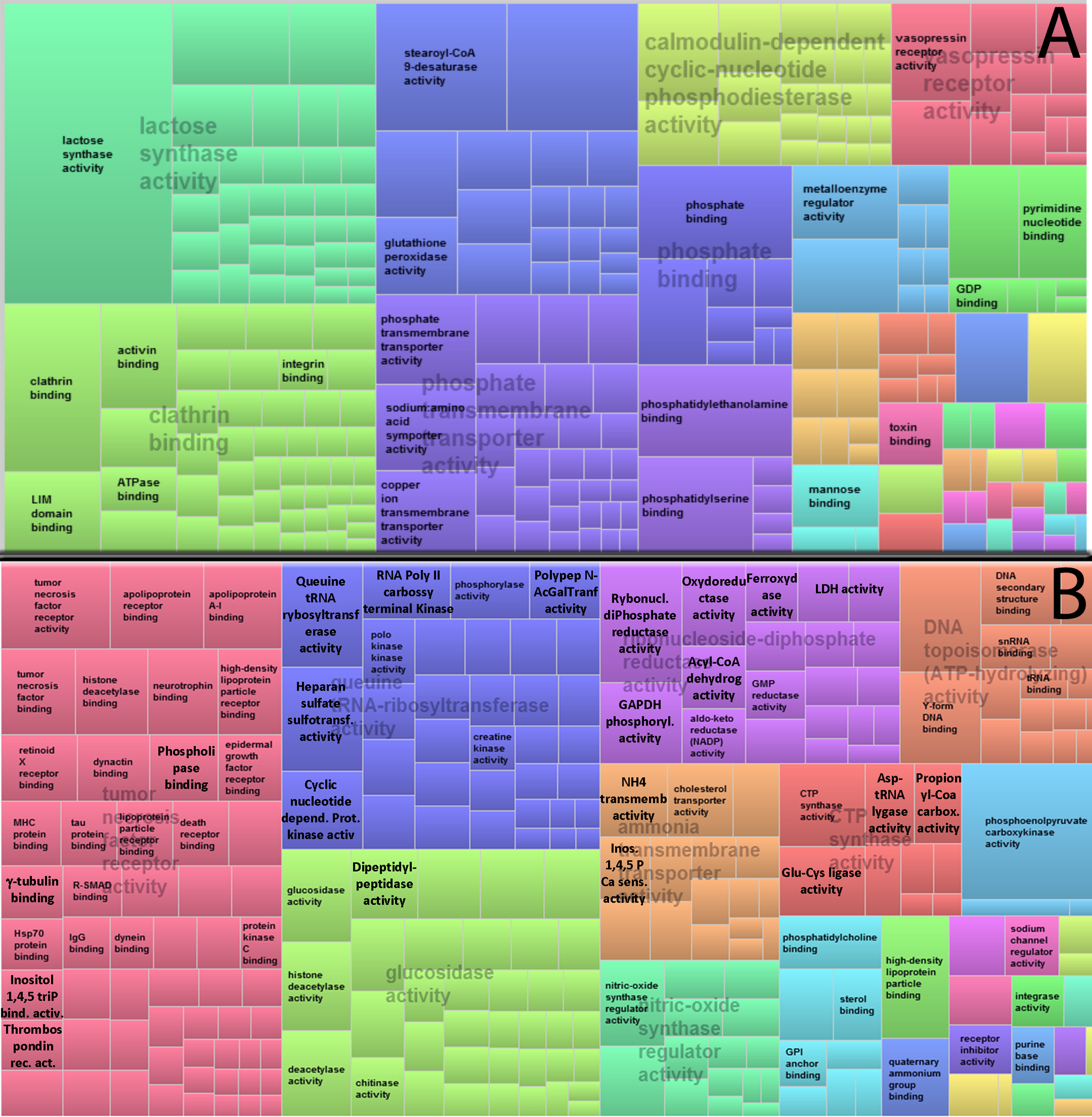

Supplement: Figure S9 — TreeMap results from REVIGO of GO Molecular function terms induced (A) and inhibited (B) during lactation (from 15 to 120 vs. −30 d). Shown are the results of the direction of the impact. Size of the shape denotes overall impact (larger the size larger the impact). Similar colors denote semantic similarity. See detailed table in Additional file S4. (TIF) [file pone.0033268.s009.tif]
