# Supplementary material for: Old and New Stories: Revelations from Functional Analysis of the Bovine Mammary Transcriptome during the Lactation Cycle
Source: PLoS One. 2012 Mar 12;7(3):e33268. doi: 10.1371/journal.pone.0033268 (PMC3299771; doi:10.1371/journal.pone.0033268)
Supplement: File S2 — Visualization of KEGG pathways for the comparison 60 vs. −30 d using the application KeggArray available in KEGG: Kyoto Encyclopedia of Genes and Genomes website at http://www.genome.jp/kegg/download/kegtools.html . (DOCX) [file pone.0033268.s012.docx]

**VISUALIZATION OF KEGG PATHWAYS**

KEGG pathways visualization for the **comparison 60 vs. -30d** using the application **KeggArray** available in KEGG: Kyoto Encyclopedia of Genes and Genomes website at <http://www.genome.jp/kegg/download/kegtools.html>. The results from analysis using KeggArray is not fully comparable with the DIA because the former only use the fold change as input while the DIA accounts for proportion of DEG compared to the genes present in array, the P-value of the change, and the fold change. In addition, the DIA does not weight more the down-regulated genes compared to the up-regulated, but the KeggArray tends to use the “limit enzyme” concept in the pathway, i.e., if in a enzymatic complex all genes coding for the proteins are up-regulated but one gene is down-regulated the object of the complex will appear green. An example is the Galactose Metabolism where the synthesis of lactose by the enzymatic complex Lactose Synthase (2.4.1.22) is formed by 3 proteins coded by *B4GALT2* (Gene ID 100125390) which was down-regulated (ratio expression at 60 vs. -30d = 0.71, P-value = 0.0002) and two strongly up-regulated genes, *B4GALT1* (Gene ID 281781, ratio expression at 60 vs. -30d = 1.70, P-value < 0.0001) and *LALBA* (Gene ID 281894, ratio expression at 60 vs. -30d = 75.4, P-value < 0.0001), but in the figure it appears green. The reader has to be aware that the images are shown to help in data interpretation but should be used with caution. The orange-red object denote up-regulation, the green down-regulation, the grey objects denote genes present (or annotated) in the bovine genome, and white objects denote genes not present (or not yet annotated) in the bovine genome.

Pathways are shown in order from the most to the least impacted as calculated by the DIA.

Contents

[METABOLIC PATHWAYS 10](#_Toc294790069)

[1.Metabolism; 11](#_Toc294790070)

[1.1 Carbohydrate Metabolism 11](#_Toc294790071)

[Amino sugar and nucleotide sugar metabolism 11](#_Toc294790072)

[Ascorbate and aldarate metabolism 12](#_Toc294790073)

[Butanoate Metabolism 13](#_Toc294790074)

[Citrate cycle (TCA) 14](#_Toc294790075)

[Fructose and Mannose Metabolism 15](#_Toc294790076)

[Galactose Metabolism 16](#_Toc294790077)

[Glycolysis / Gluconeogenesis 17](#_Toc294790078)

[Glyoxylate and dicarboxylate metabolism 18](#_Toc294790079)

[Inositol phosphate metabolism 19](#_Toc294790080)

[Pentose and glucuronate interconversions 20](#_Toc294790081)

[Pentose phosphate pathway 21](#_Toc294790082)

[Propanoate metabolism 22](#_Toc294790083)

[Pyruvate metabolism 23](#_Toc294790084)

[Starch and sucrose metabolism 24](#_Toc294790085)

[1.Metabolism 25](#_Toc294790086)

[1.2 Energy Metabolism 25](#_Toc294790087)

[Nitrogen metabolism 25](#_Toc294790088)

[Oxidative phosphorylation 26](#_Toc294790089)

[Sulfur metabolism 27](#_Toc294790090)

[1. Metabolism 28](#_Toc294790091)

[1.3 Lipid Metabolism 28](#_Toc294790092)

[alpha-Linolenic acid metabolism 28](#_Toc294790093)

[Arachidonic acid metabolism 29](#_Toc294790094)

[Biosynthesis of unsaturated fatty acids 30](#_Toc294790095)

[Ether lipid metabolism 31](#_Toc294790096)

[Fatty acid biosynthesis 32](#_Toc294790097)

[Fatty acid elongation in mitochondria 33](#_Toc294790098)

[Fatty acid metabolism 34](#_Toc294790099)

[Glycerolipid metabolism 35](#_Toc294790100)

[Glycerophospholipid metabolism 36](#_Toc294790101)

[Primary bile acid biosynthesis 37](#_Toc294790102)

[Sphingolipid metabolism 38](#_Toc294790103)

[Steroid biosynthesis 39](#_Toc294790104)

[Steroid hormone biosynthesis 40](#_Toc294790105)

[Synthesis and degradation of ketone bodies 41](#_Toc294790106)

[1. Metabolism 42](#_Toc294790107)

[1.4 Nucleotide Metabolism 42](#_Toc294790108)

[Purine metabolism 42](#_Toc294790109)

[Pyrimidine metabolism 43](#_Toc294790110)

[1. Metabolism 44](#_Toc294790111)

[1.5 Amino Acid Metabolism 44](#_Toc294790112)

[Alanine, aspartate and glutamate metabolism 44](#_Toc294790113)

[Arginine and proline metabolism 45](#_Toc294790114)

[Cysteine and methionine metabolism 46](#_Toc294790115)

[Glycine, serine and threonine metabolism 47](#_Toc294790116)

[Histidine metabolism 48](#_Toc294790117)

[Lysine degradation 49](#_Toc294790118)

[Phenylalanine metabolism 50](#_Toc294790119)

[Tryptophan metabolism 51](#_Toc294790120)

[Tyrosine metabolism 52](#_Toc294790121)

[Valine, leucine and isoleucine biosynthesis 53](#_Toc294790122)

[Valine, leucine and isoleucine degradation 54](#_Toc294790123)

[1. Metabolism 55](#_Toc294790124)

[1.6 Metabolism of Other Amino Acids 55](#_Toc294790125)

[beta-Alanine metabolism 55](#_Toc294790126)

[Cyanoamino acid metabolism 56](#_Toc294790127)

[Glutathione metabolism 57](#_Toc294790128)

[Taurine and hypotaurine metabolism 58](#_Toc294790129)

[1. Metabolism 59](#_Toc294790130)

[1.7 Glycan Biosynthesis and Metabolism 59](#_Toc294790131)

[Glycosaminoglycan biosynthesis - chondroitin sulfate 59](#_Toc294790132)

[Glycosaminoglycan biosynthesis - keratan sulfate 60](#_Toc294790133)

[Glycosaminoglycan degradation 61](#_Toc294790134)

[Glycosphingolipid biosynthesis - ganglio series 62](#_Toc294790135)

[Glycosphingolipid biosynthesis - globo series 63](#_Toc294790136)

[Glycosphingolipid biosynthesis - lacto and neolacto series 64](#_Toc294790137)

[Glycosylphosphatidylinositol(GPI)-anchor biosynthesis 65](#_Toc294790138)

[N-Glycan biosynthesis 66](#_Toc294790139)

[O-Glycan biosynthesis 67](#_Toc294790140)

[Other glycan degradation 68](#_Toc294790141)

[1. Metabolism 69](#_Toc294790142)

[1.8 Metabolism of Cofactors and Vitamins 69](#_Toc294790143)

[Biotin metabolism 69](#_Toc294790144)

[Folate biosynthesis 70](#_Toc294790145)

[Nicotinate and nicotinamide metabolism 71](#_Toc294790146)

[One carbon pool by folate 72](#_Toc294790147)

[Pantothenate and CoA biosynthesis 73](#_Toc294790148)

[Porphyrin and chlorophyll metabolism 74](#_Toc294790149)

[Retinol metabolism 75](#_Toc294790150)

[Riboflavin metabolism 76](#_Toc294790151)

[Thiamine metabolism 77](#_Toc294790152)

[Vitamin B6 metabolism 78](#_Toc294790153)

[1. Metabolism 79](#_Toc294790154)

[1.9 Metabolism of Terpenoids and Polyketides 79](#_Toc294790155)

[Terpenoid backbone biosynthesis 79](#_Toc294790156)

[1. Metabolism 80](#_Toc294790157)

[1.10 Biosynthesis of Other Secondary Metabolites 80](#_Toc294790158)

[Caffeine metabolism 80](#_Toc294790159)

[1. Metabolism 81](#_Toc294790160)

[1.11 Xenobiotics Biodegradation and Metabolism 81](#_Toc294790161)

[Drug metabolism - cytochrome P450 81](#_Toc294790162)

[Drug metabolism - other enzymes 82](#_Toc294790163)

[Metabolism of xenobiotics by cytochrome P450 83](#_Toc294790164)

[2. Genetic Information Processing 84](#_Toc294790165)

[2.1 Transcription 84](#_Toc294790166)

[Basal transcription factors 84](#_Toc294790167)

[RNA polymerase 85](#_Toc294790168)

[Spliceosome 86](#_Toc294790169)

[2. Genetic Information Processing 87](#_Toc294790170)

[2.2 Translation 87](#_Toc294790171)

[Aminoacyl-tRNA biosynthesis 87](#_Toc294790172)

[mRNA surveillance pathway 88](#_Toc294790173)

[Ribosome 89](#_Toc294790174)

[Ribosome biogenesis in eukaryotes 90](#_Toc294790175)

[RNA transport 91](#_Toc294790176)

[2. Genetic Information Processing 92](#_Toc294790177)

[2.3 Folding, Sorting and Degradation 92](#_Toc294790178)

[Proteasome 92](#_Toc294790179)

[Protein export 93](#_Toc294790180)

[Protein processing in endoplasmic reticulum 94](#_Toc294790181)

[RNA degradation 95](#_Toc294790182)

[SNARE interactions in vesicular transport 96](#_Toc294790183)

[Sulfur relay system 97](#_Toc294790184)

[Ubiquitin mediated proteolysis 98](#_Toc294790185)

[2. Genetic Information Processing 99](#_Toc294790186)

[2.4 Replication and Repair 99](#_Toc294790187)

[Base excision repair 99](#_Toc294790188)

[DNA replication 100](#_Toc294790189)

[Homologous recombination 101](#_Toc294790190)

[Mismatch repair 102](#_Toc294790191)

[Non-homologous end-joining 103](#_Toc294790192)

[Nucleotide excision repair 104](#_Toc294790193)

[3. Environmental Information Processing 105](#_Toc294790194)

[3.1 Membrane Transport 105](#_Toc294790195)

[ABC transporters 105](#_Toc294790196)

[3. Environmental Information Processing 106](#_Toc294790197)

[3.2 Signal Transduction 106](#_Toc294790198)

[Calcium signaling pathway 106](#_Toc294790199)

[ErbB signaling pathway 107](#_Toc294790200)

[Hedgehog signaling pathway 108](#_Toc294790201)

[Jak-STAT signaling pathway 109](#_Toc294790202)

[MAPK signaling pathway 110](#_Toc294790203)

[mTOR signaling pathway 111](#_Toc294790204)

[Notch signaling pathway 112](#_Toc294790205)

[Phosphatidylinositol signaling system 113](#_Toc294790206)

[TGF-beta signaling pathway 114](#_Toc294790207)

[VEGF signaling pathway 115](#_Toc294790208)

[Wnt signaling pathway 116](#_Toc294790209)

[3. Environmental Information Processing 117](#_Toc294790210)

[3.3 Signaling Molecules and Interaction 117](#_Toc294790211)

[Cell adhesion molecules (CAMs) 117](#_Toc294790212)

[Cytokine-cytokine receptor interaction 118](#_Toc294790213)

[ECM-receptor interaction 119](#_Toc294790214)

[4. Cellular Processes 120](#_Toc294790215)

[4.1 Transport and Catabolism 120](#_Toc294790216)

[Endocytosis 120](#_Toc294790217)

[Lysosome 121](#_Toc294790218)

[Peroxisome 122](#_Toc294790219)

[Phagosome 123](#_Toc294790220)

[Regulation of autophagy 124](#_Toc294790221)

[4. Cellular Processes 125](#_Toc294790222)

[4.2 Cell Motility 125](#_Toc294790223)

[Regulation of actin cytoskeleton 125](#_Toc294790224)

[4. Cellular Processes 126](#_Toc294790225)

[4.3 Cell Growth and Death 126](#_Toc294790226)

[Apoptosis 126](#_Toc294790227)

[Cell cycle 127](#_Toc294790228)

[Oocyte meiosis 128](#_Toc294790229)

[p53 signaling pathway 129](#_Toc294790230)

[4. Cellular Processes 130](#_Toc294790231)

[4.4 Cell Communication 130](#_Toc294790232)

[Adherens junction 130](#_Toc294790233)

[Focal adhesion 131](#_Toc294790234)

[Gap junction 132](#_Toc294790235)

[Tight junction 133](#_Toc294790236)

[5. Organismal Systems 134](#_Toc294790237)

[5.1 Immune System 134](#_Toc294790238)

[Antigen processing and presentation 134](#_Toc294790239)

[B cell receptor signaling pathway 135](#_Toc294790240)

[Chemokine signaling pathway 136](#_Toc294790241)

[Complement and coagulation cascades 137](#_Toc294790242)

[Cytosolic DNA-sensing pathway 138](#_Toc294790243)

[Fc epsilon RI signaling pathway 139](#_Toc294790244)

[Fc gamma R-mediated phagocytosis 140](#_Toc294790245)

[Hematopoietic cell lineage 141](#_Toc294790246)

[Intestinal immune network for IgA production 142](#_Toc294790247)

[Leukocyte transendothelial migration 143](#_Toc294790248)

[Natural killer cell mediated cytotoxicity 144](#_Toc294790249)

[NOD-like receptor signaling pathway 145](#_Toc294790250)

[RIG-I-like receptor signaling pathway 146](#_Toc294790251)

[T cell receptor signaling pathway 147](#_Toc294790252)

[Toll-like receptor signaling pathway 148](#_Toc294790253)

[5. Organismal Systems 149](#_Toc294790254)

[5.2 Endocrine System 149](#_Toc294790255)

[Adipocytokine signaling pathway 149](#_Toc294790256)

[GnRH signaling pathway 150](#_Toc294790257)

[Insulin signaling pathway 151](#_Toc294790258)

[Melanogenesis 152](#_Toc294790259)

[PPAR signaling pathway 153](#_Toc294790260)

[Progesterone-mediated oocyte maturation 154](#_Toc294790261)

[Renin-angiotensin system 155](#_Toc294790262)

[5. Organismal Systems 156](#_Toc294790263)

[5.3 Circulatory System 156](#_Toc294790264)

[Cardiac muscle contraction 156](#_Toc294790265)

[Vascular smooth muscle contraction 157](#_Toc294790266)

[5. Organismal Systems 158](#_Toc294790267)

[5.4 Digestive System 158](#_Toc294790268)

[Bile secretion 158](#_Toc294790269)

[Carbohydrate digestion and absorption 159](#_Toc294790270)

[Fat digestion and absorption 160](#_Toc294790271)

[Gastric acid secretion 161](#_Toc294790272)

[Mineral absorption 162](#_Toc294790273)

[Pancreatic secretion 163](#_Toc294790274)

[Protein digestion and absorption 164](#_Toc294790275)

[Salivary secretion 165](#_Toc294790276)

[Vitamin digestion and absorption 166](#_Toc294790277)

[5. Organismal Systems 167](#_Toc294790278)

[5.5 Excretory System 167](#_Toc294790279)

[Aldosterone-regulated sodium reabsorption 167](#_Toc294790280)

[Collecting duct acid secretion 168](#_Toc294790281)

[Endocrine and other factor-regulated calcium reabsorption 169](#_Toc294790282)

[Proximal tubule bicarbonate reclamation 170](#_Toc294790283)

[Vasopressin-regulated water reabsorption 171](#_Toc294790284)

[5. Organismal Systems 172](#_Toc294790285)

[5.6 Nervous System 172](#_Toc294790286)

[Glutamatergic synapse 172](#_Toc294790287)

[Long-term depression 173](#_Toc294790288)

[Long-term potentiation 174](#_Toc294790289)

[Neurotrophin signaling pathway 175](#_Toc294790290)

[5. Organismal Systems 176](#_Toc294790291)

[5.7 Sensory System 176](#_Toc294790292)

[Phototransduction 176](#_Toc294790293)

[5. Organismal Systems 177](#_Toc294790294)

[5.8 Development 177](#_Toc294790295)

[Axon guidance 177](#_Toc294790296)

[Dorso-ventral axis formation 178](#_Toc294790297)

[Osteoclast differentiation 179](#_Toc294790298)

[5. Organismal Systems 180](#_Toc294790299)

[5.9 Environmental Adaptation 180](#_Toc294790300)

[Circadian rhythm - mammal 180](#_Toc294790301)

# METABOLIC PATHWAYS

Orange line denote positive or increased flux and light green lines decreased flux. Other color lines denote the overall category of pathways
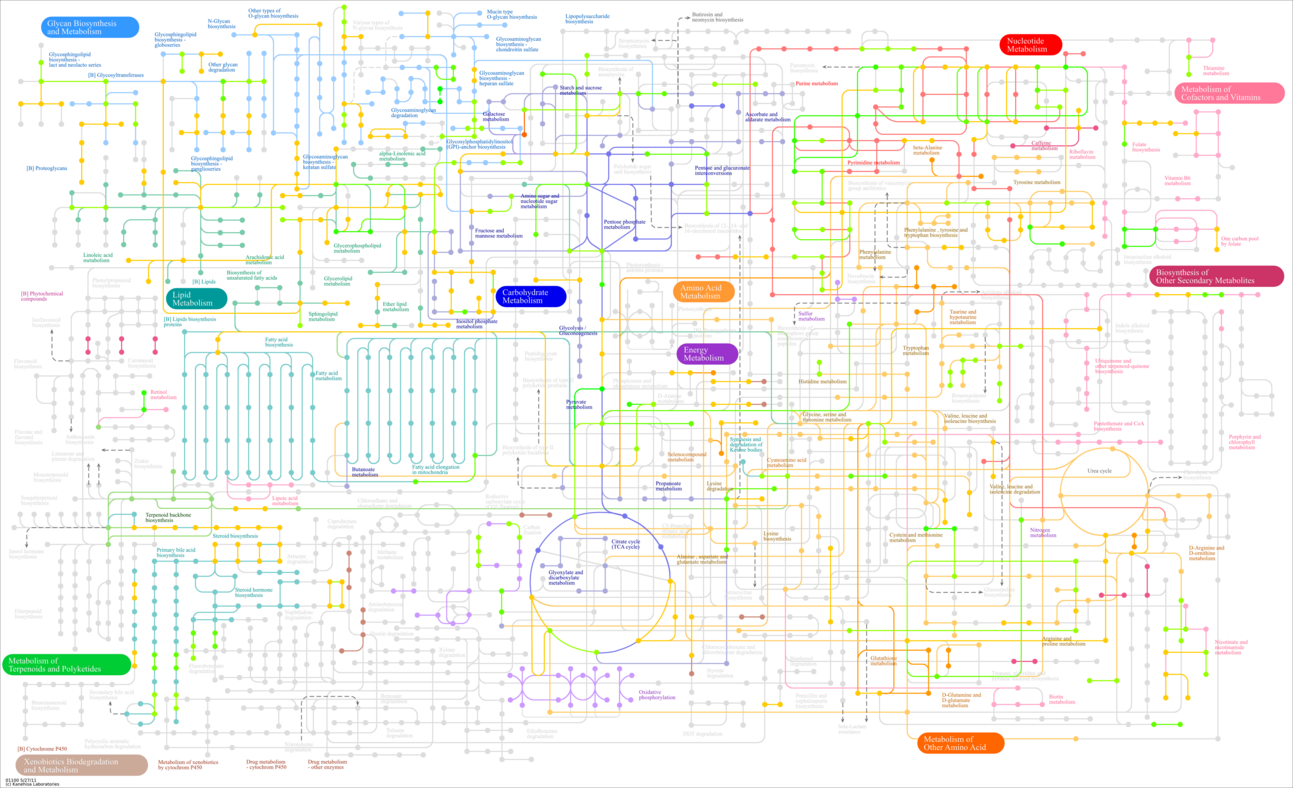


# 1.Metabolism;

## 1.1 Carbohydrate Metabolism

### Amino sugar and nucleotide sugar metabolism


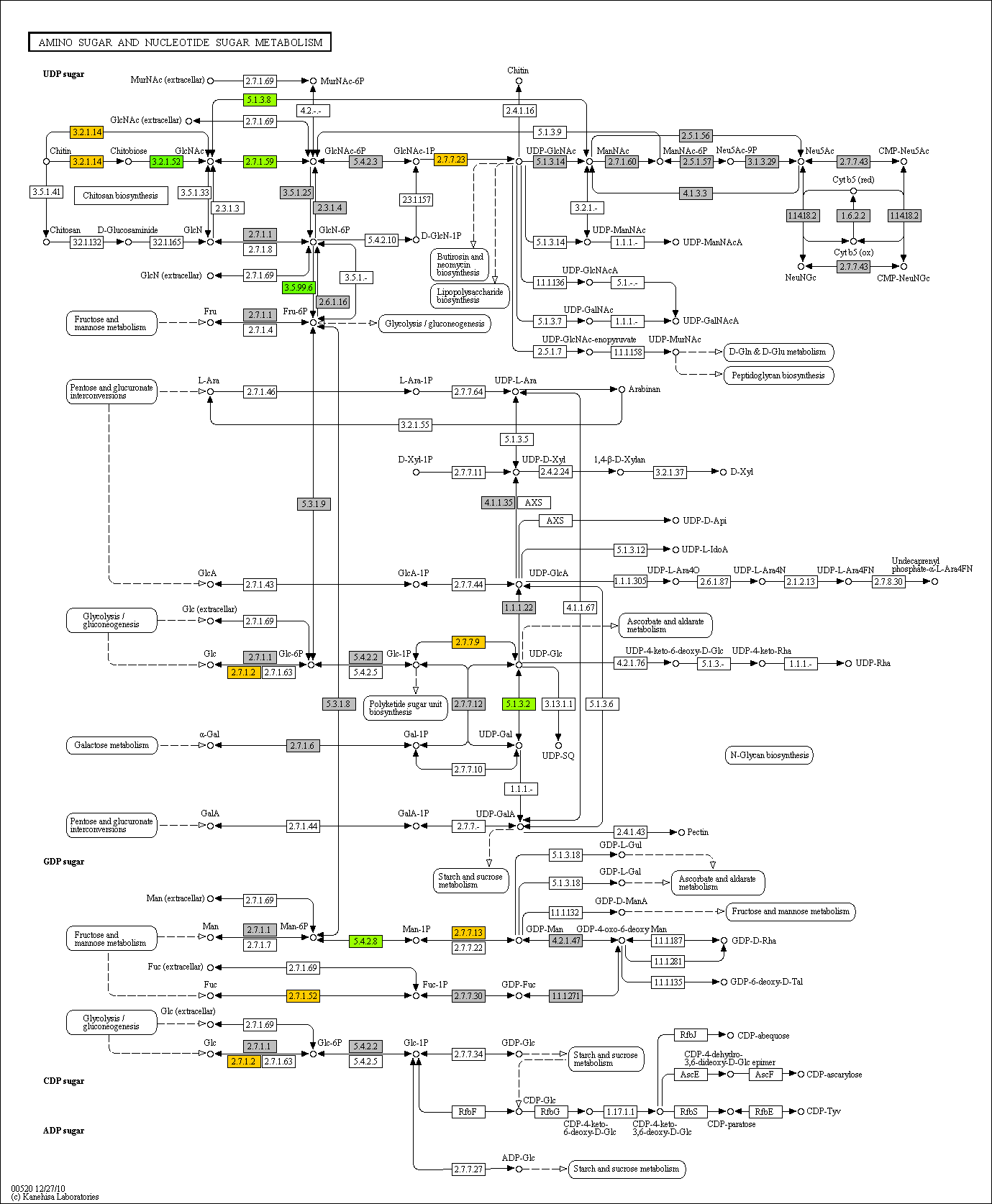


### Ascorbate and aldarate metabolism


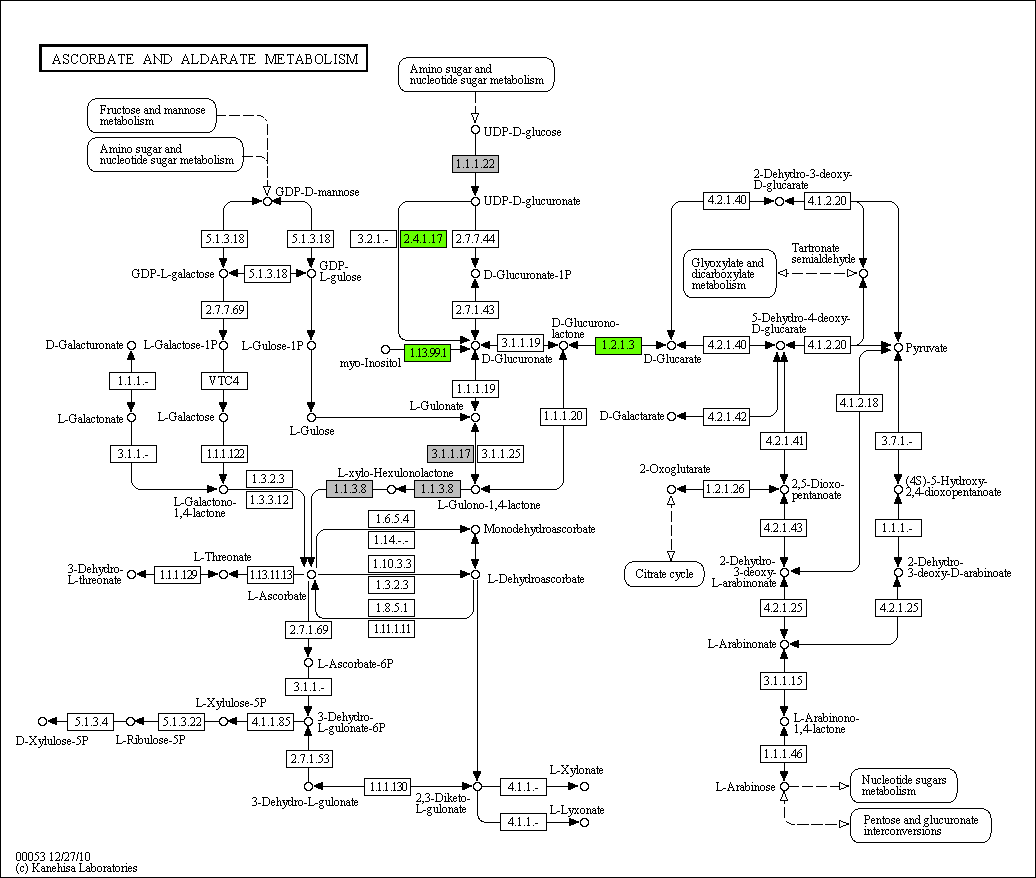


### Butanoate Metabolism


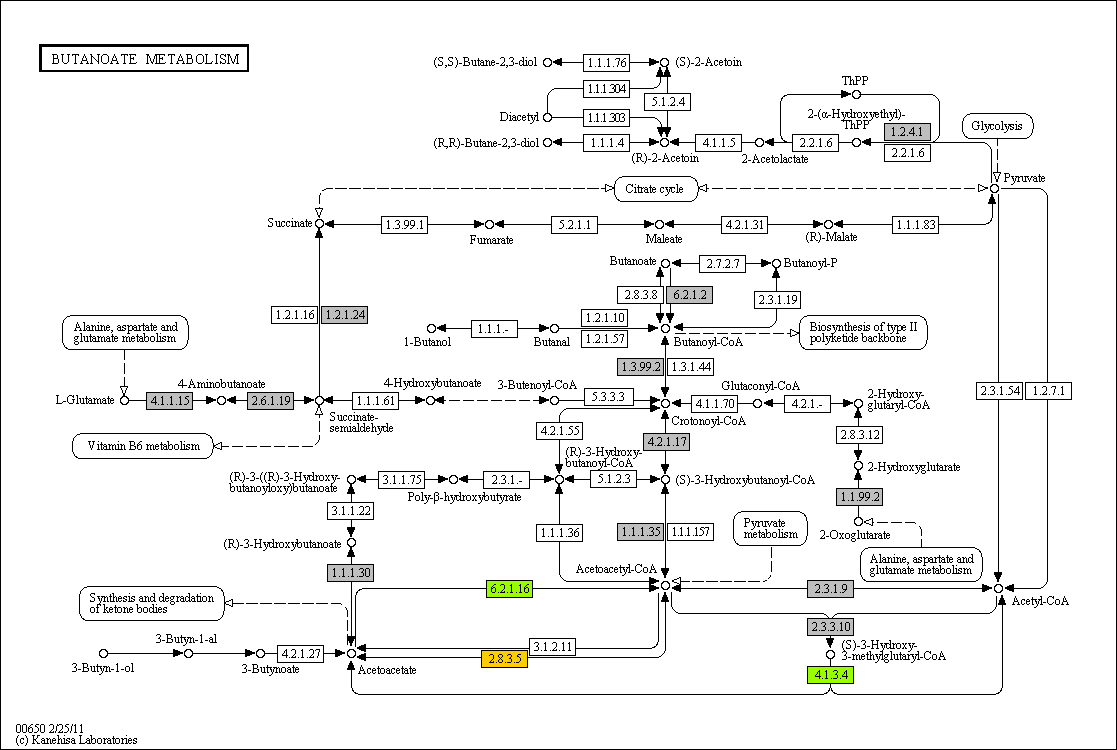


### Citrate cycle (TCA)


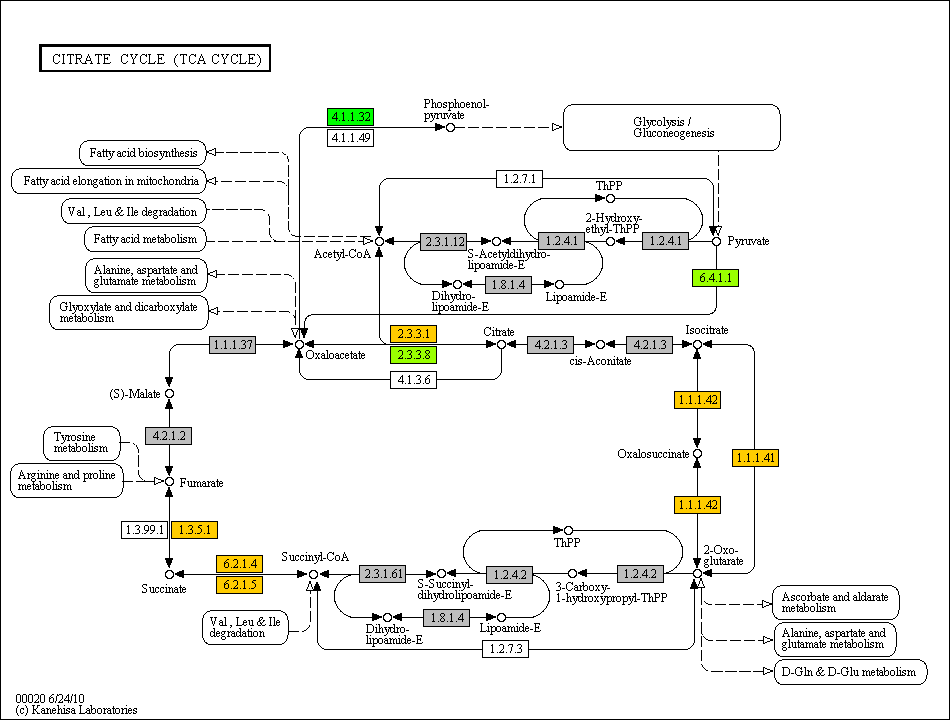


### Fructose and Mannose Metabolism


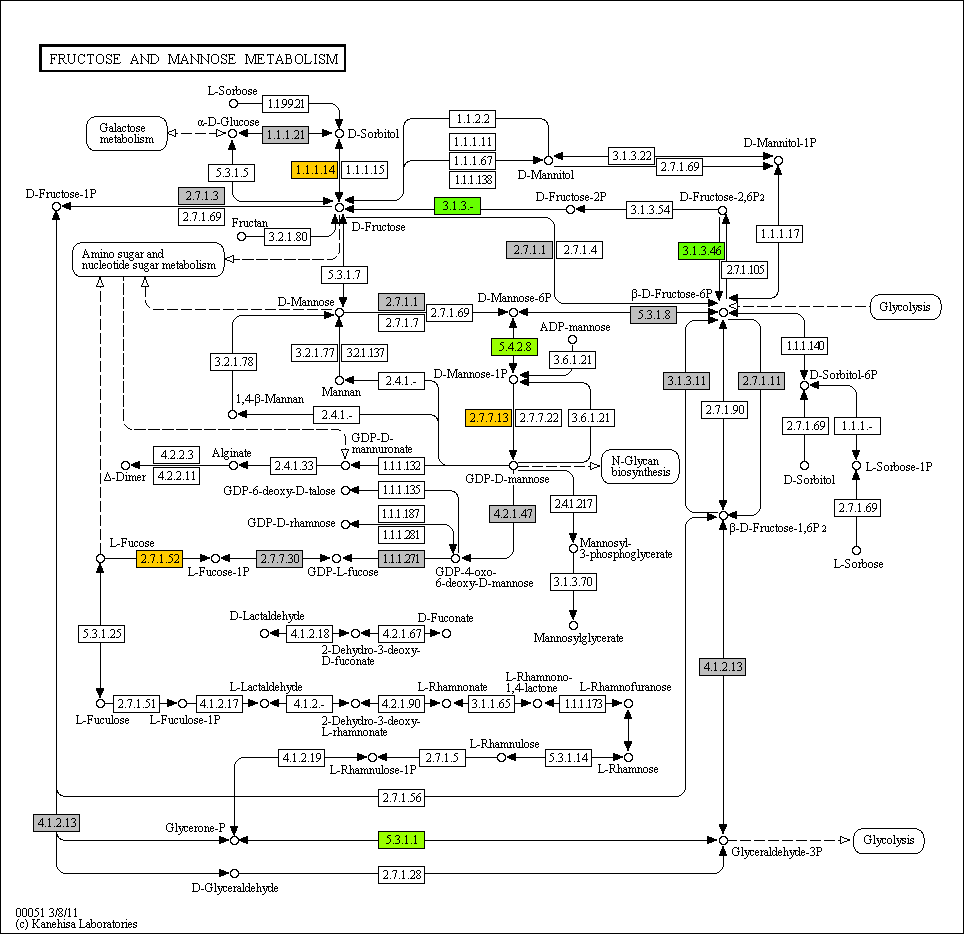


Galactose Metabolism
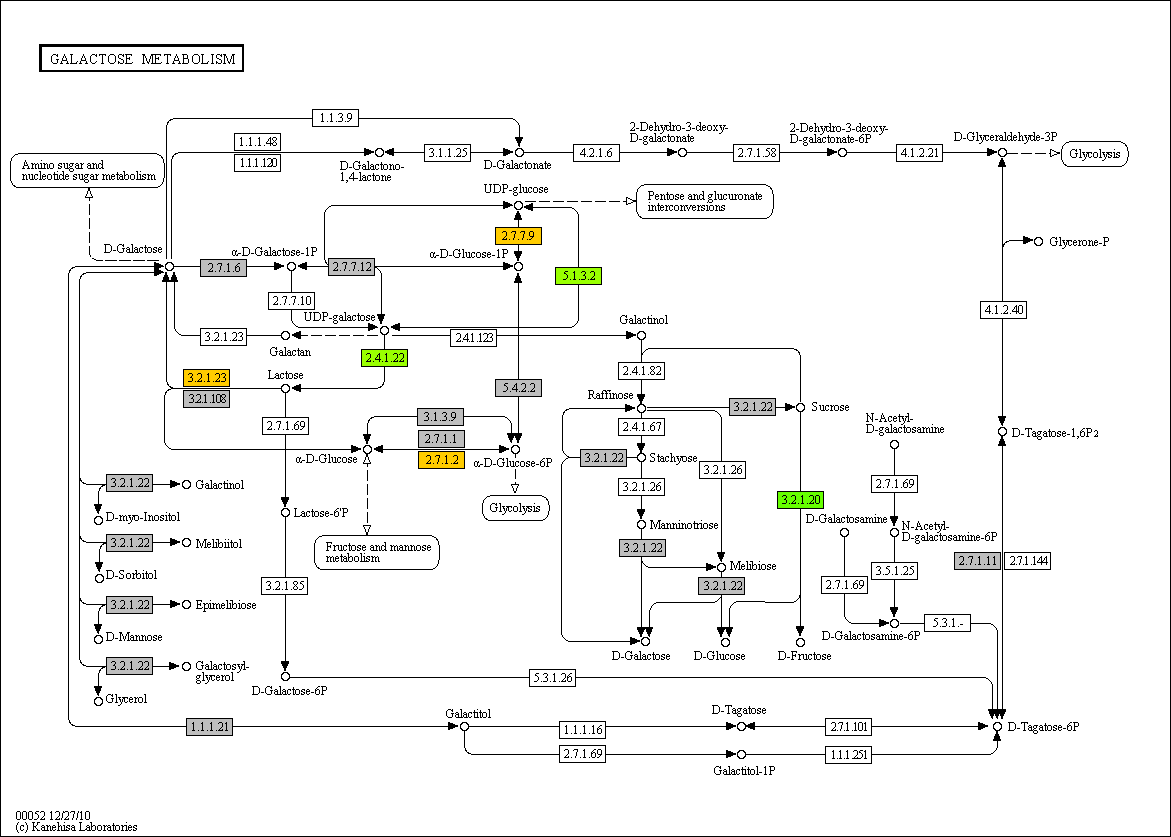


Glycolysis / Gluconeogenesis
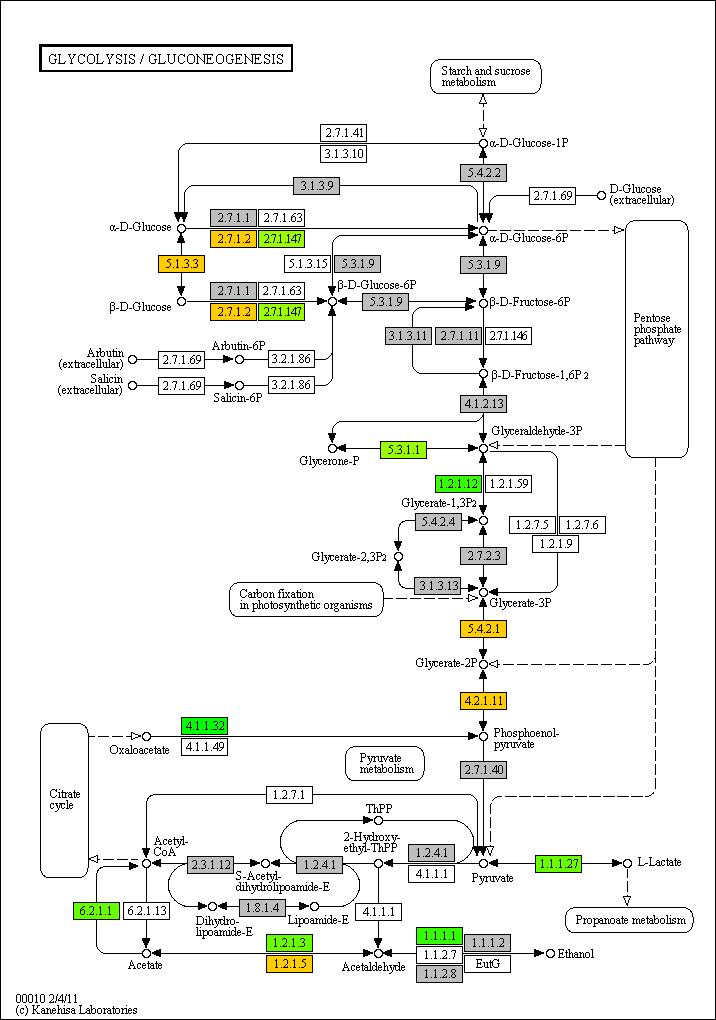


### Glyoxylate and dicarboxylate metabolism


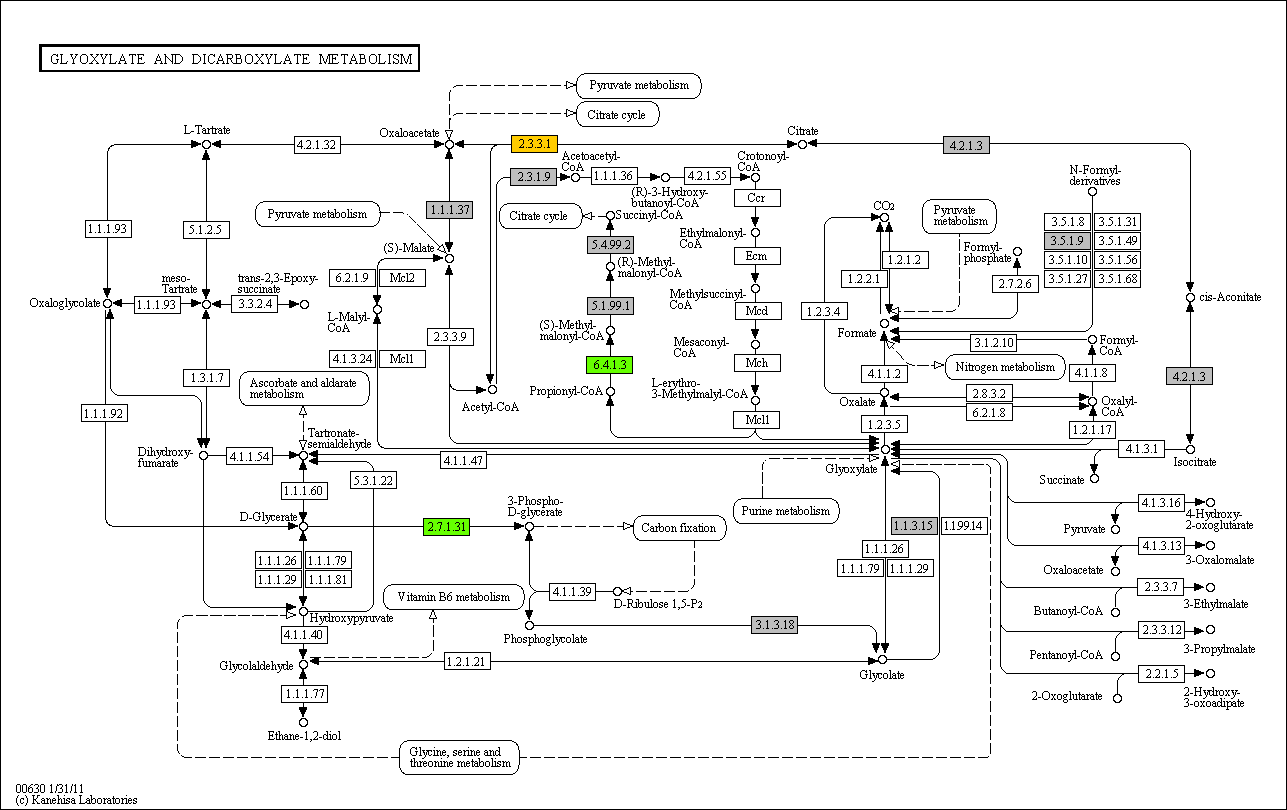


### Inositol phosphate metabolism


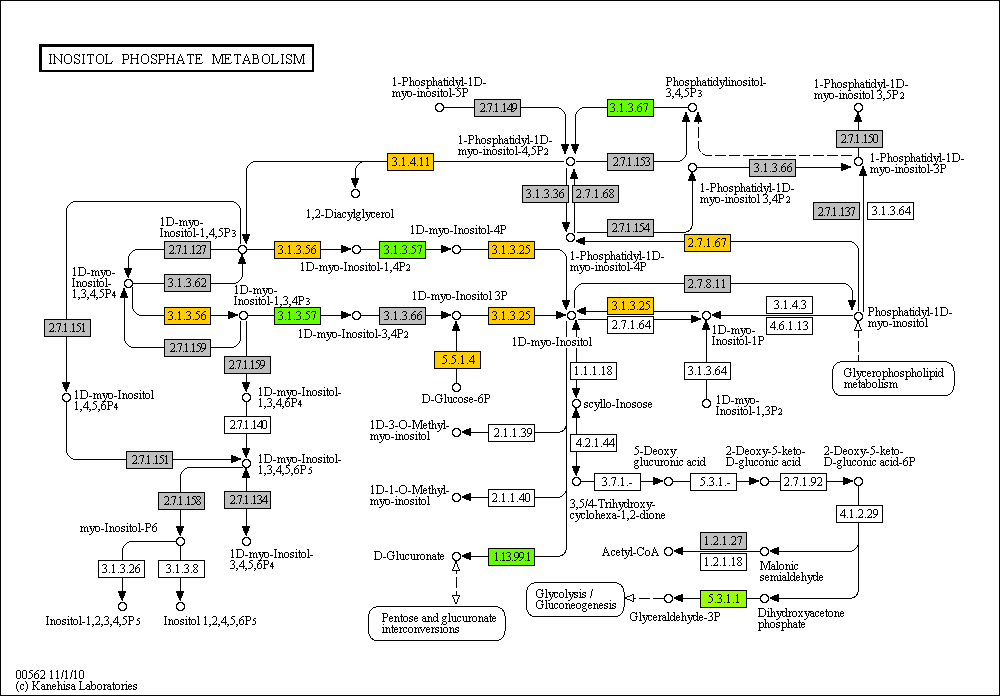


### Pentose and glucuronate interconversions


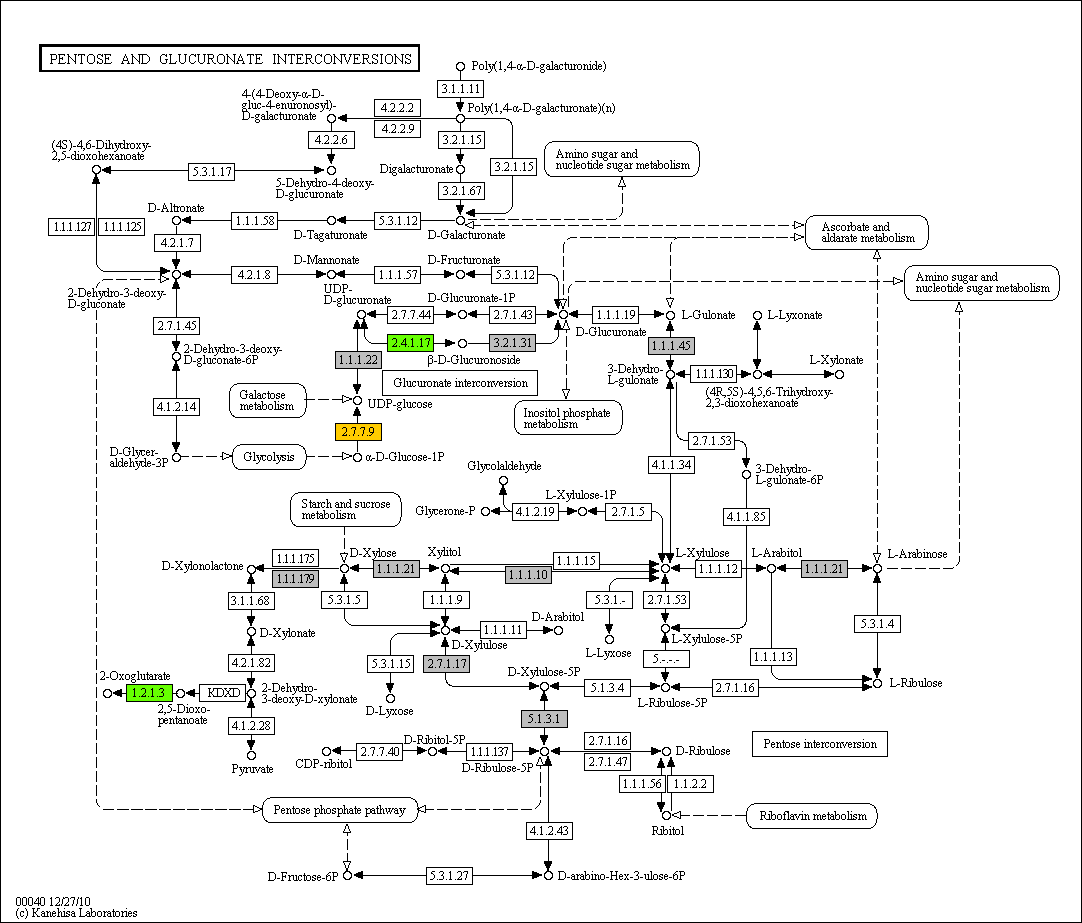


### Pentose phosphate pathway


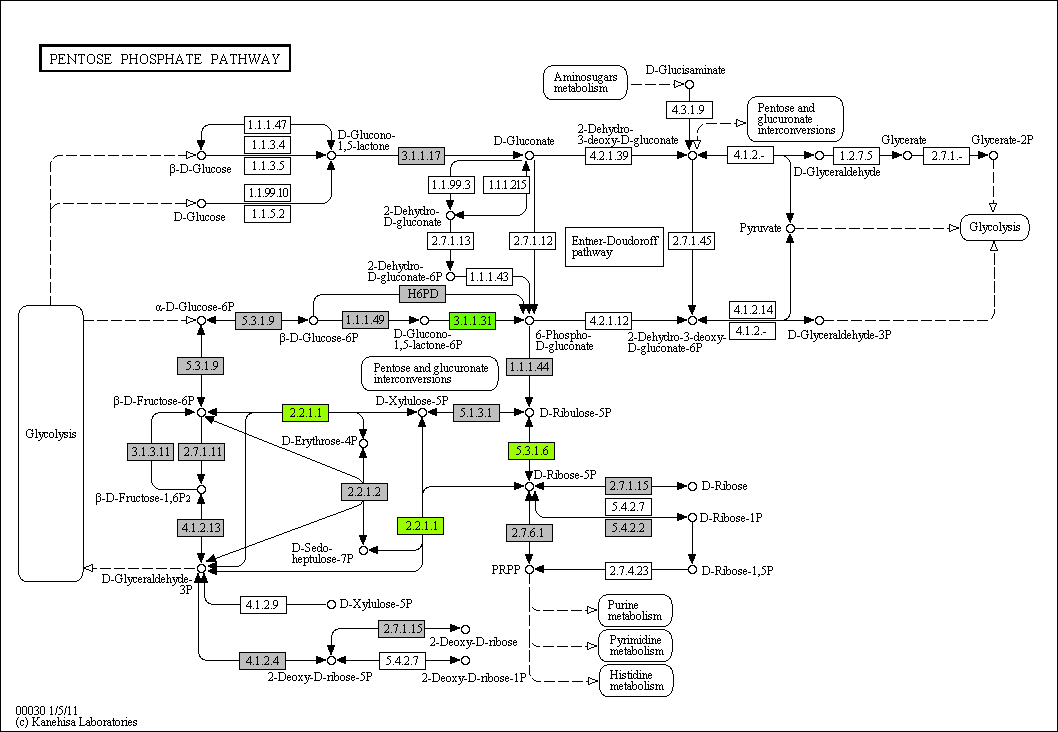


### Propanoate metabolism


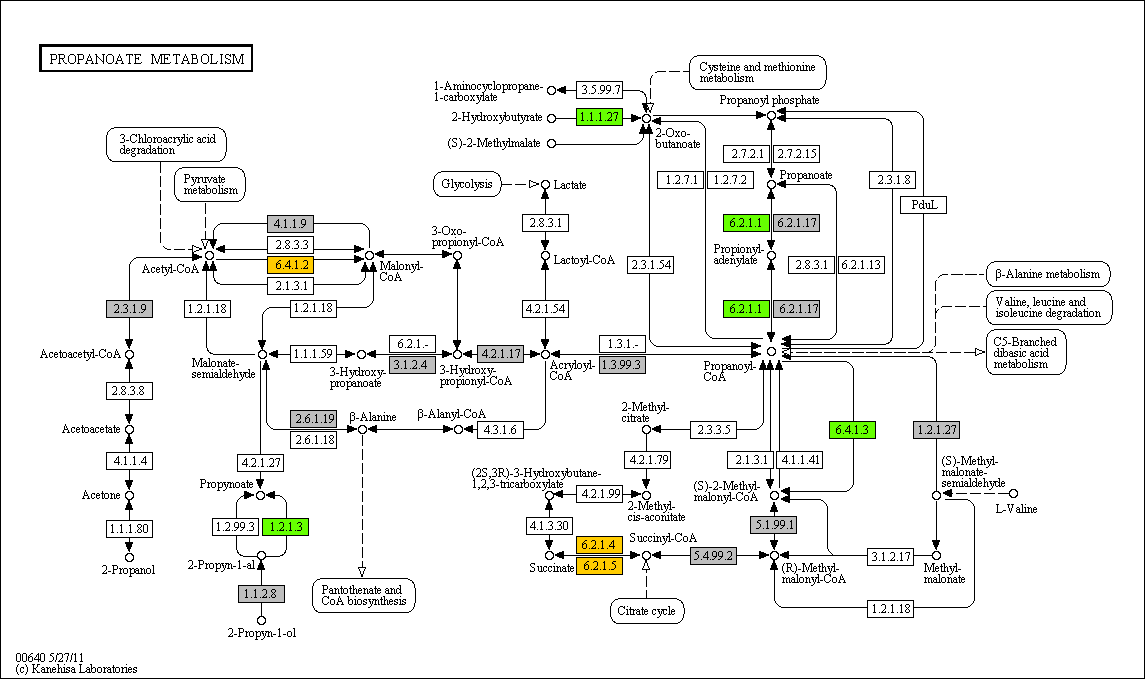


### Pyruvate metabolism


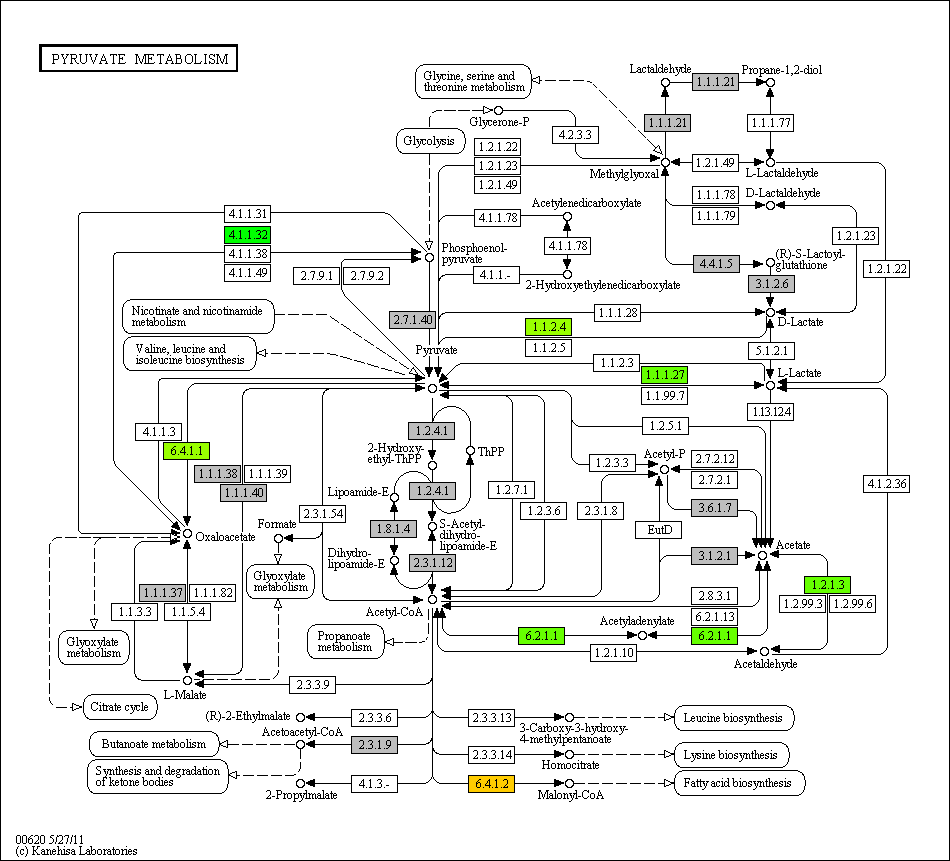


### Starch and sucrose metabolism


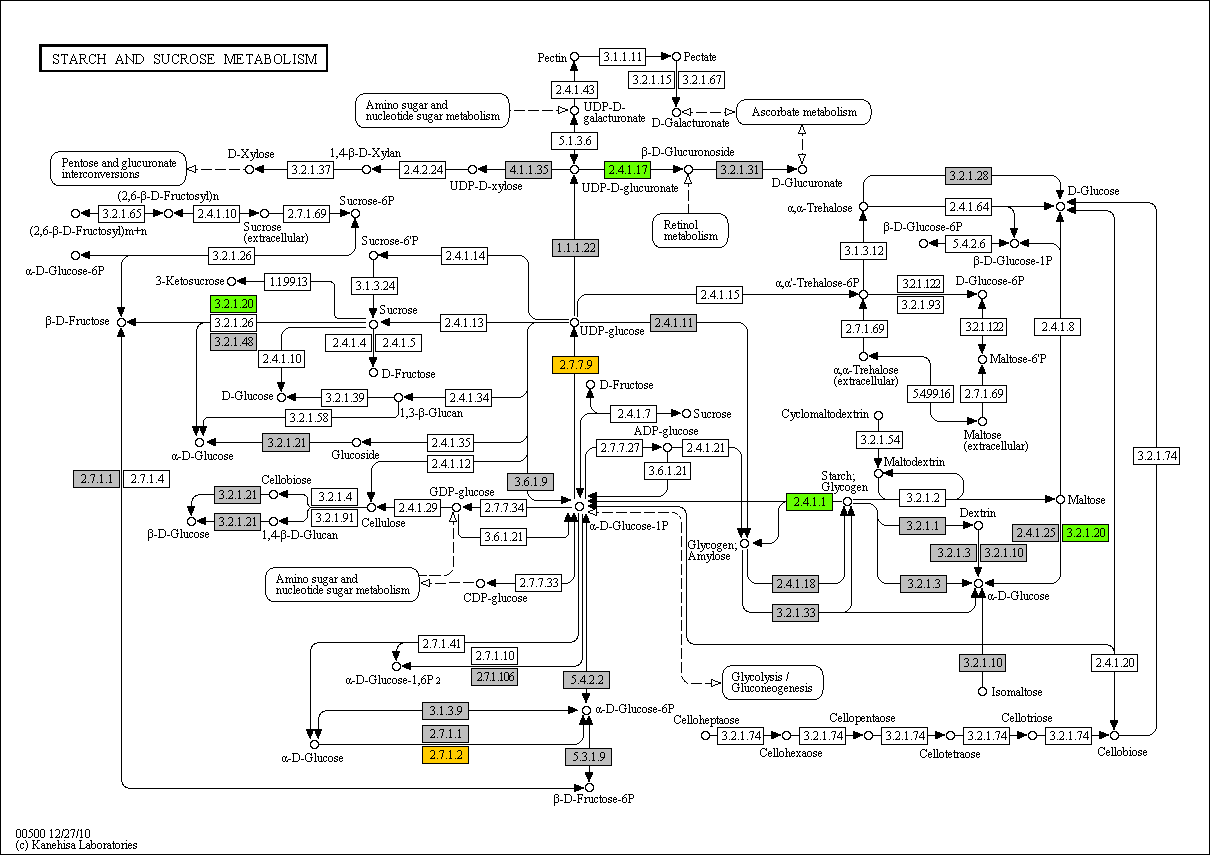


# 1.Metabolism

## 1.2 Energy Metabolism

### Nitrogen metabolism


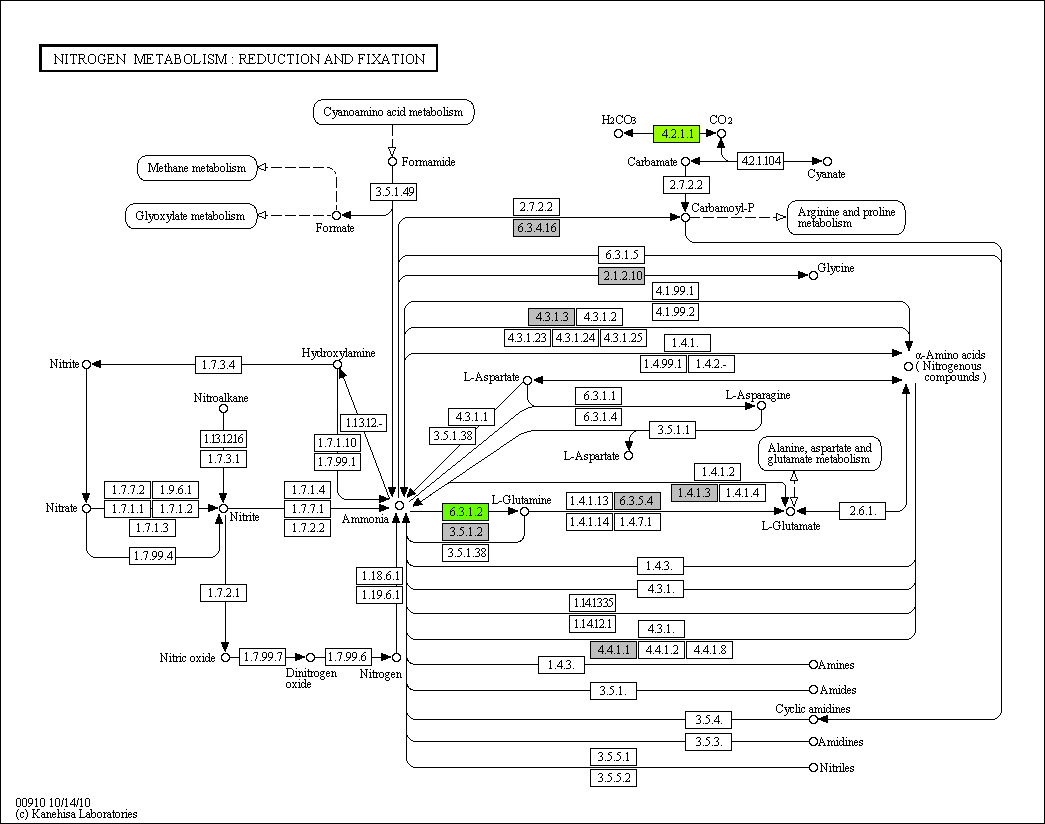


### Oxidative phosphorylation


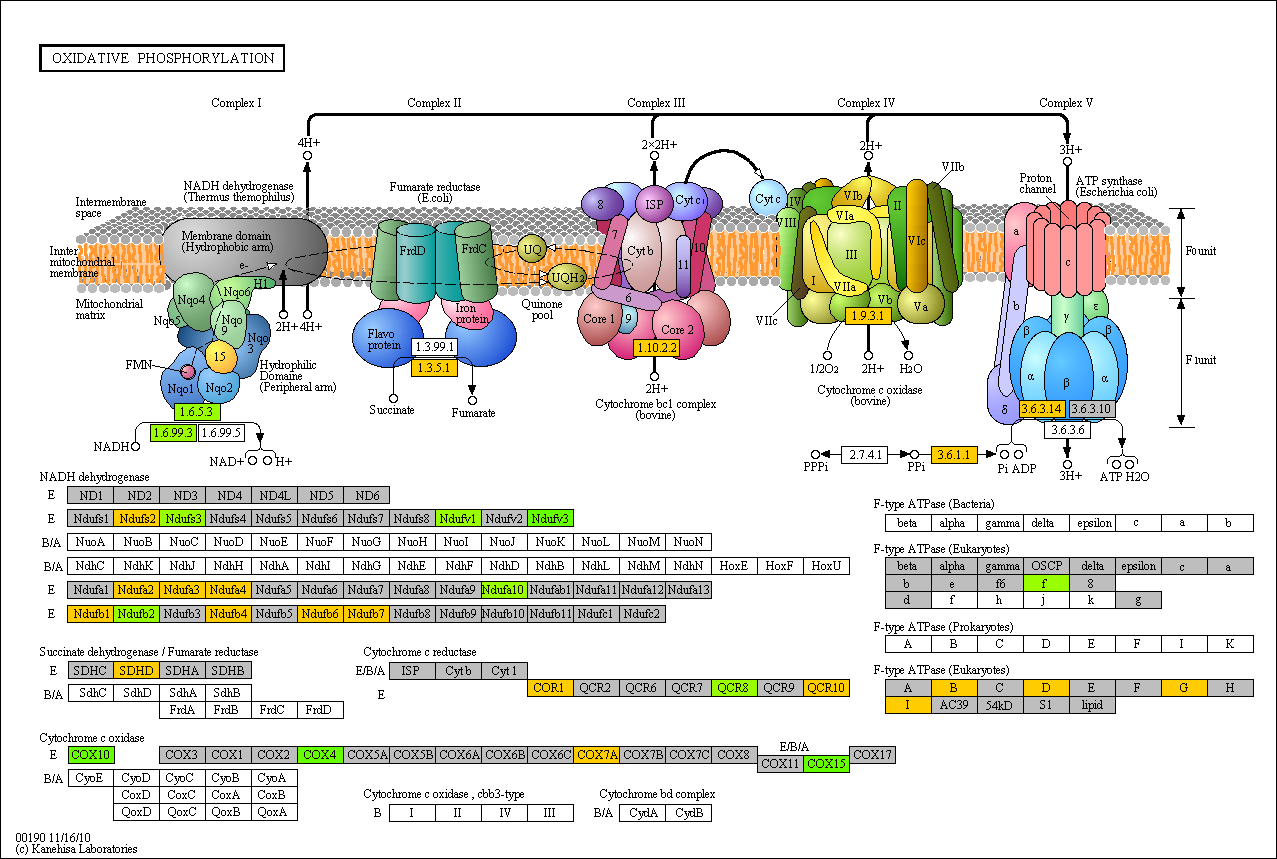


### Sulfur metabolism


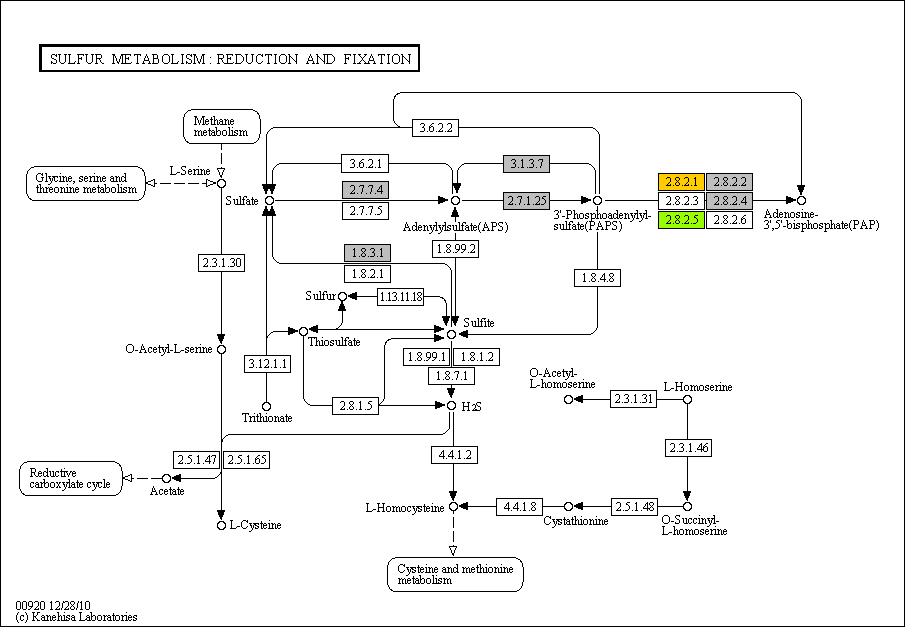


# 1. Metabolism

## 1.3 Lipid Metabolism

### alpha-Linolenic acid metabolism


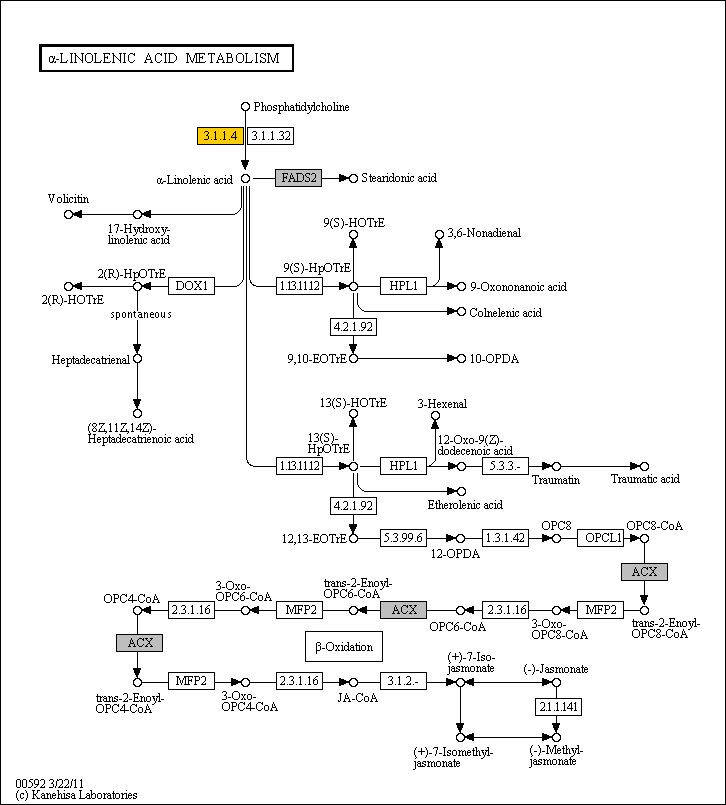


### Arachidonic acid metabolism


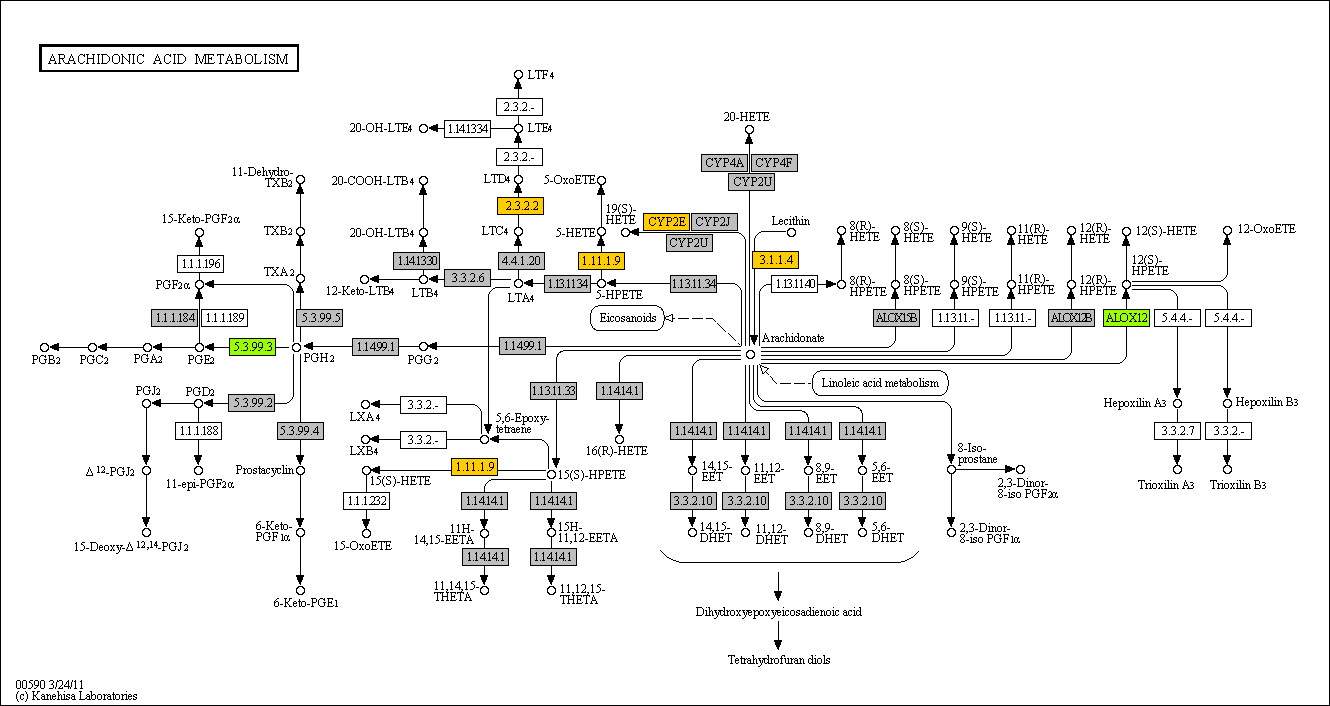


### Biosynthesis of unsaturated fatty acids


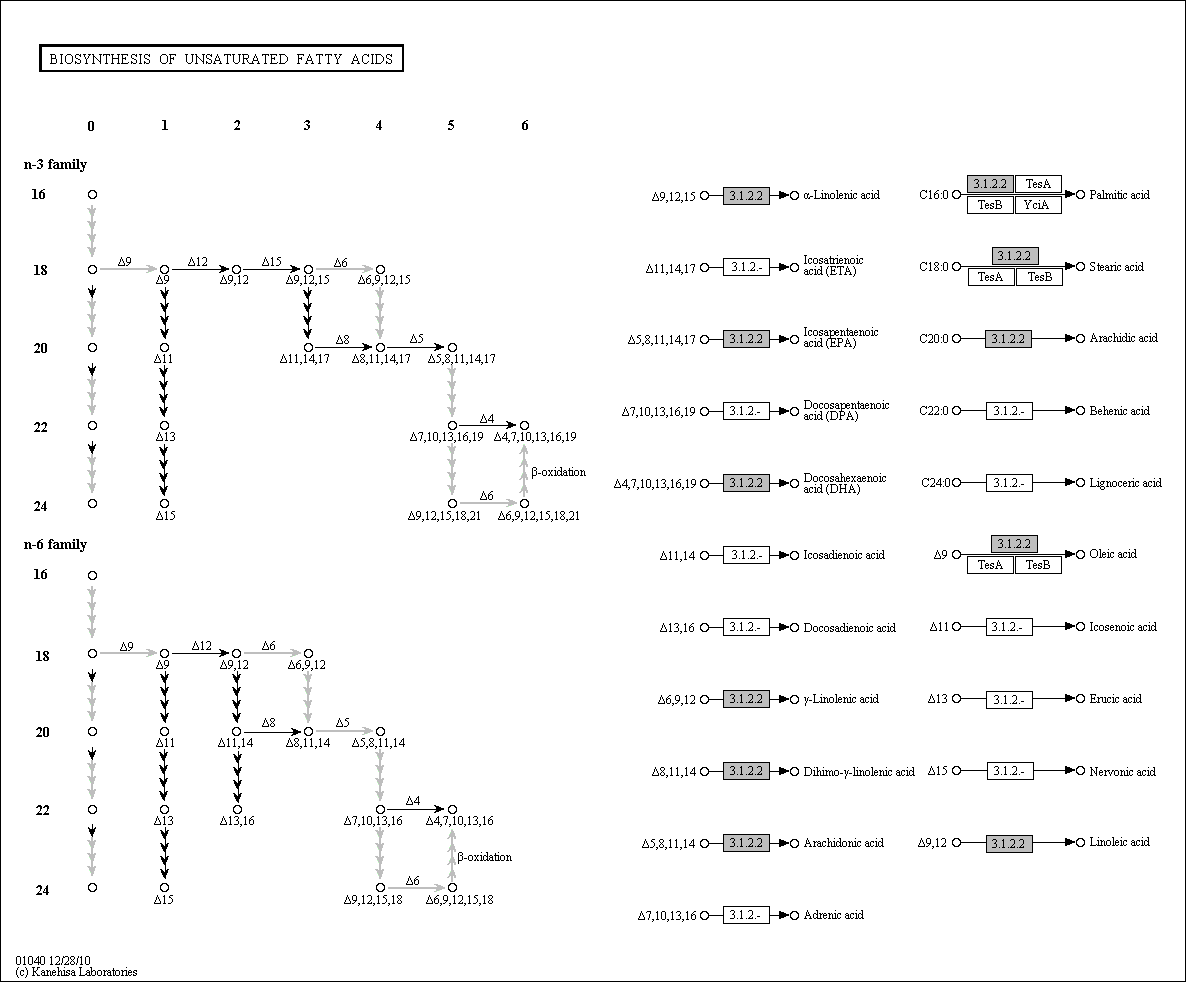


### Ether lipid metabolism


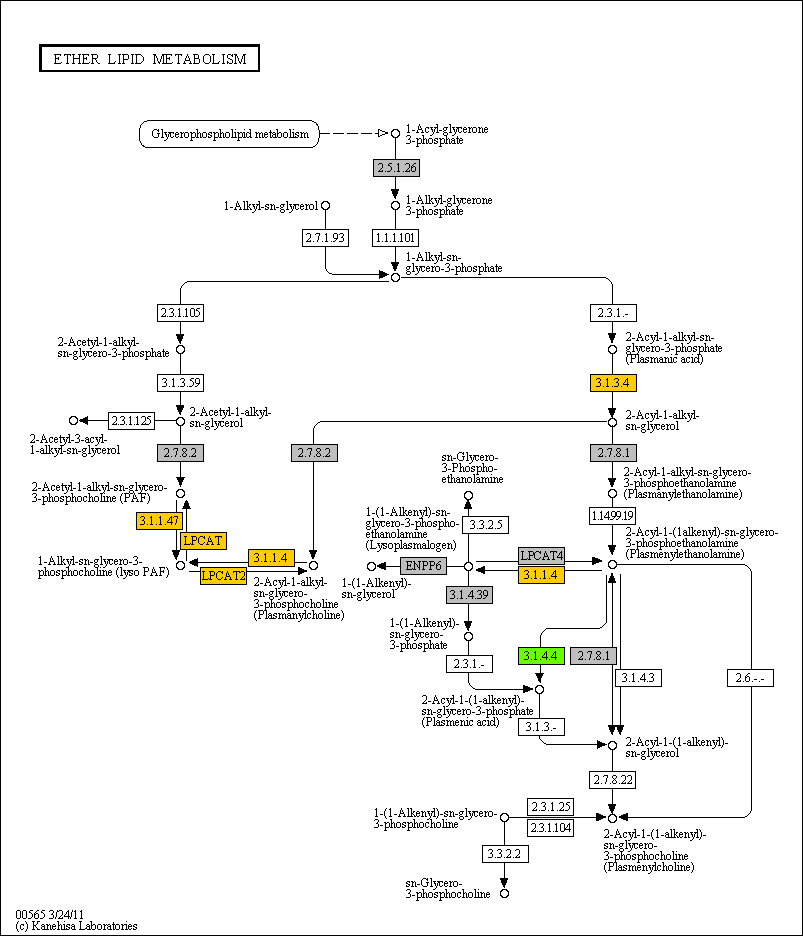


### Fatty acid biosynthesis


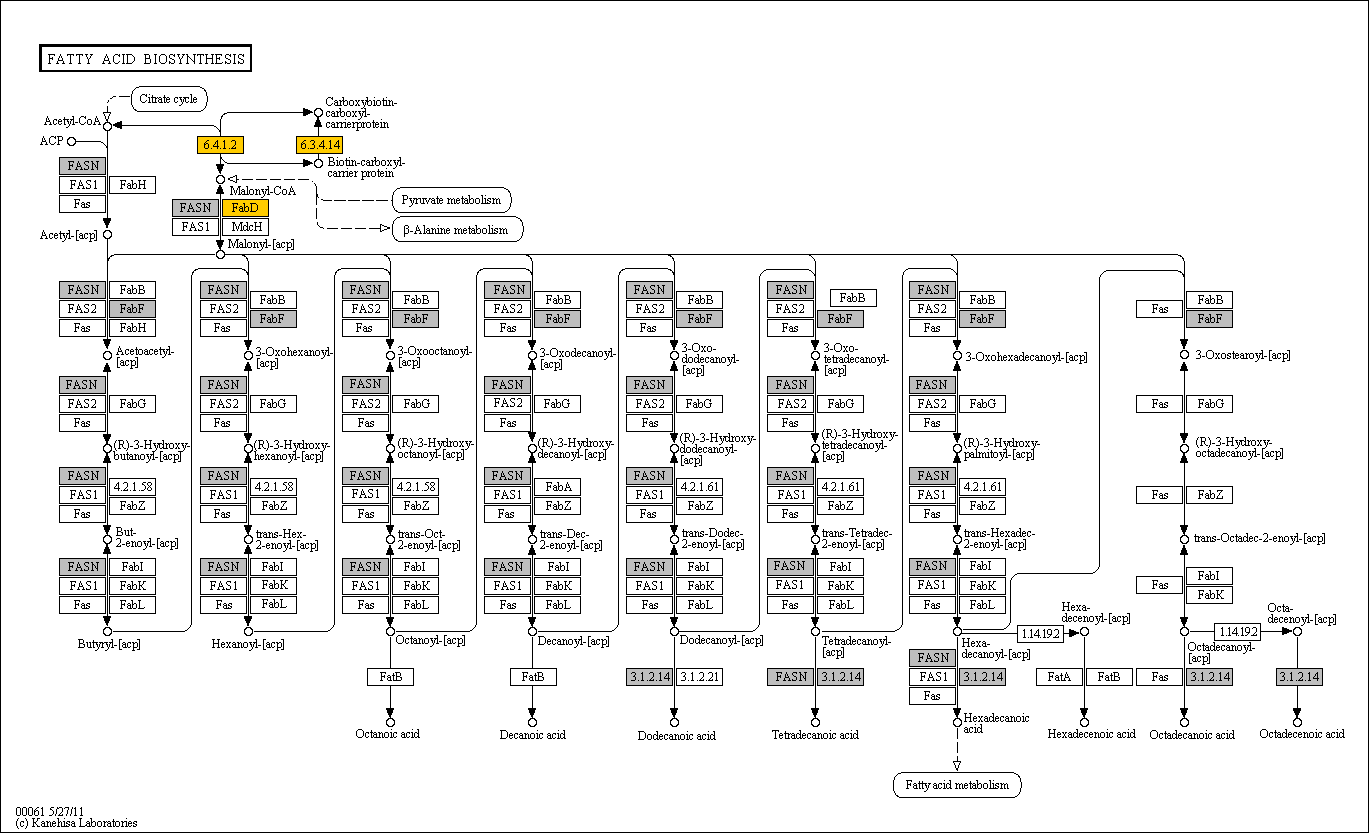


### Fatty acid elongation in mitochondria


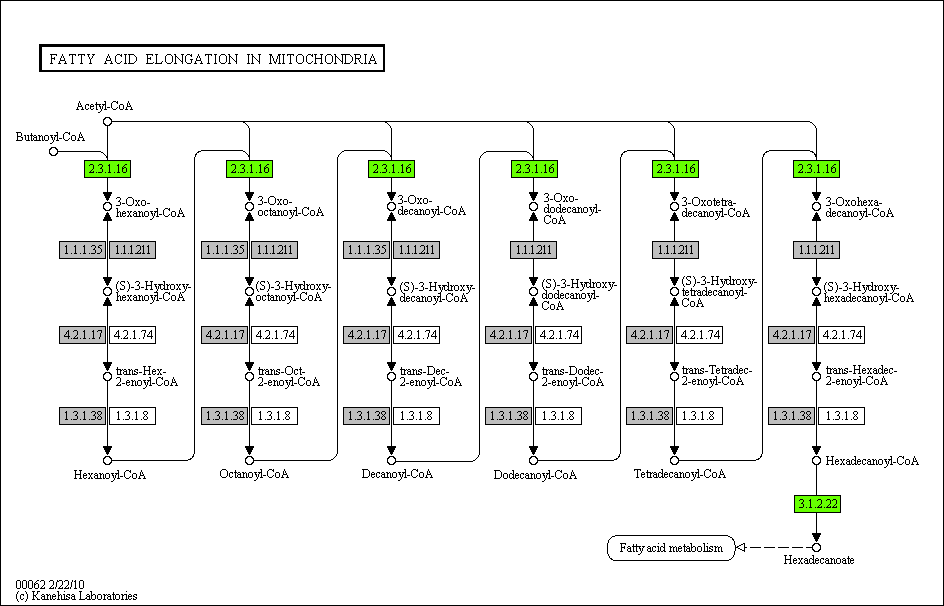


### Fatty acid metabolism


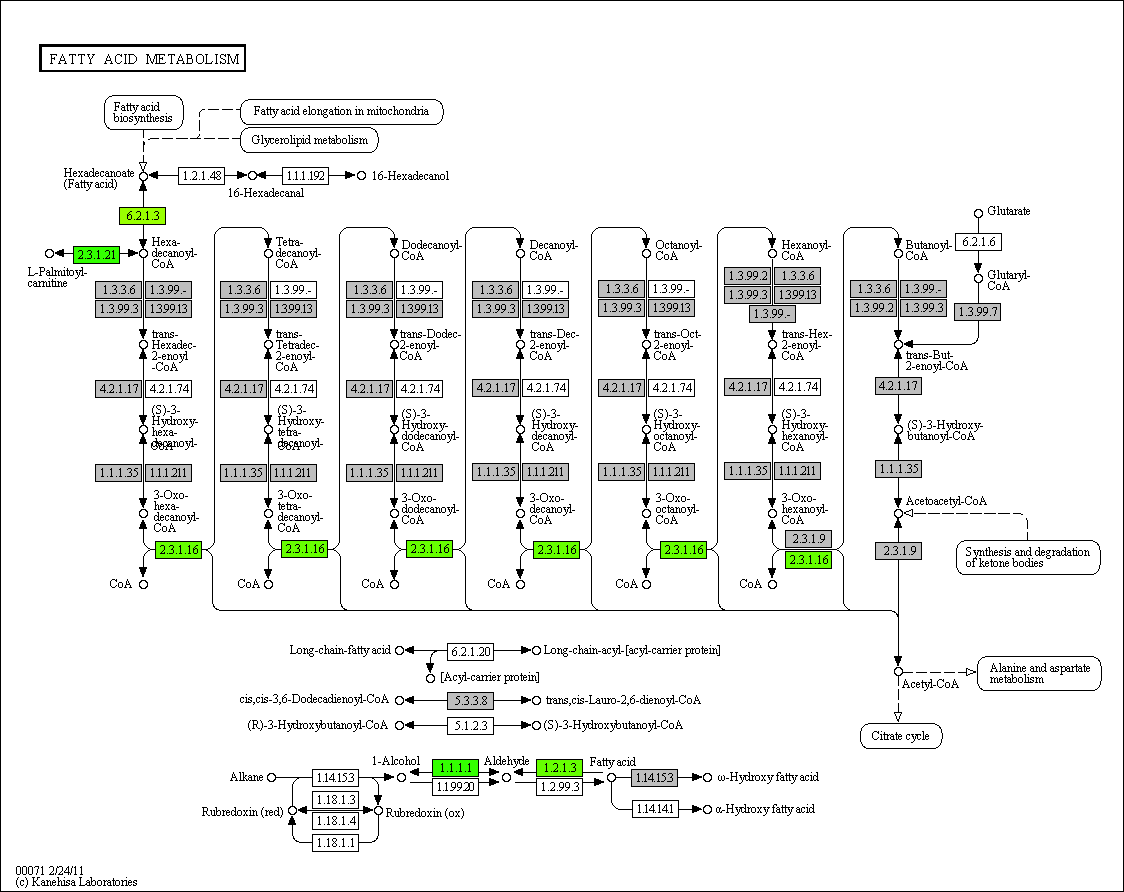


### Glycerolipid metabolism


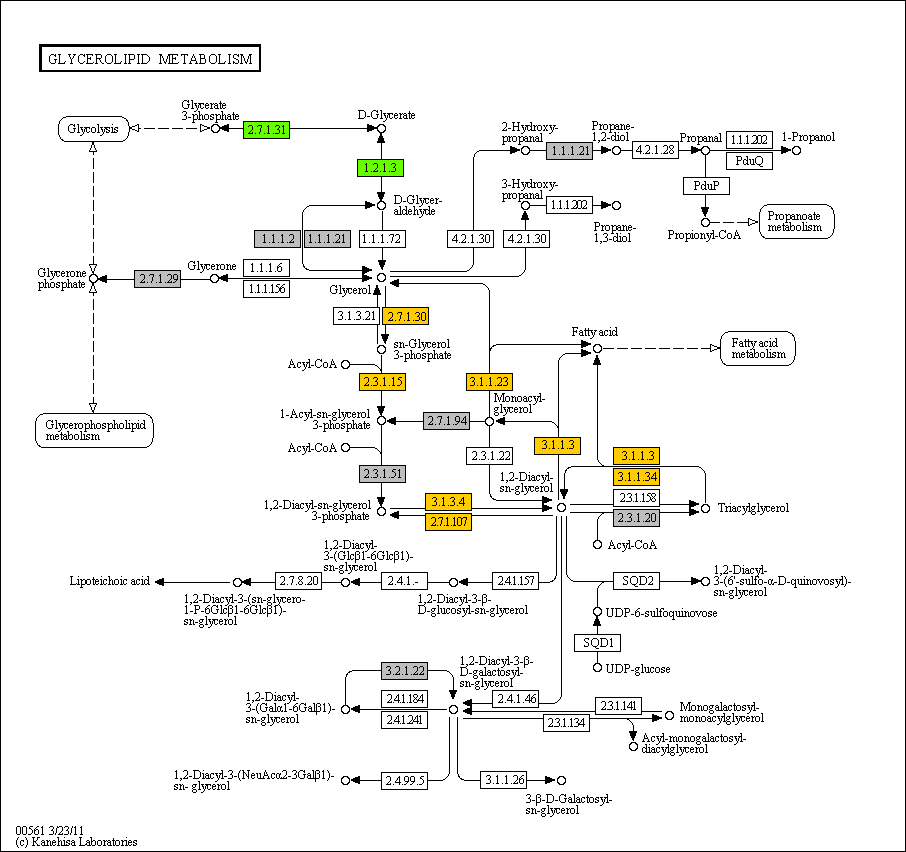


### Glycerophospholipid metabolism


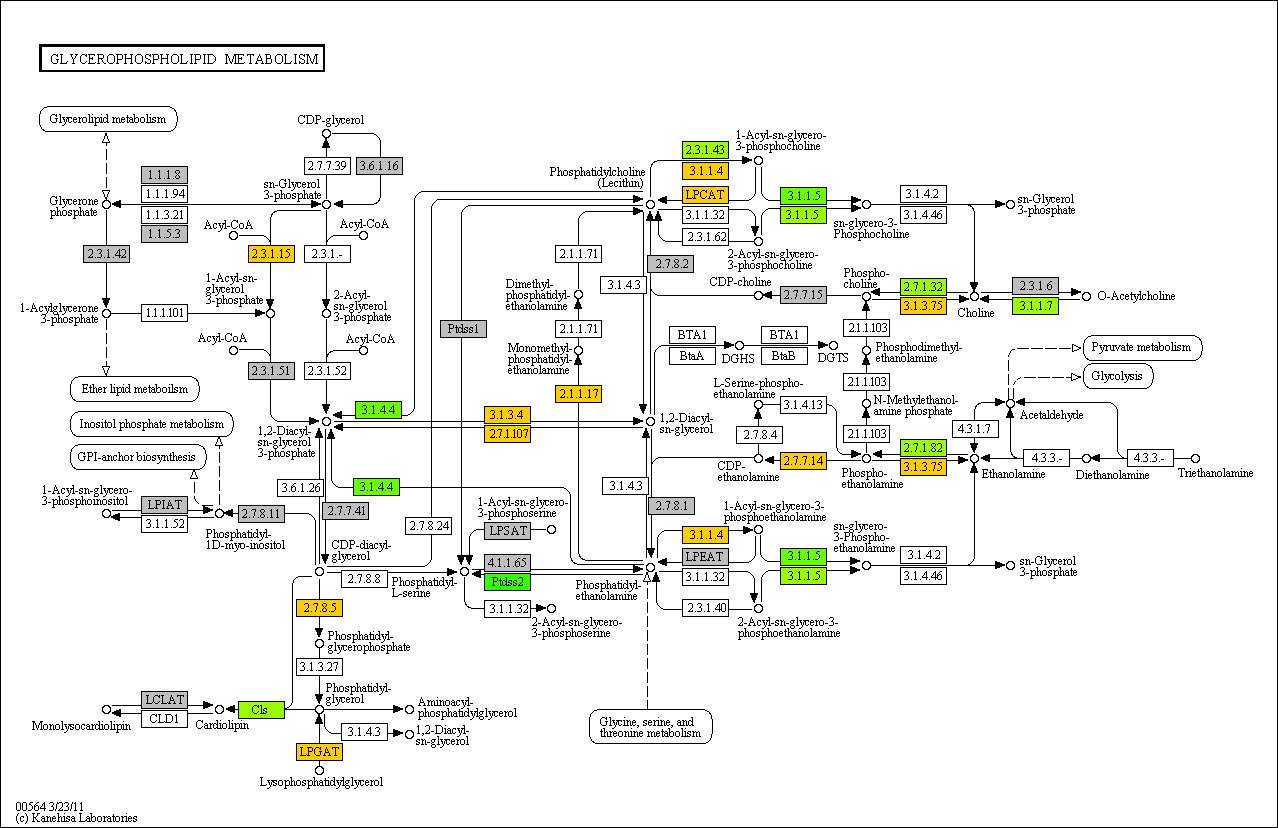


### Primary bile acid biosynthesis


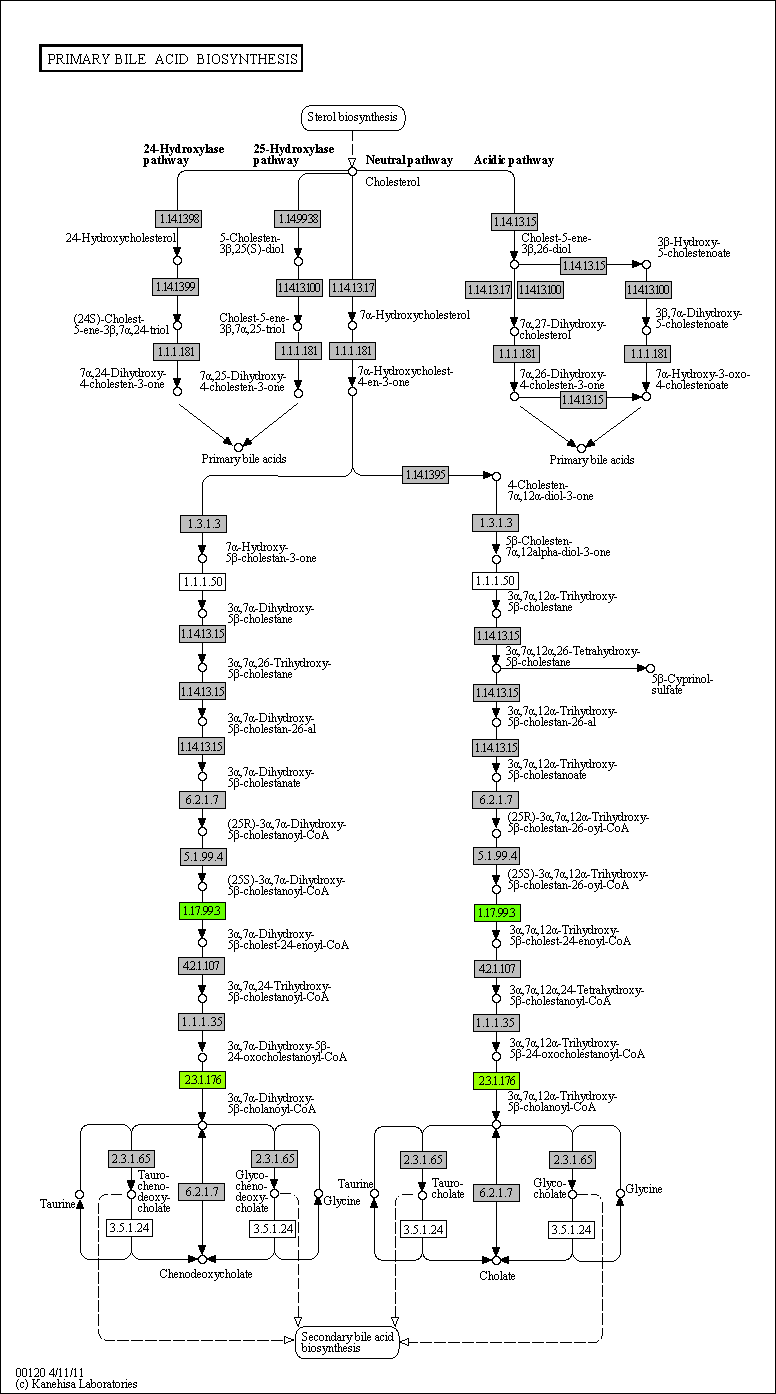


### Sphingolipid metabolism


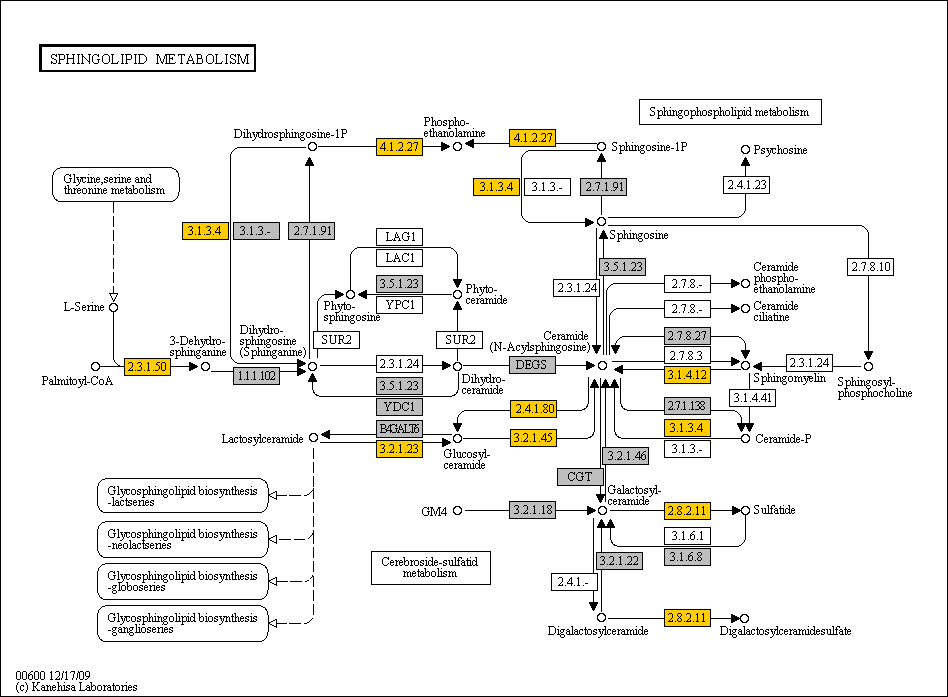


### Steroid biosynthesis


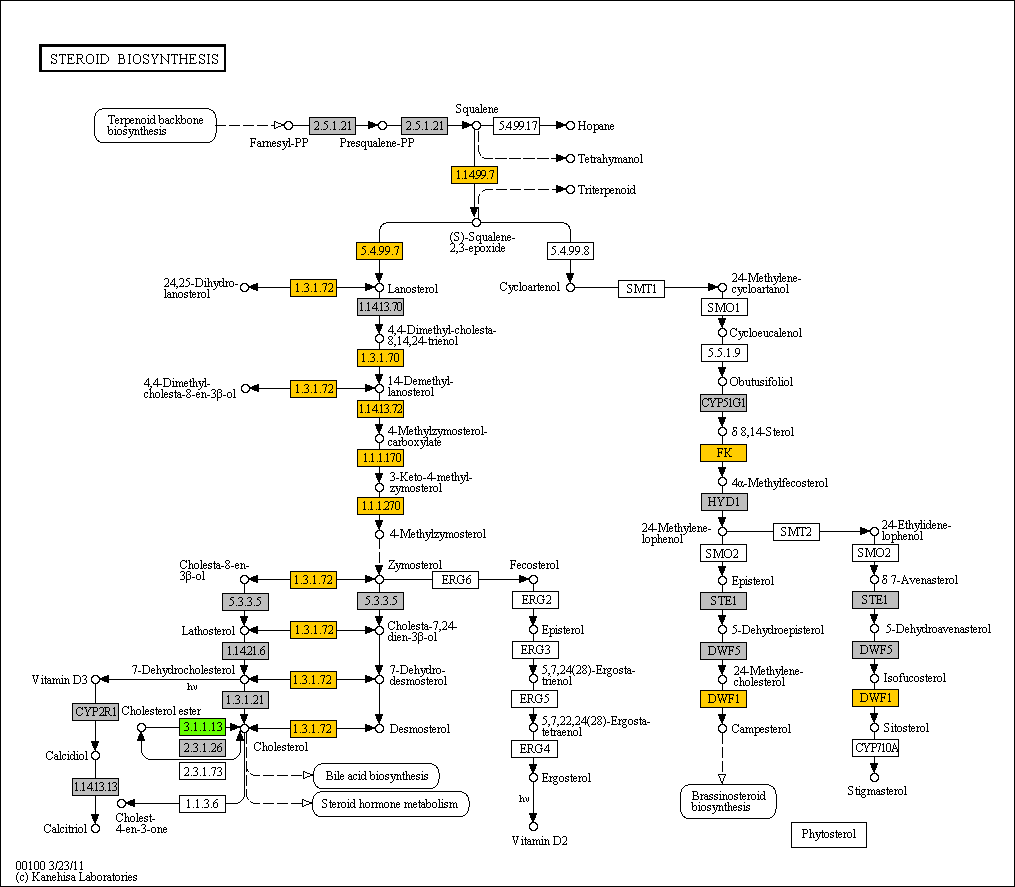


### Steroid hormone biosynthesis


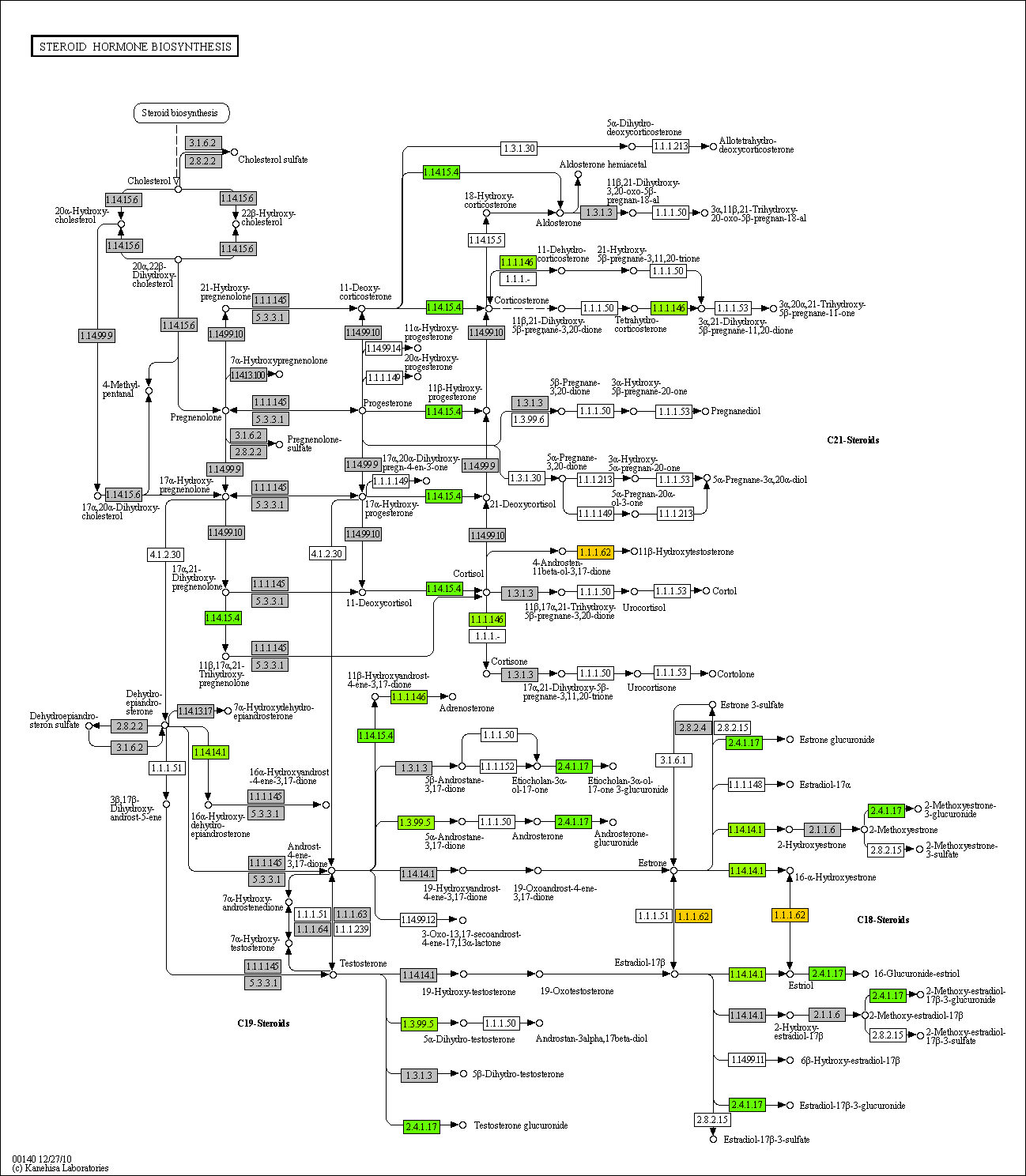


### Synthesis and degradation of ketone bodies


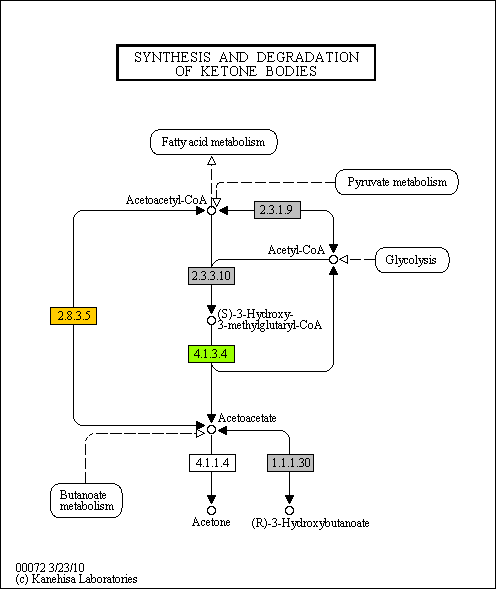


# 1. Metabolism

## 1.4 Nucleotide Metabolism

### Purine metabolism


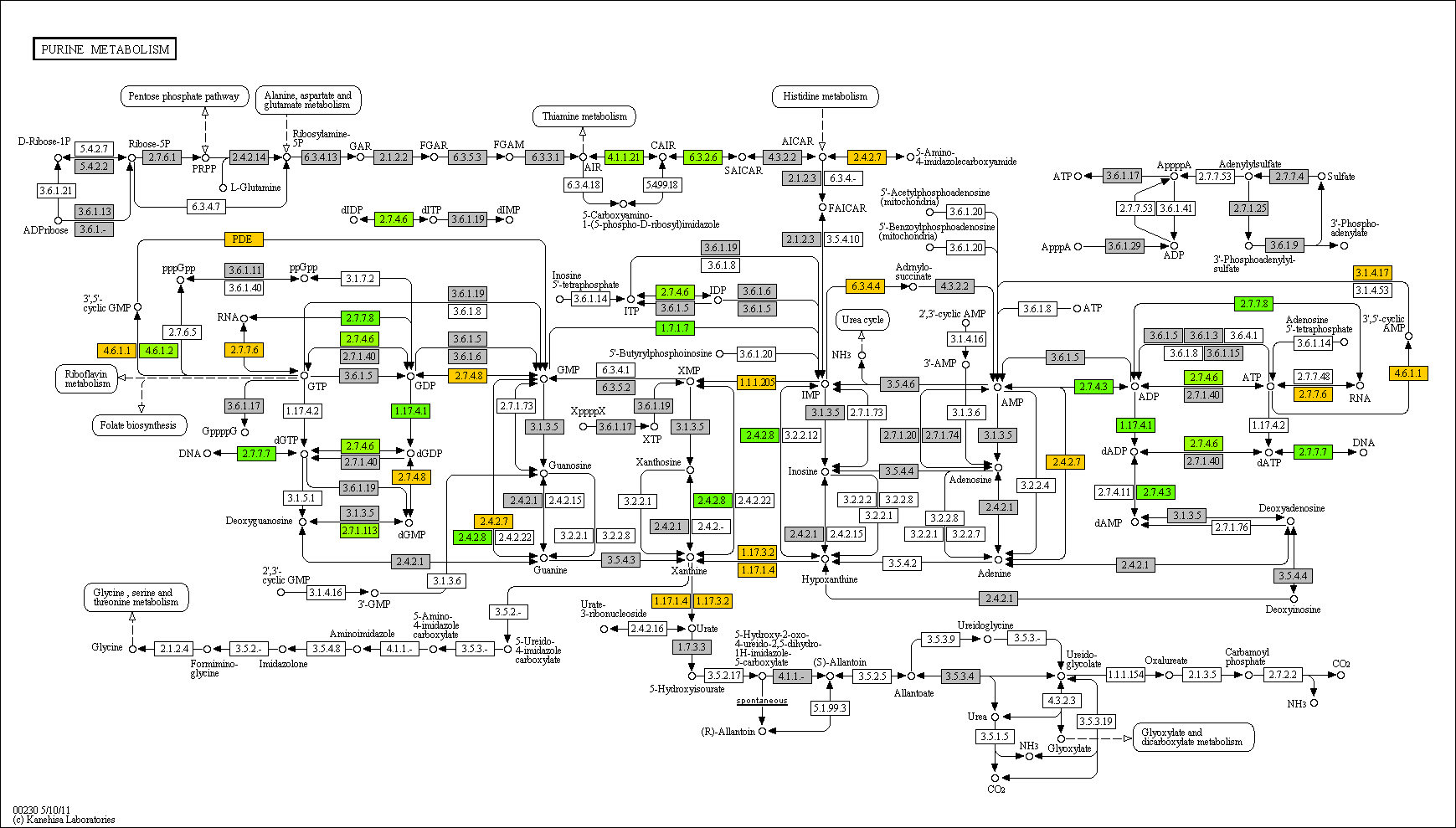


### Pyrimidine metabolism


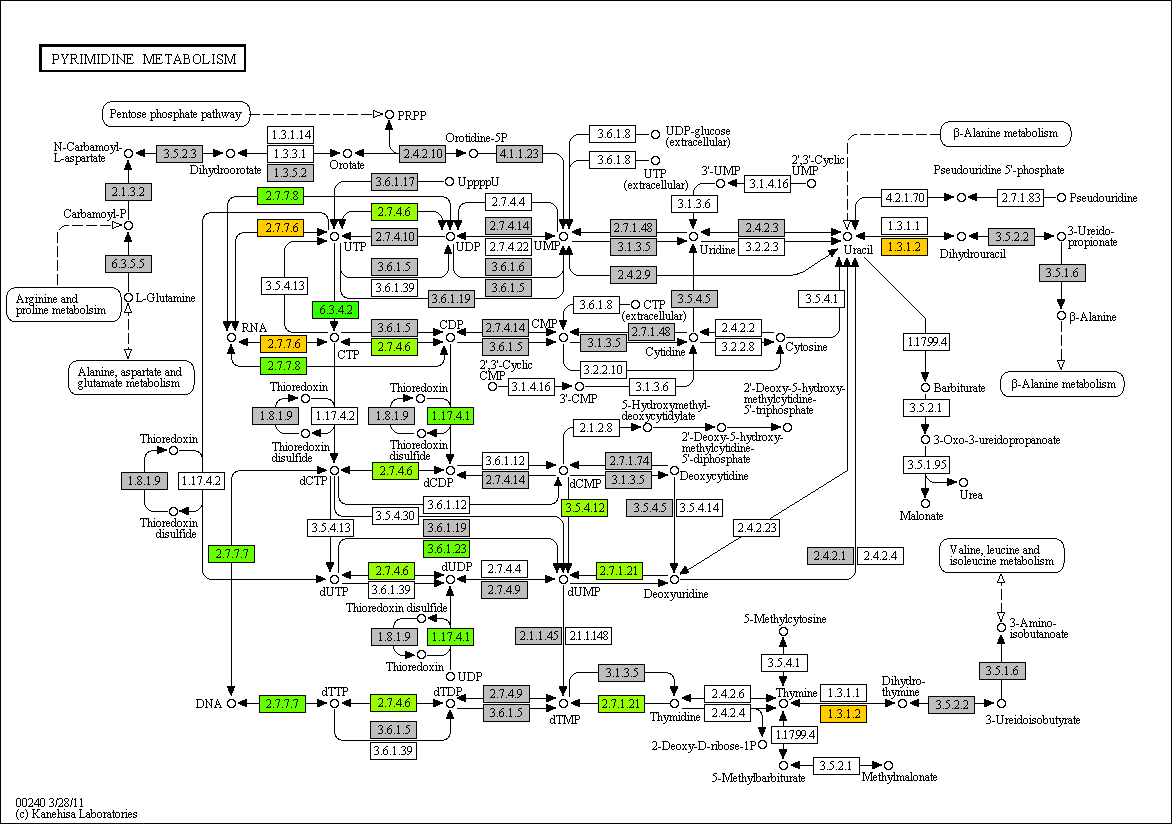


# 1. Metabolism

## 1.5 Amino Acid Metabolism

### Alanine, aspartate and glutamate metabolism


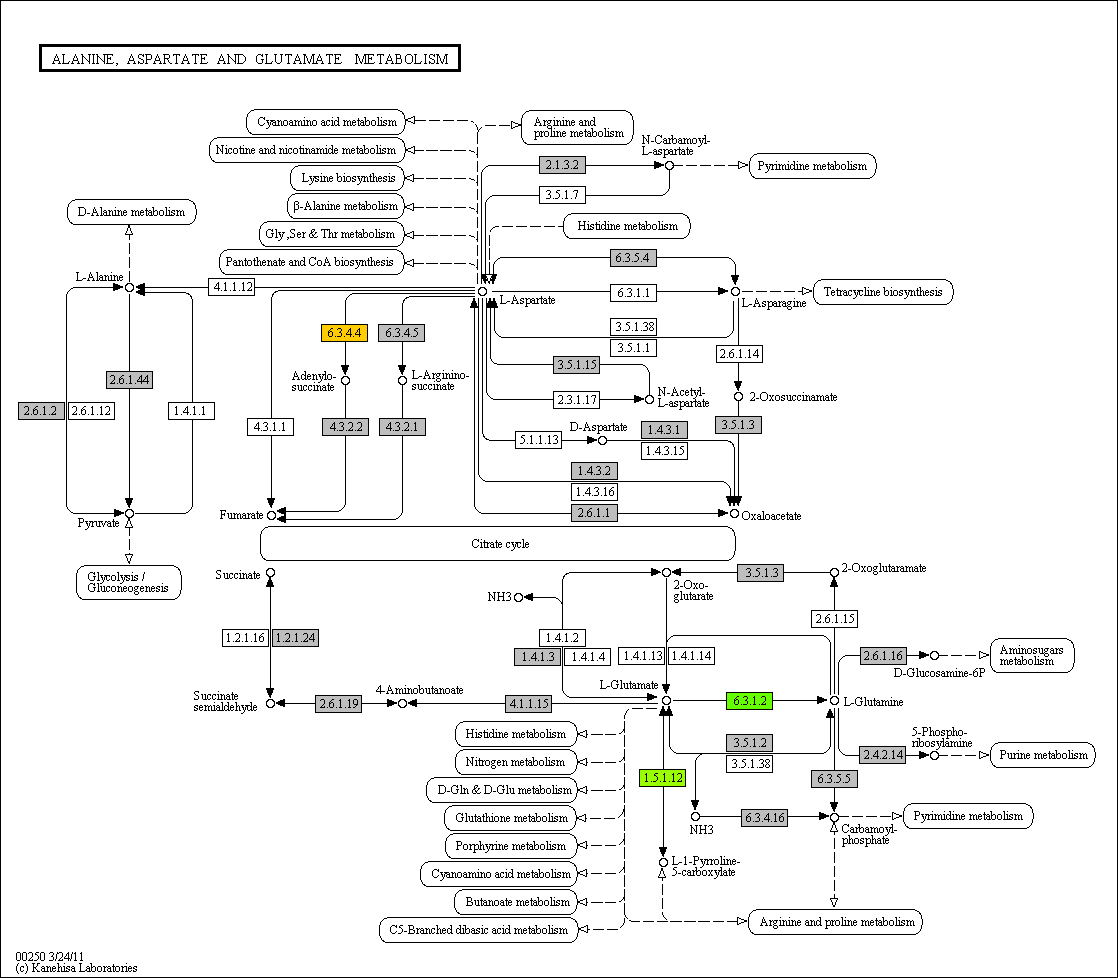


### Arginine and proline metabolism


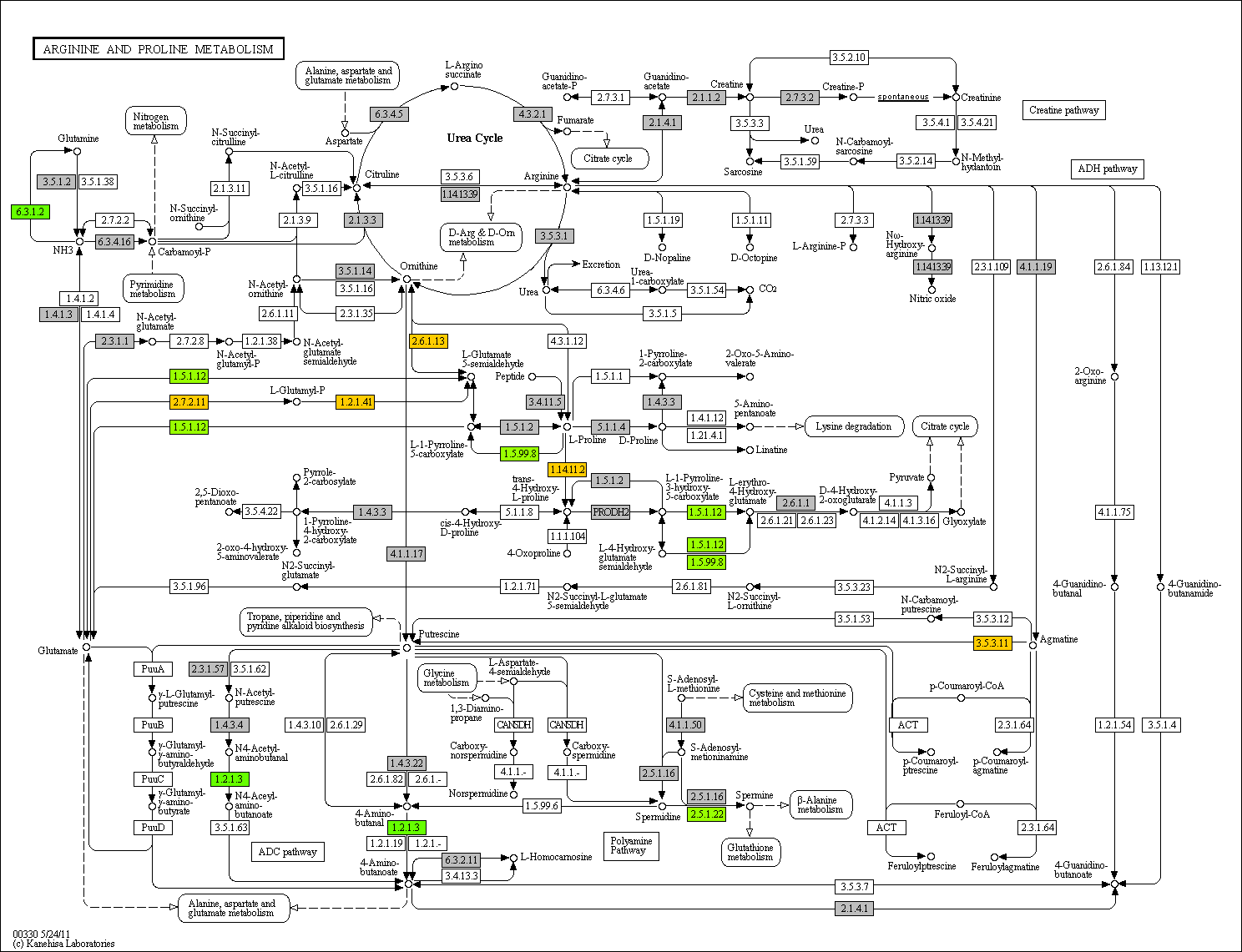


### Cysteine and methionine metabolism


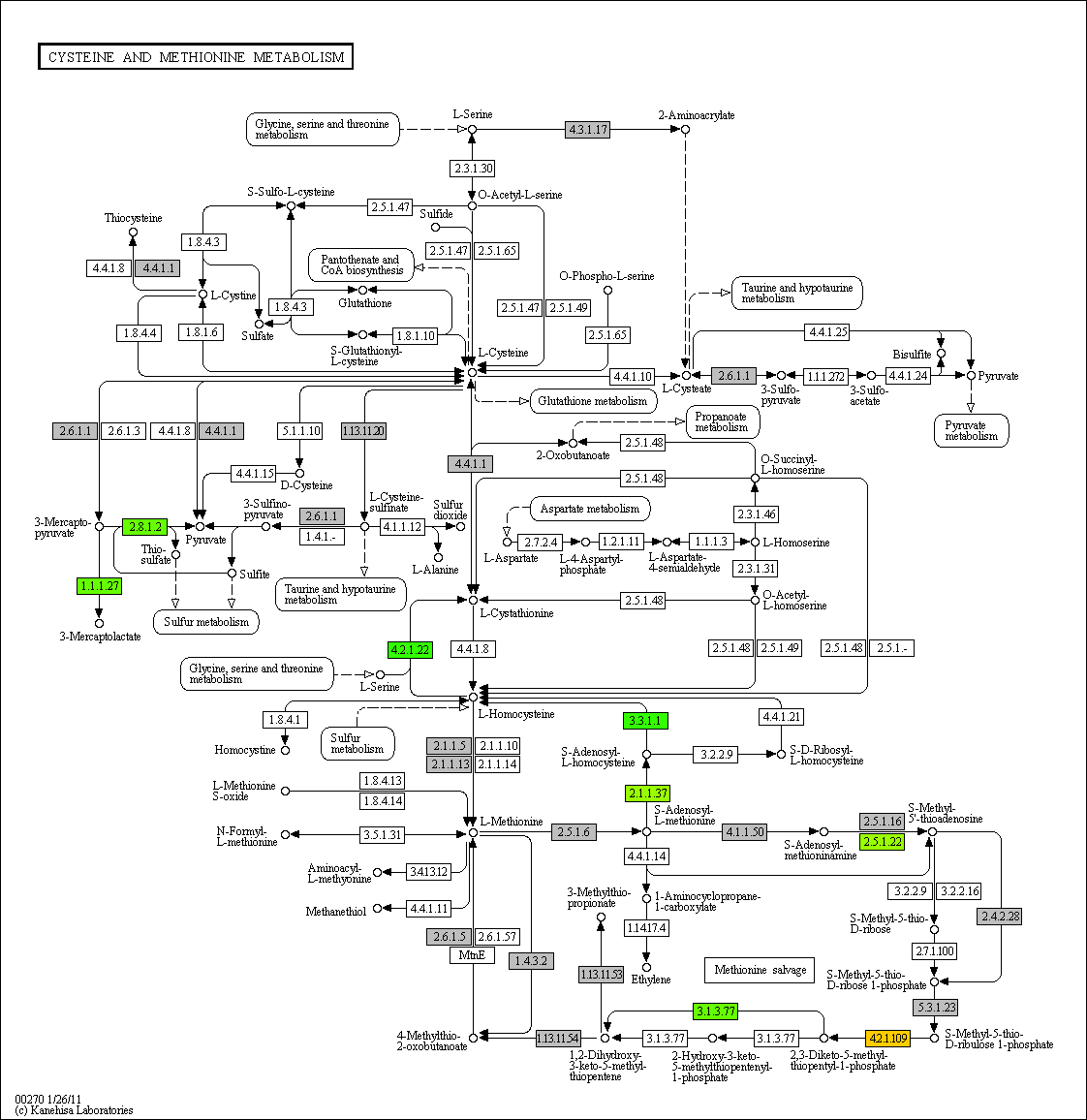


### Glycine, serine and threonine metabolism


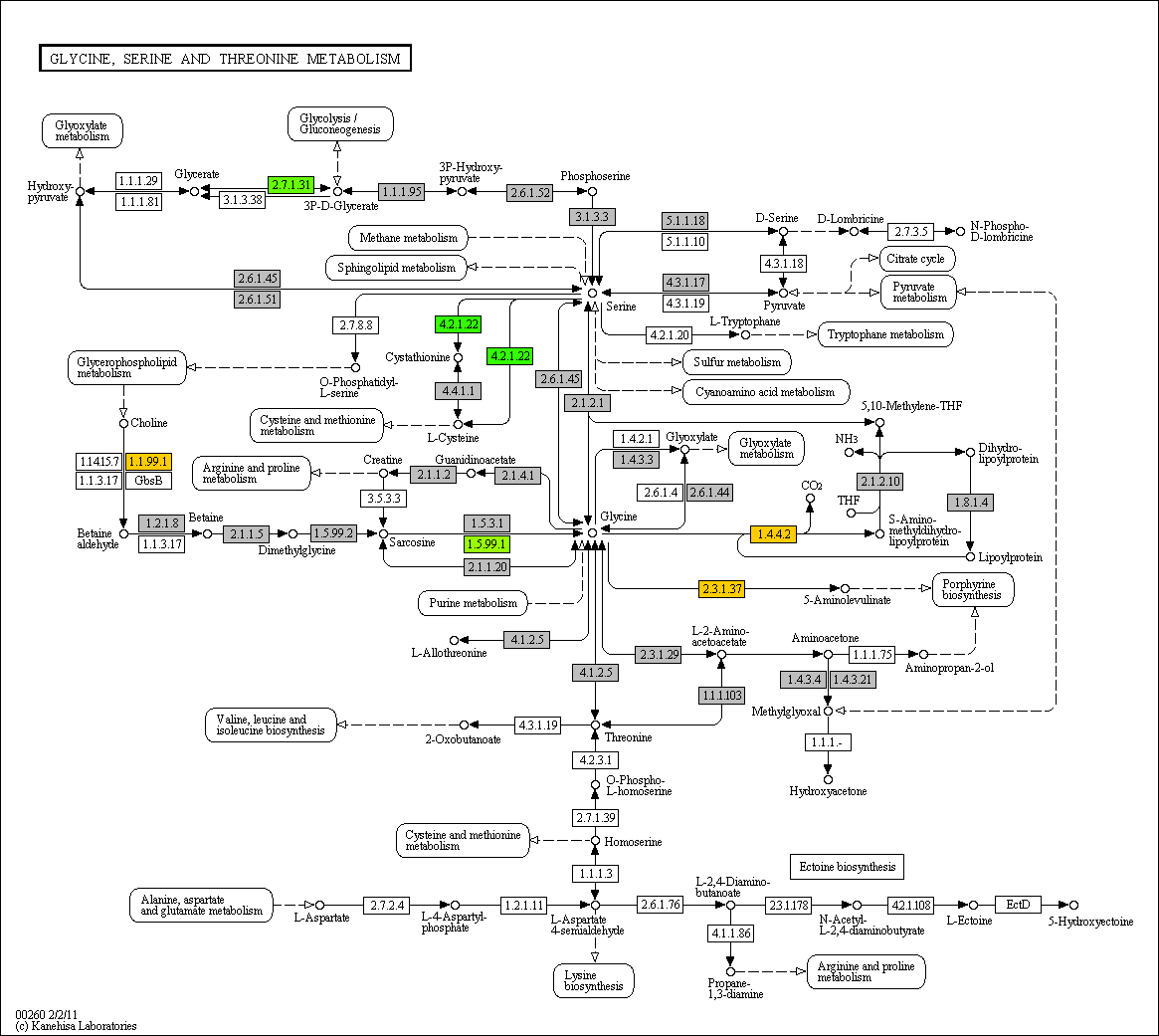


### Histidine metabolism


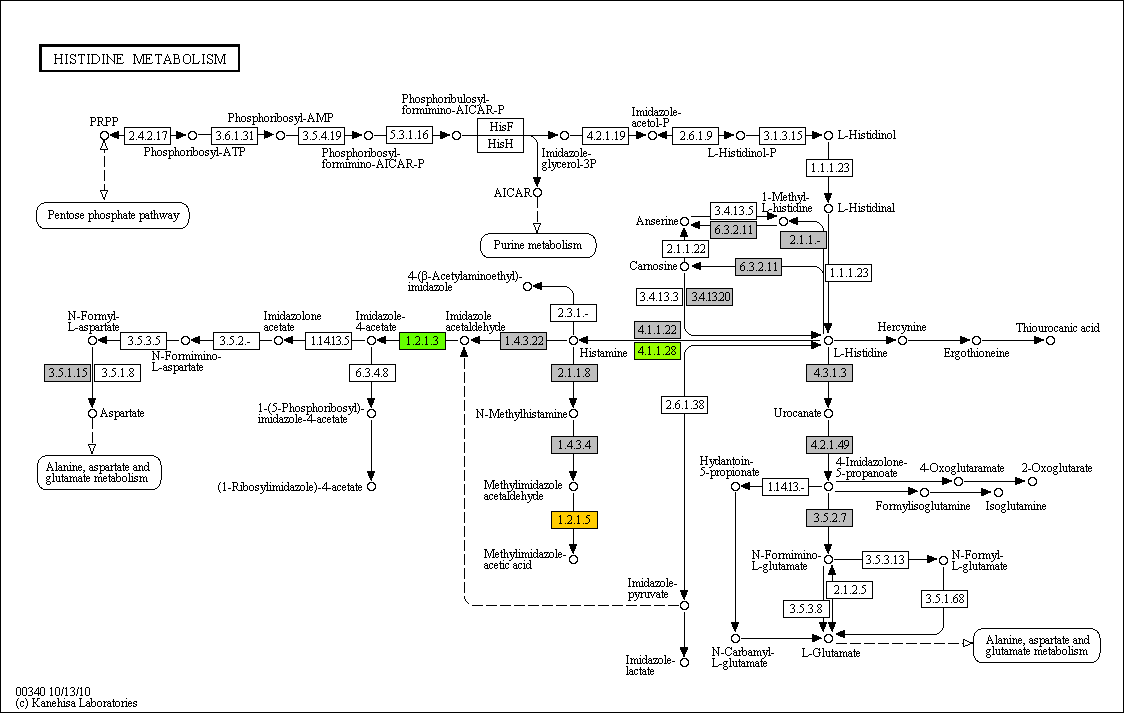


### Lysine degradation


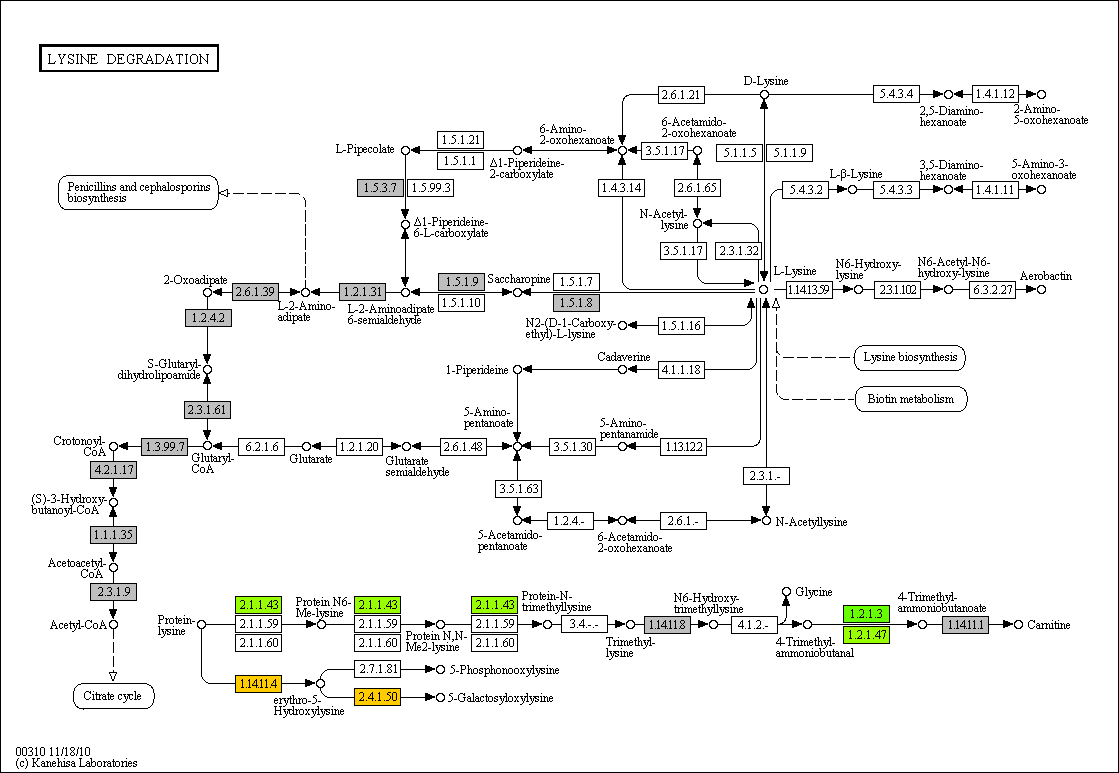


### Phenylalanine metabolism


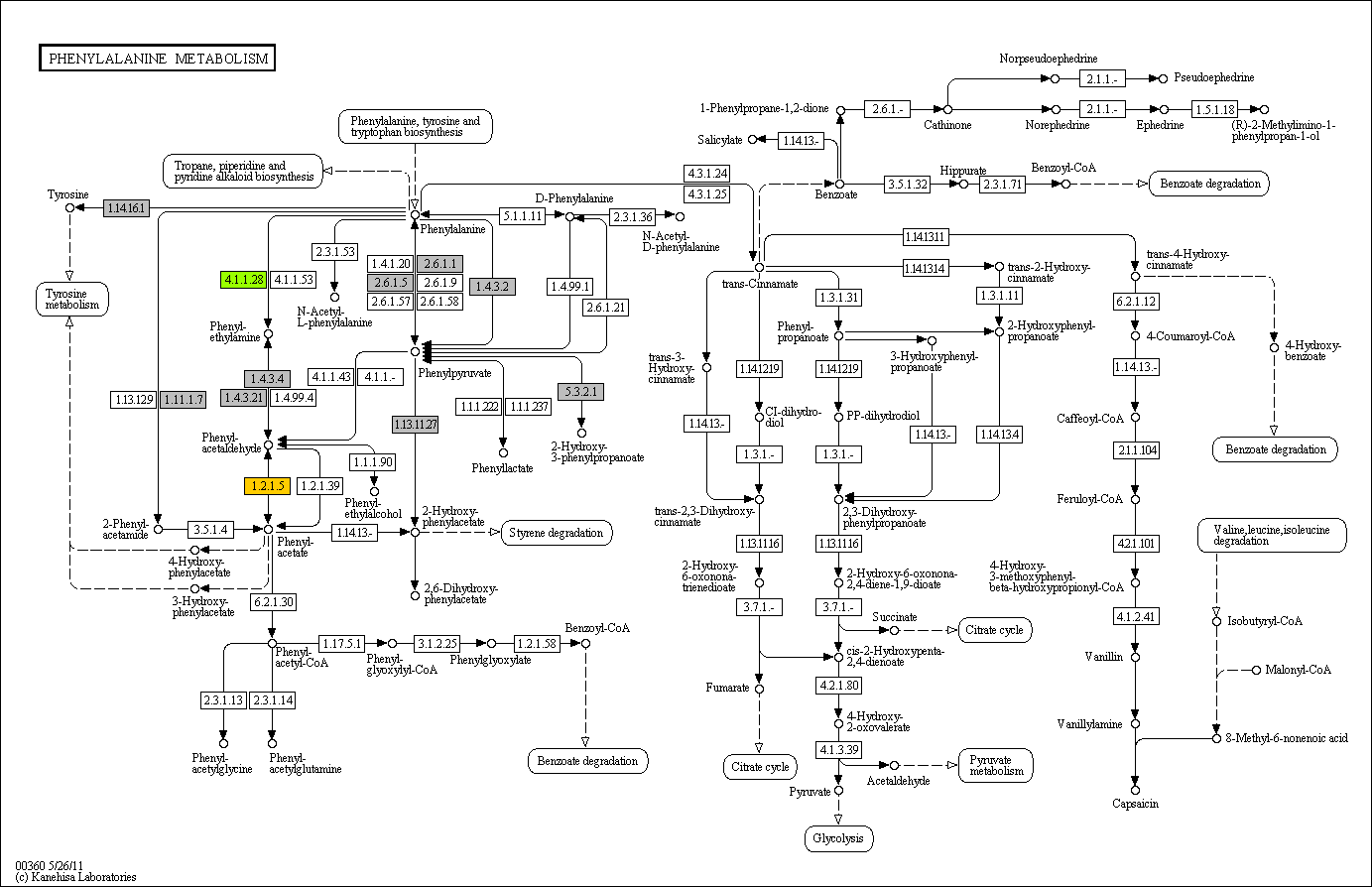


### Tryptophan metabolism


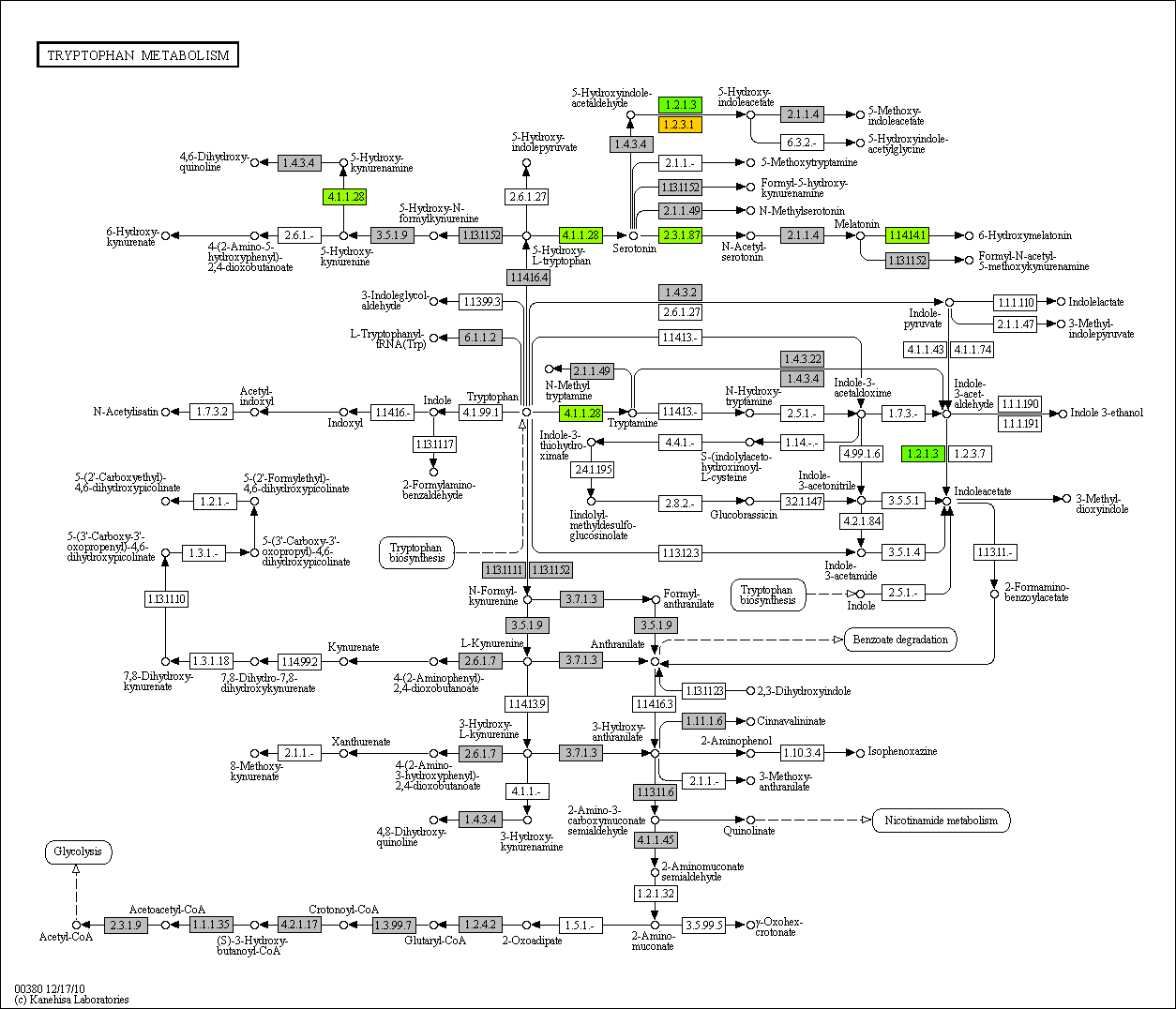


### Tyrosine metabolism


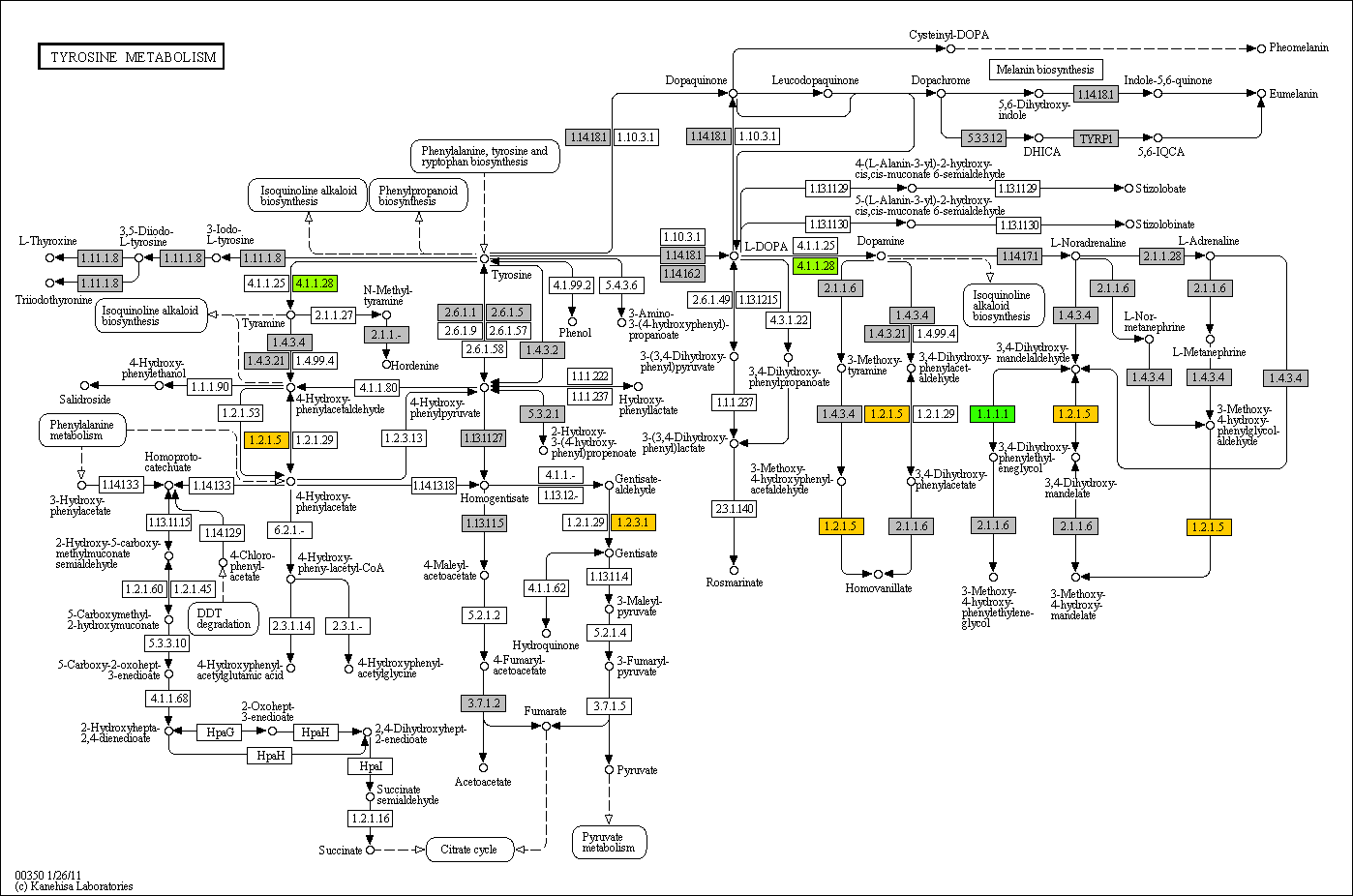


### Valine, leucine and isoleucine biosynthesis


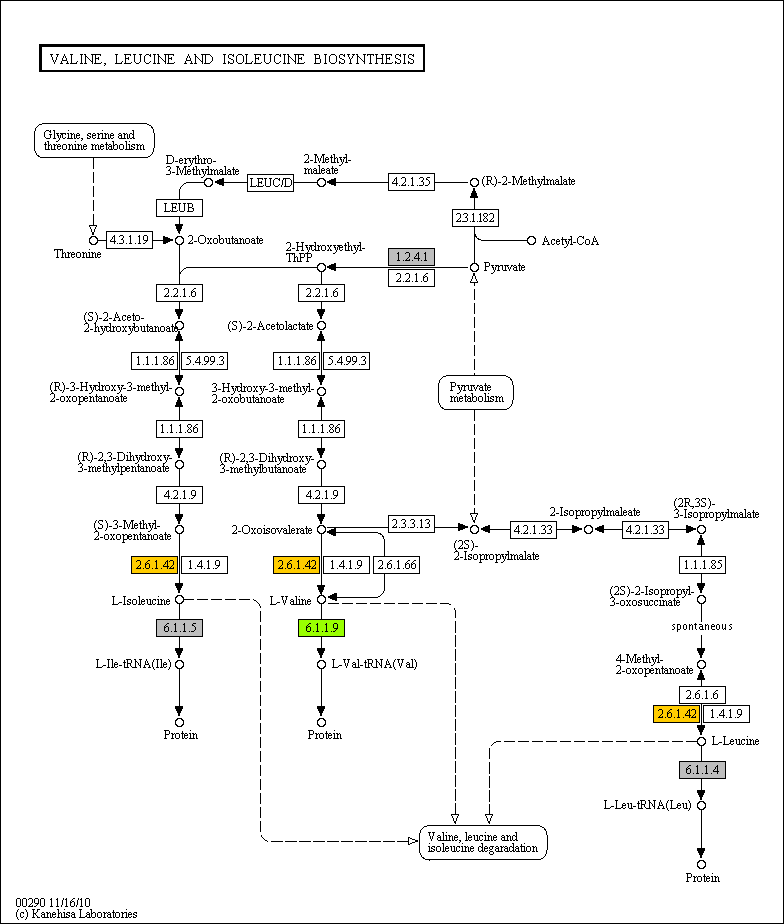


### Valine, leucine and isoleucine degradation


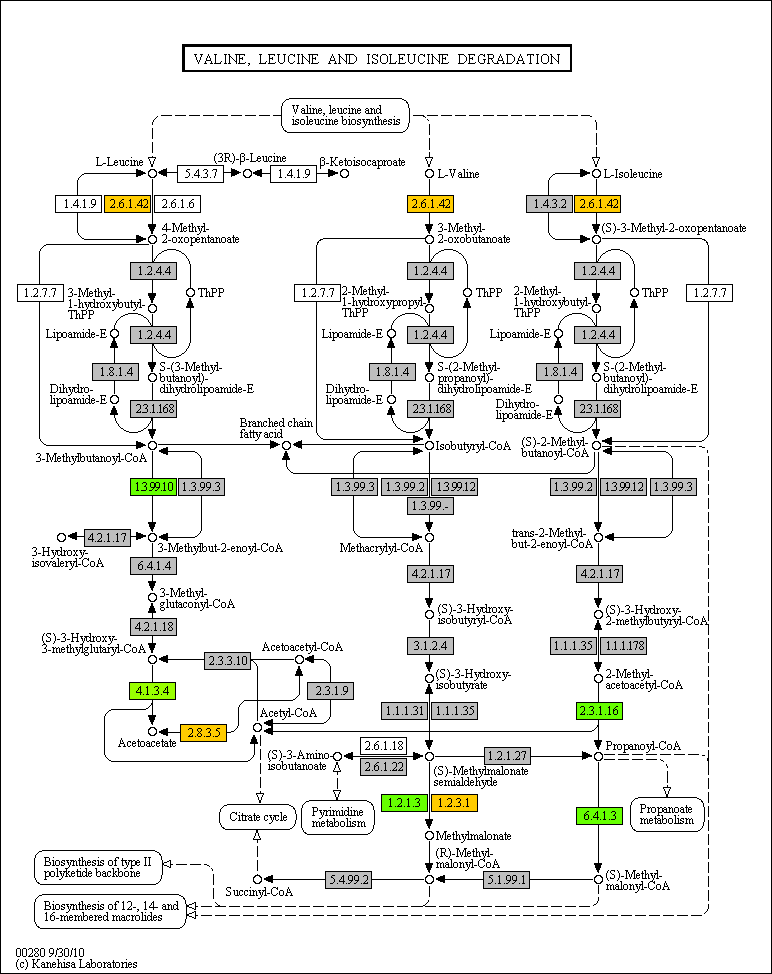


# 1. Metabolism

## 1.6 Metabolism of Other Amino Acids

### beta-Alanine metabolism


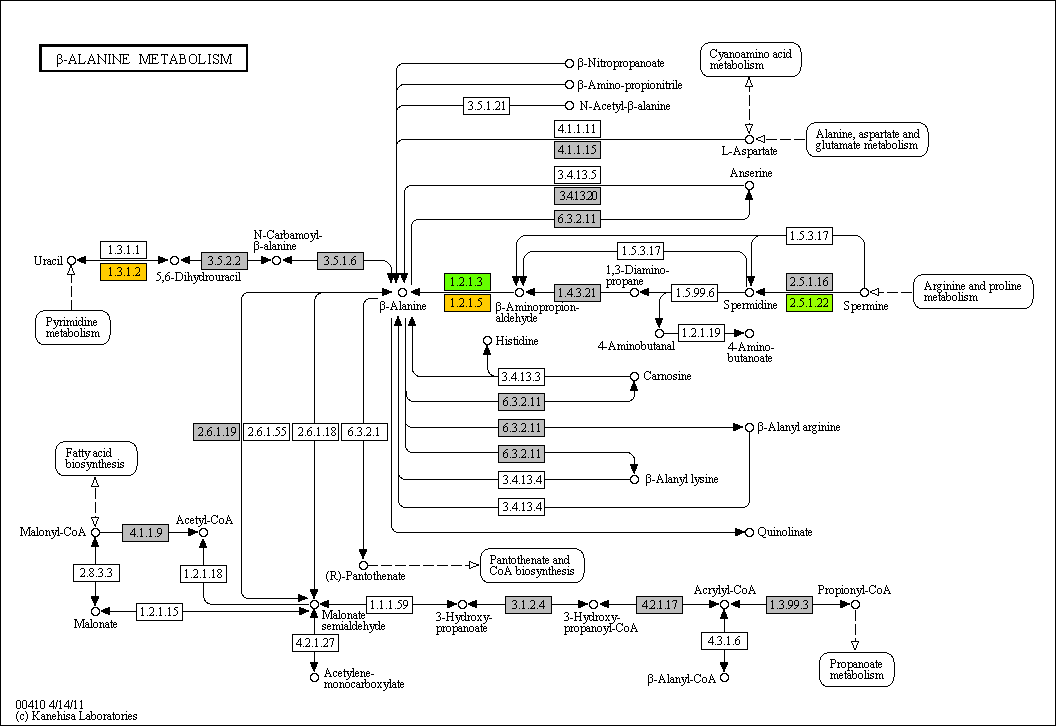


### Cyanoamino acid metabolism


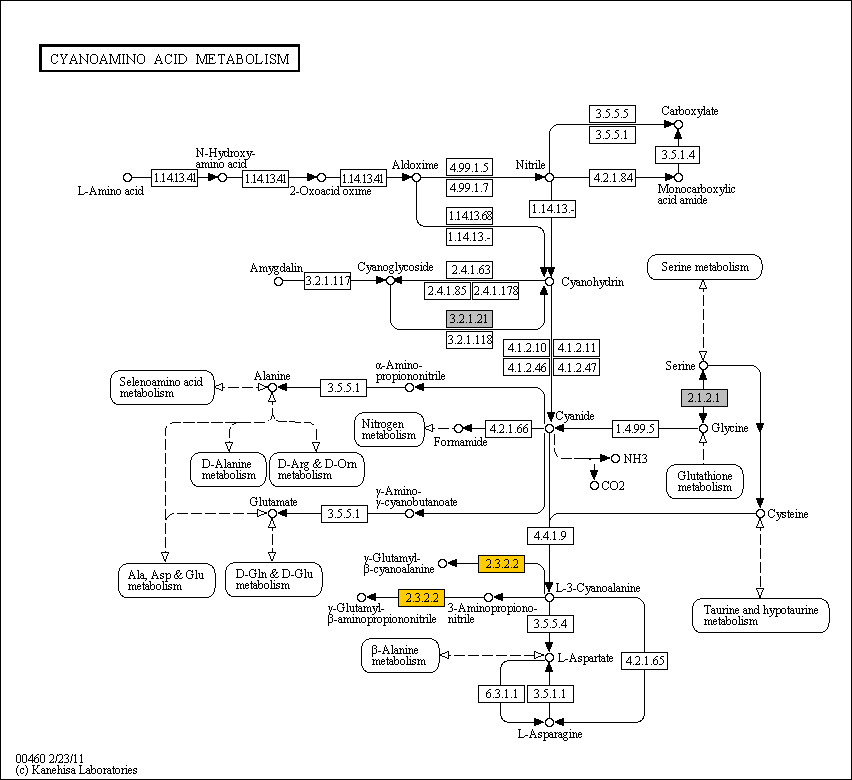


### Glutathione metabolism


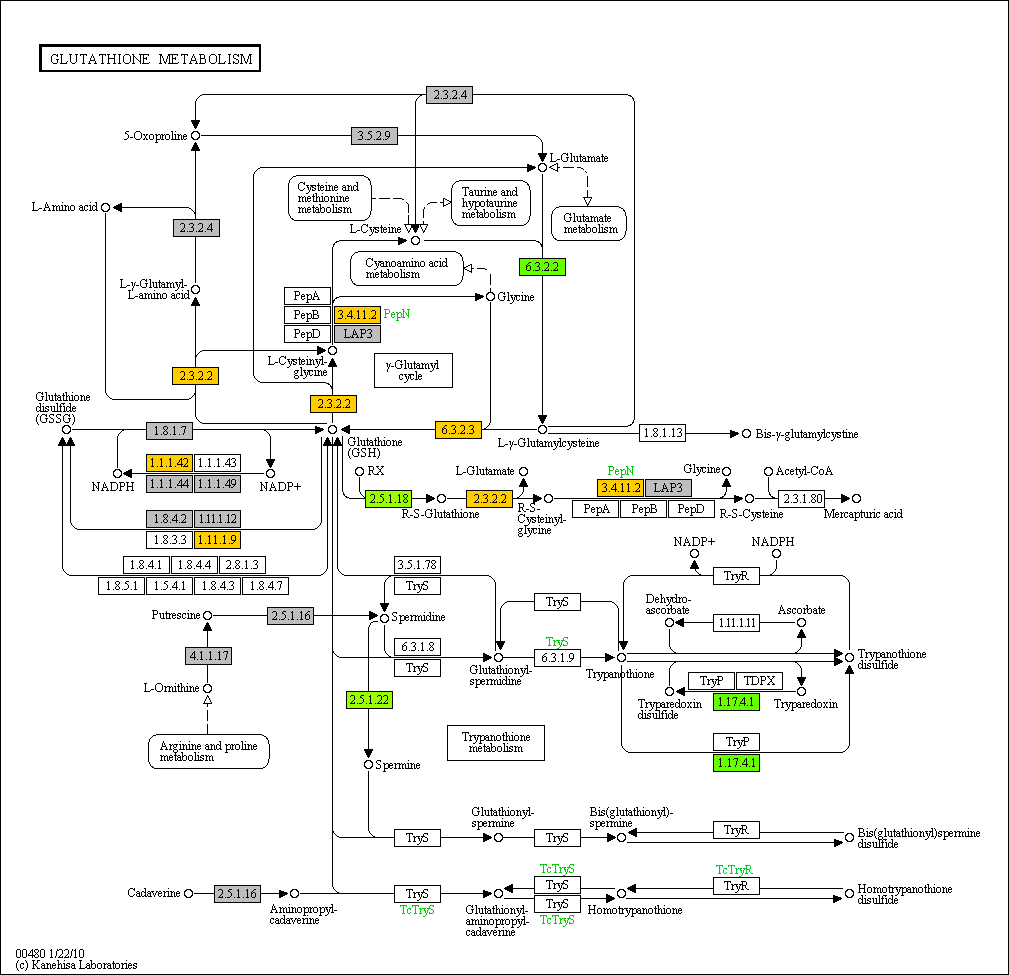


### Taurine and hypotaurine metabolism


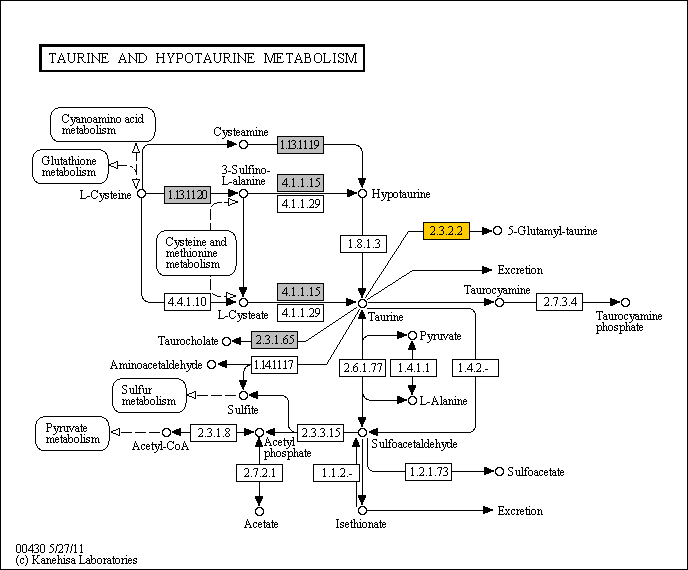


# 1. Metabolism

## 1.7 Glycan Biosynthesis and Metabolism

### Glycosaminoglycan biosynthesis - chondroitin sulfate


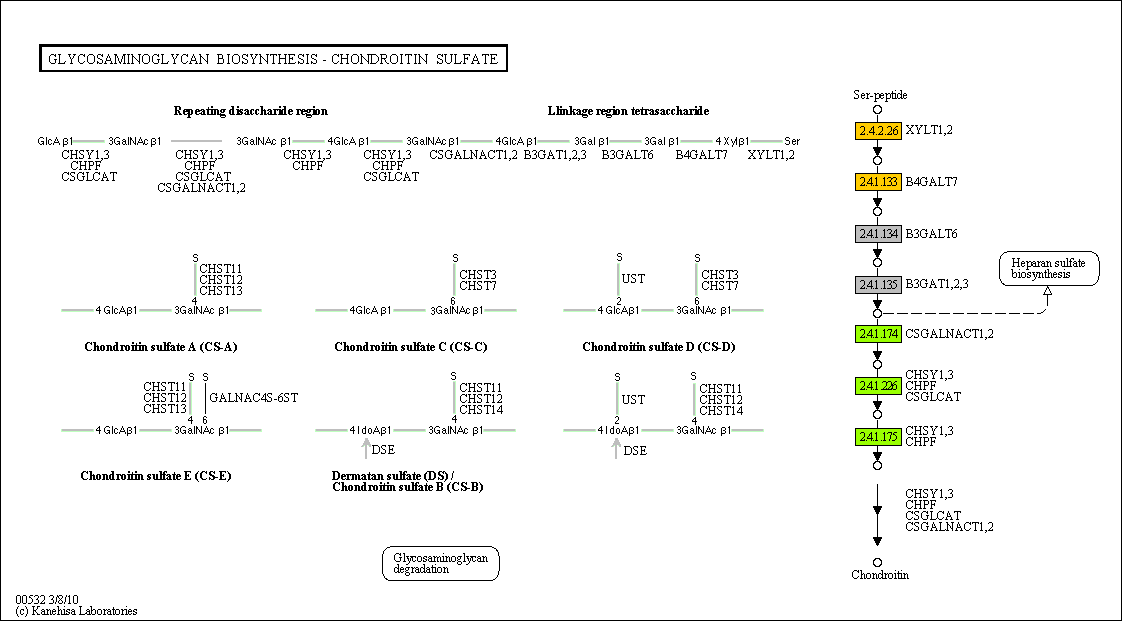


### Glycosaminoglycan biosynthesis - keratan sulfate


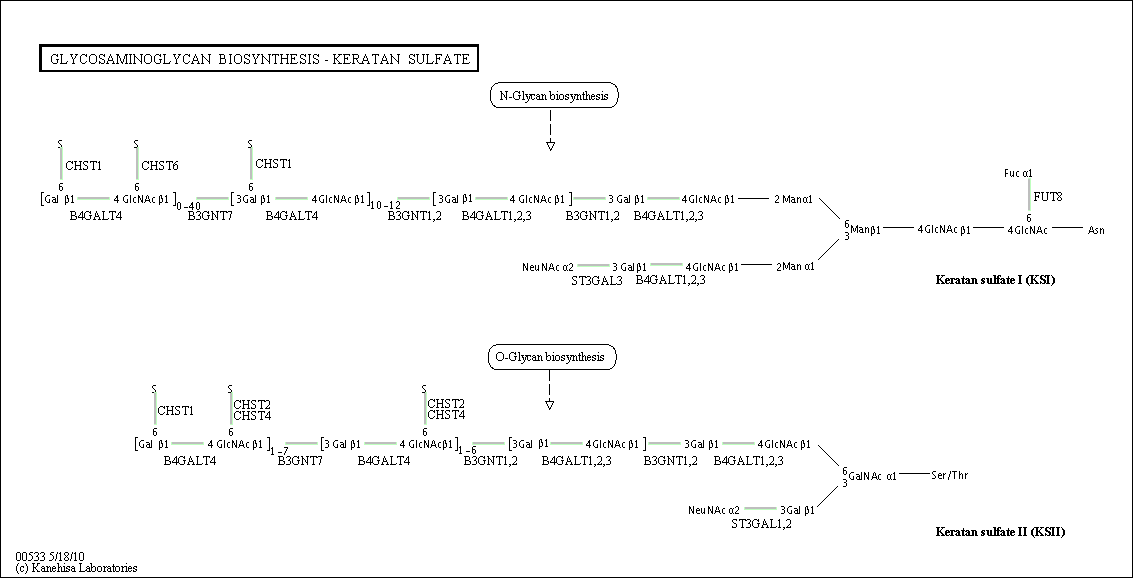


### Glycosaminoglycan degradation


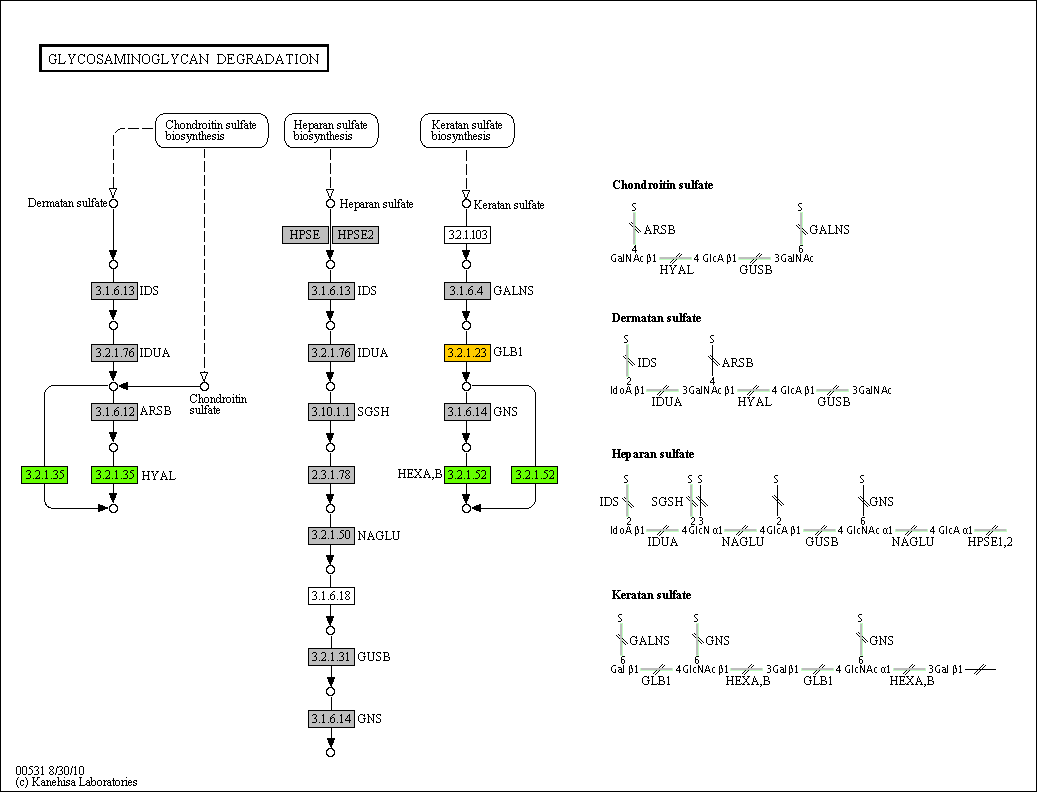


### Glycosphingolipid biosynthesis - ganglio series


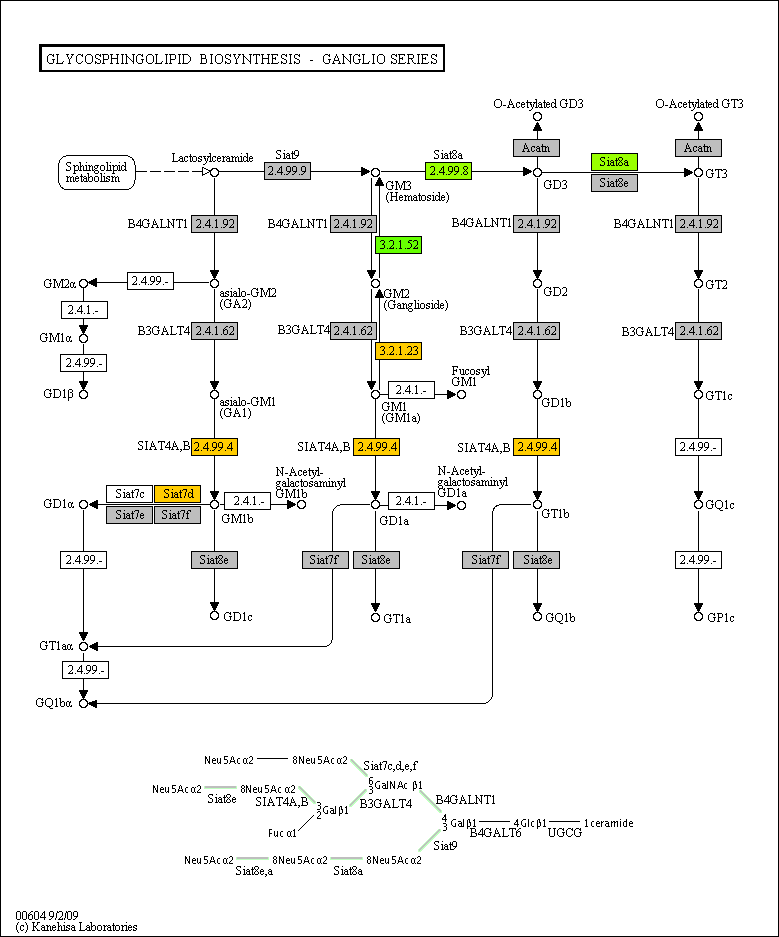


### Glycosphingolipid biosynthesis - globo series


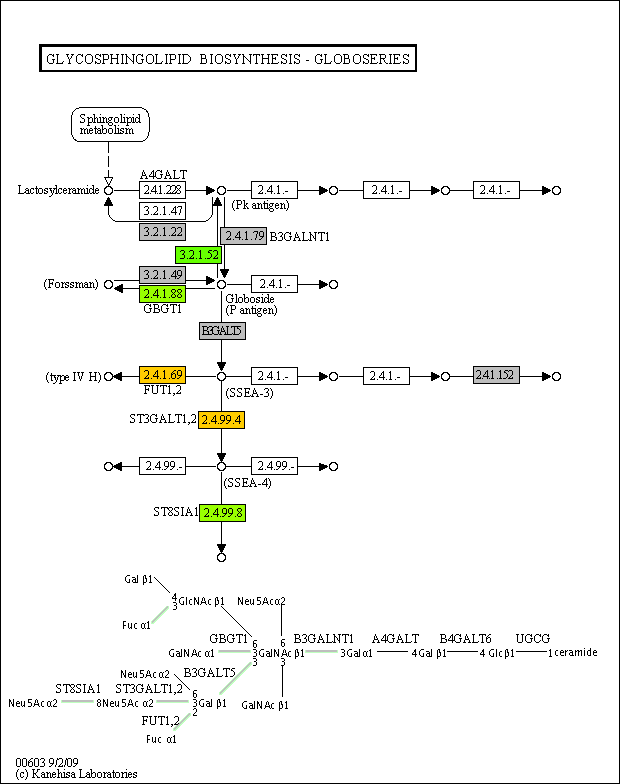


### Glycosphingolipid biosynthesis - lacto and neolacto series


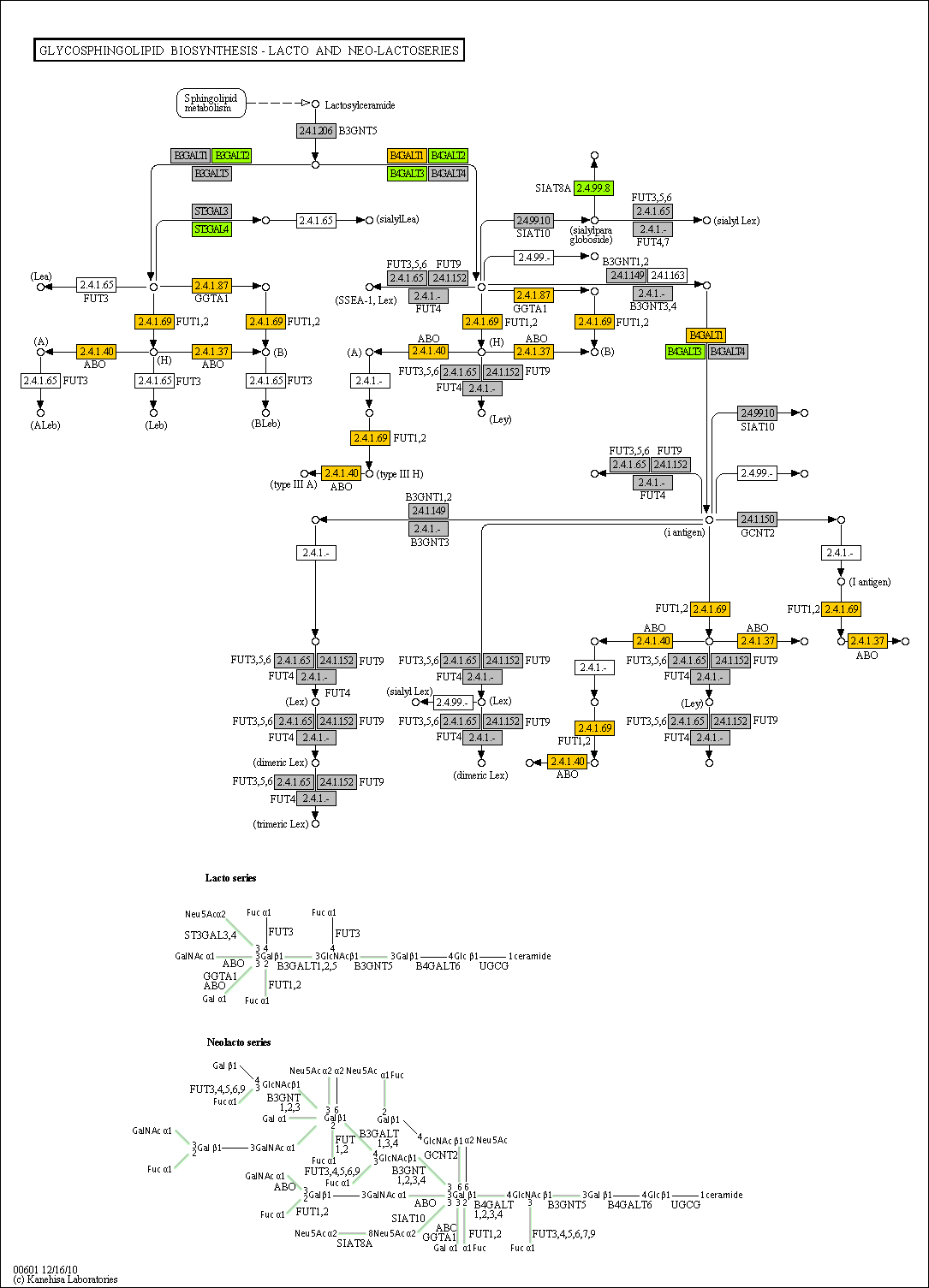


### Glycosylphosphatidylinositol(GPI)-anchor biosynthesis


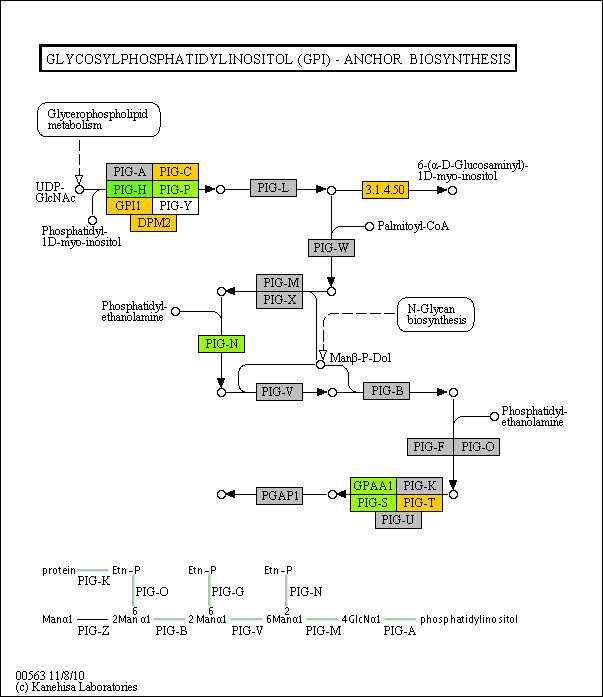


### N-Glycan biosynthesis


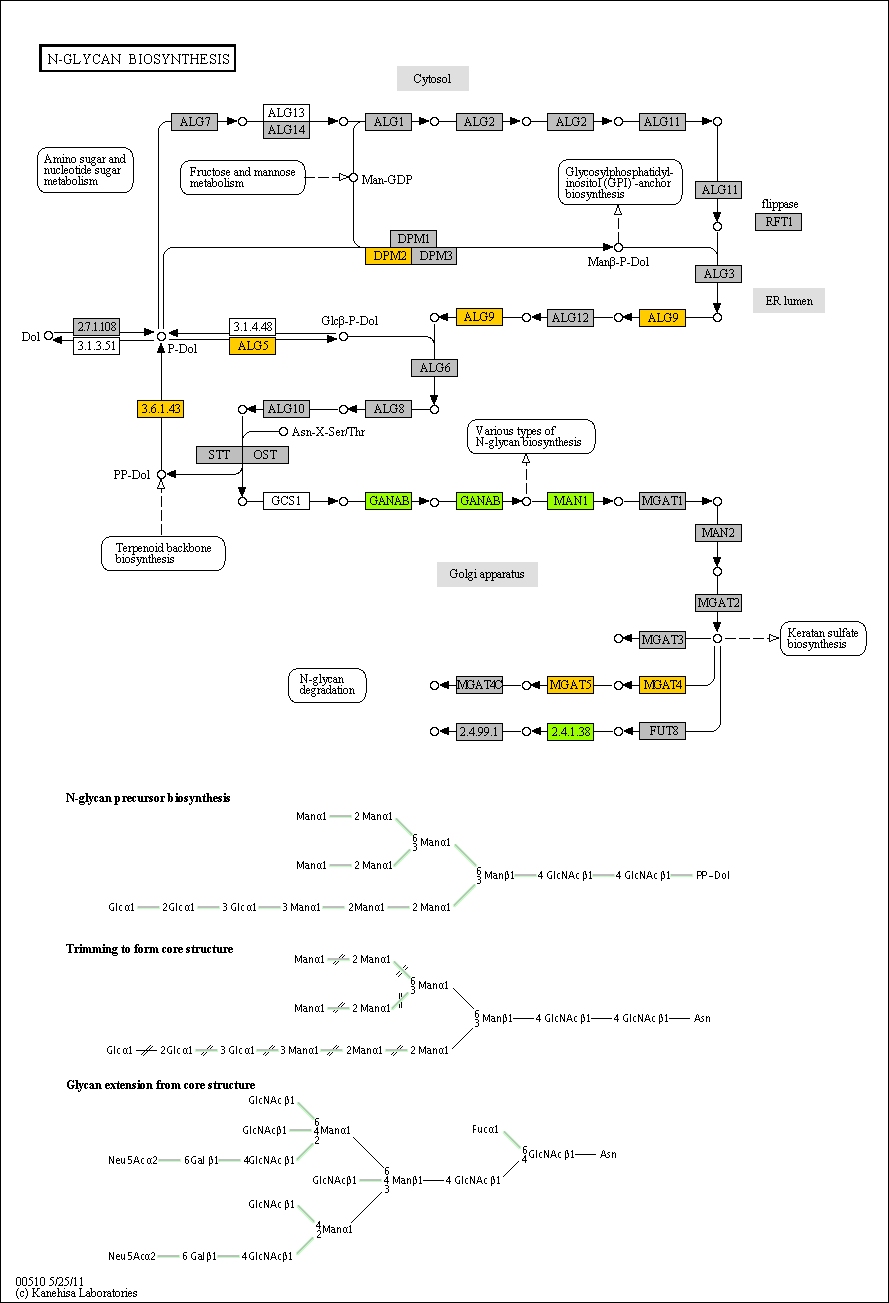


### O-Glycan biosynthesis


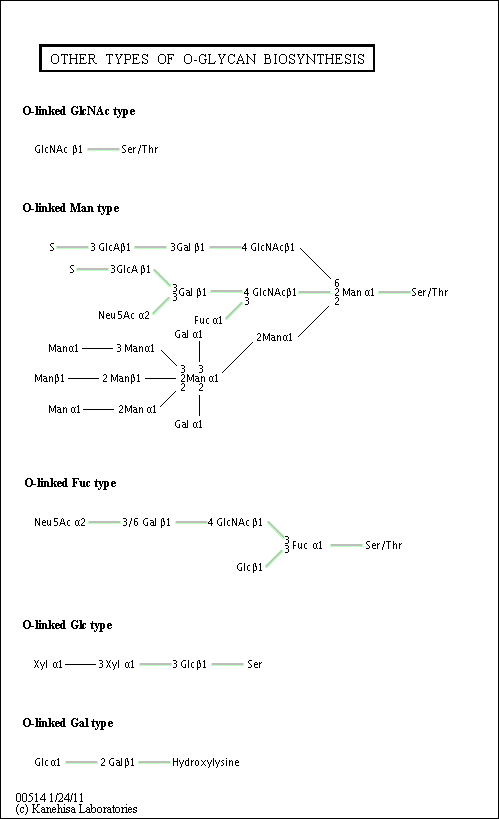


Other glycan degradation
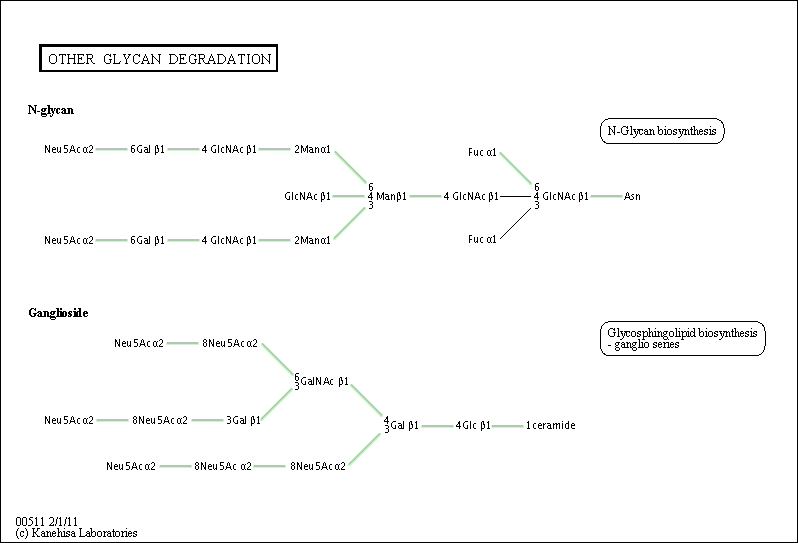


# 1. Metabolism

## 1.8 Metabolism of Cofactors and Vitamins

### Biotin metabolism


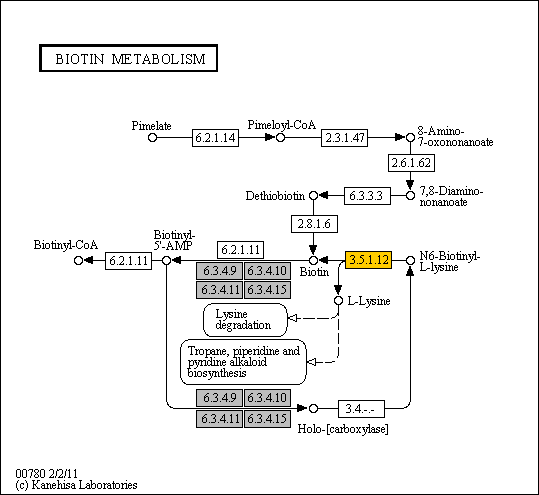


### Folate biosynthesis


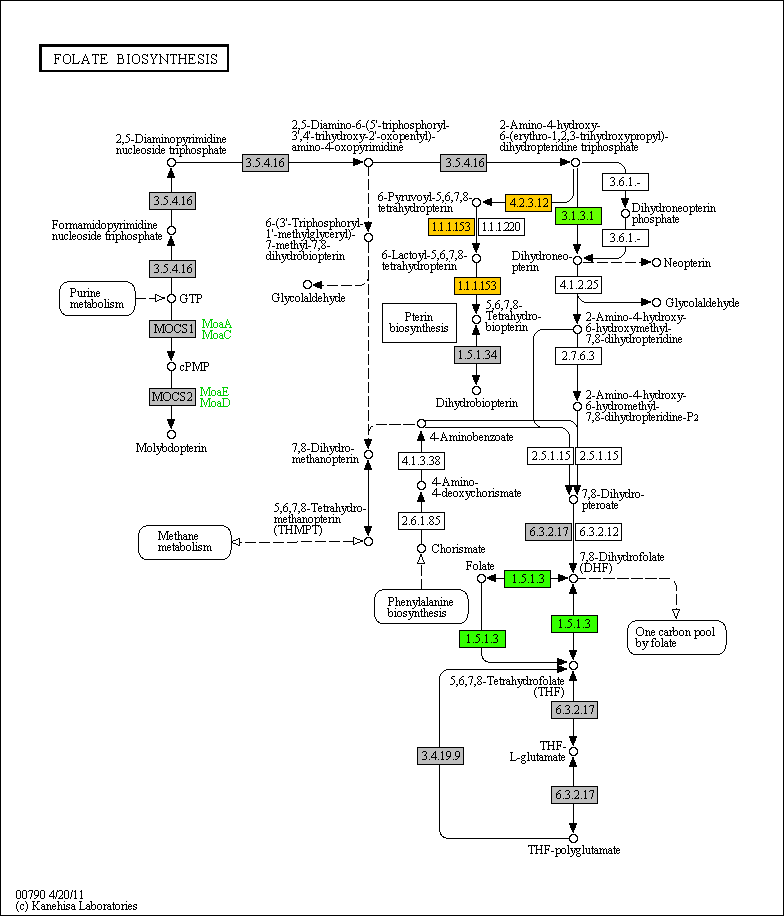


### Nicotinate and nicotinamide metabolism


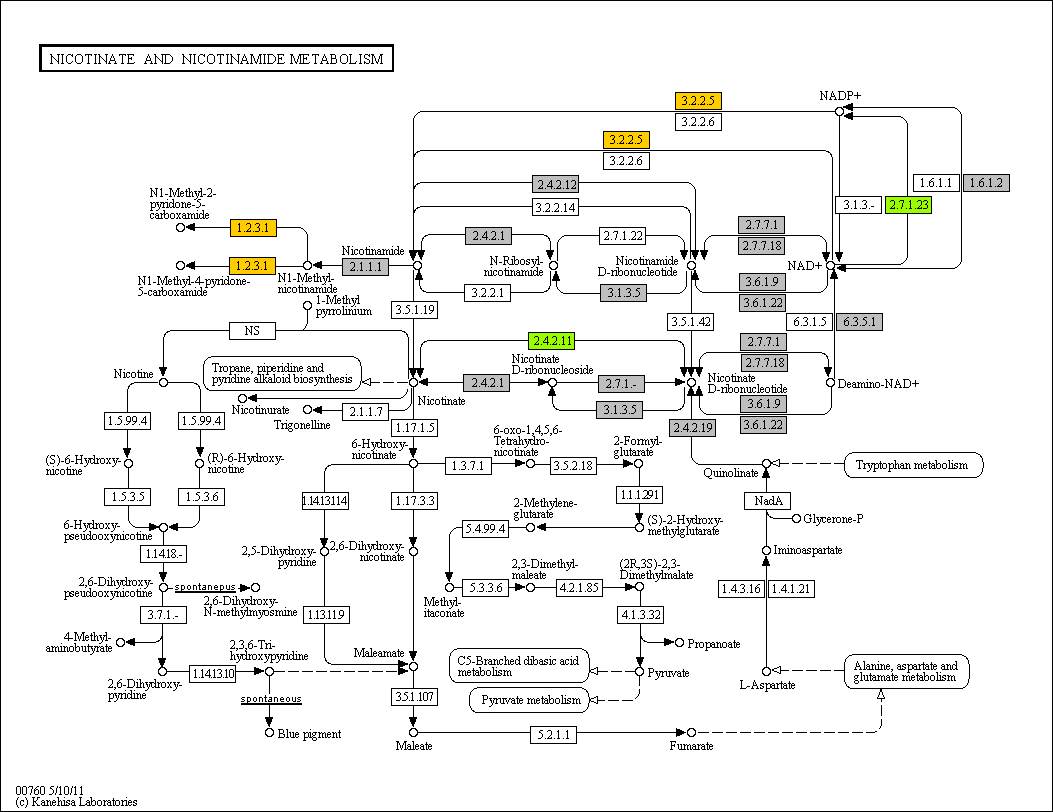


### One carbon pool by folate


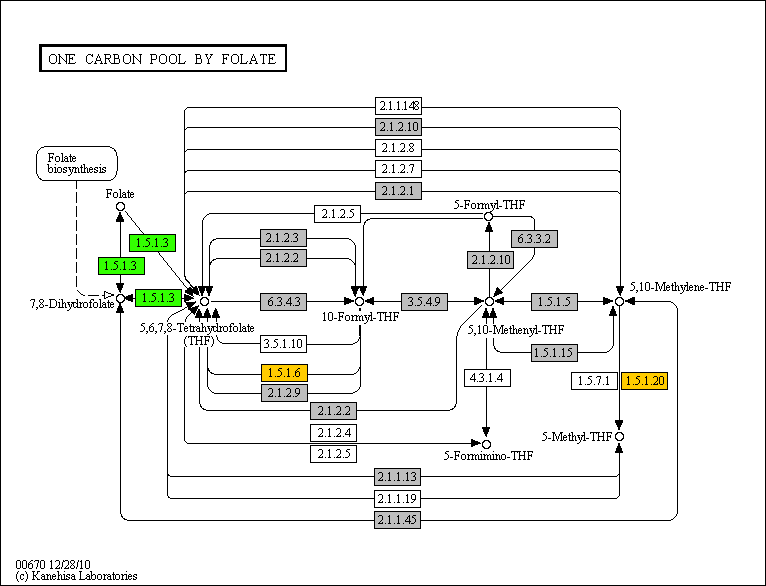


### Pantothenate and CoA biosynthesis


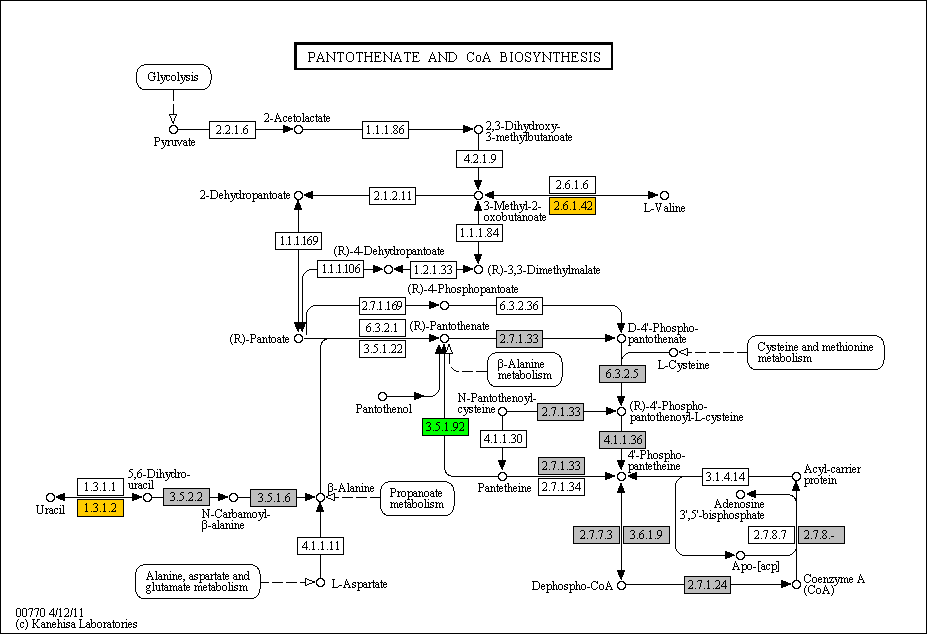


### Porphyrin and chlorophyll metabolism


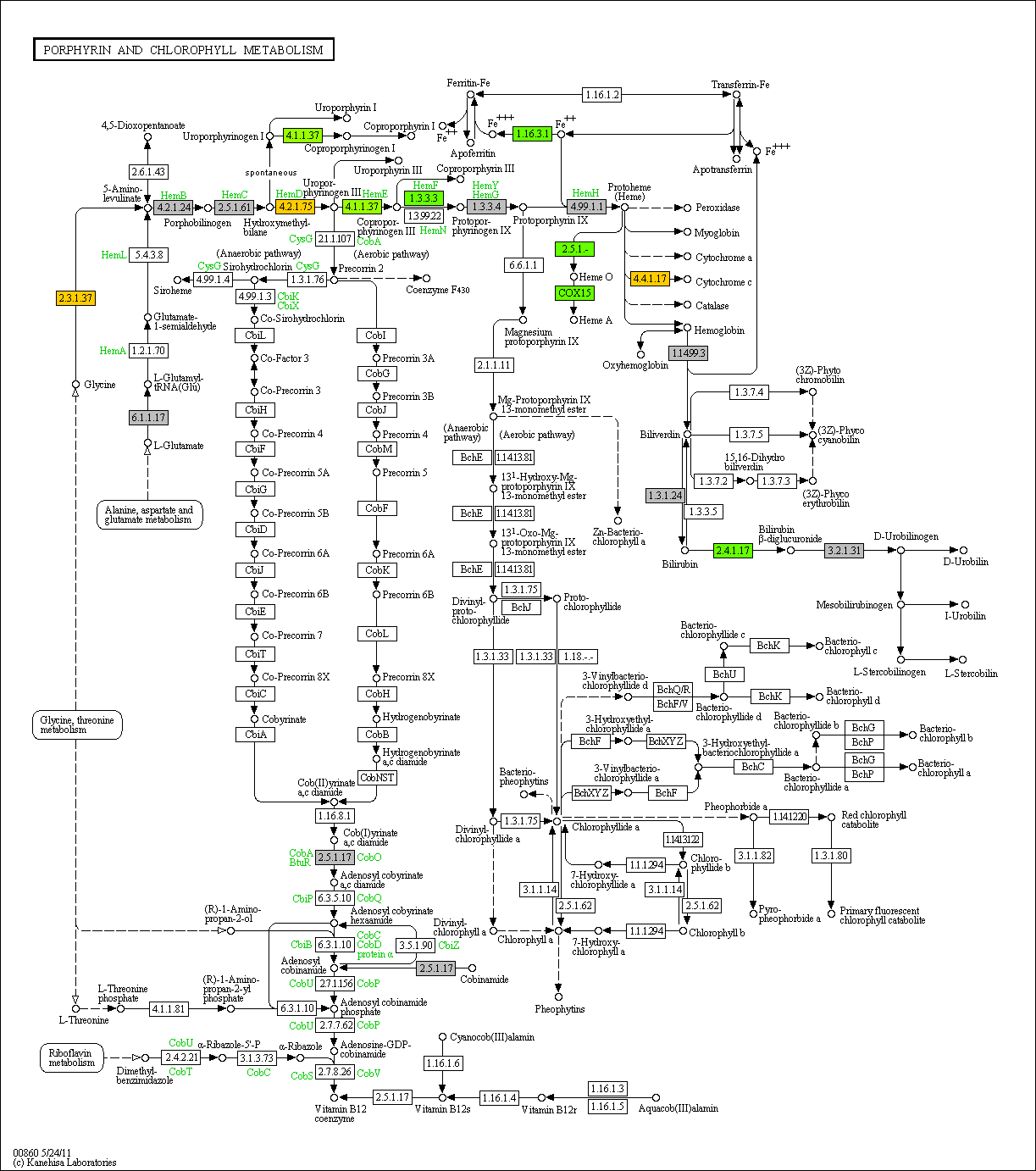


### Retinol metabolism


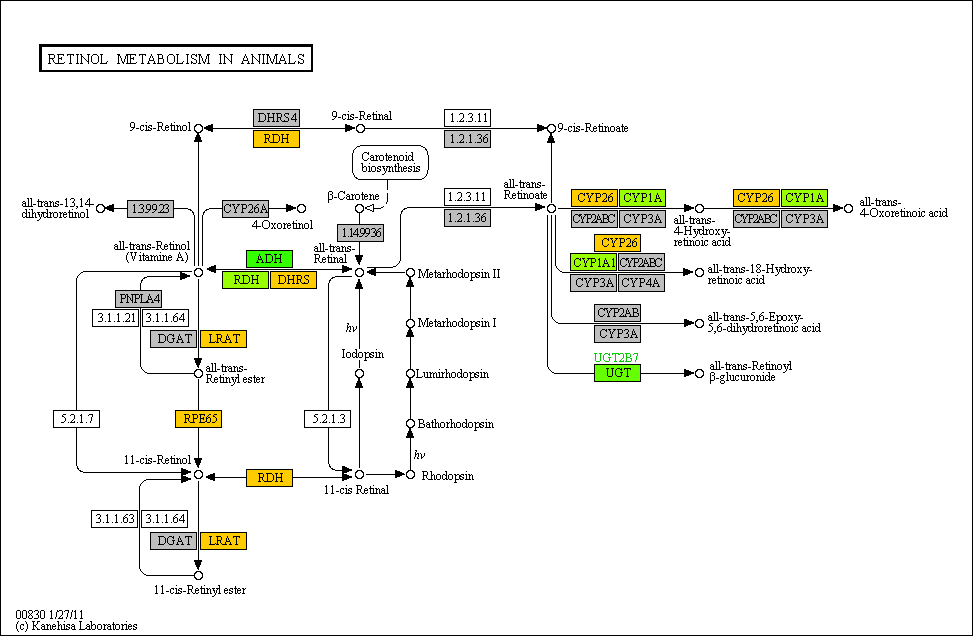


### Riboflavin metabolism


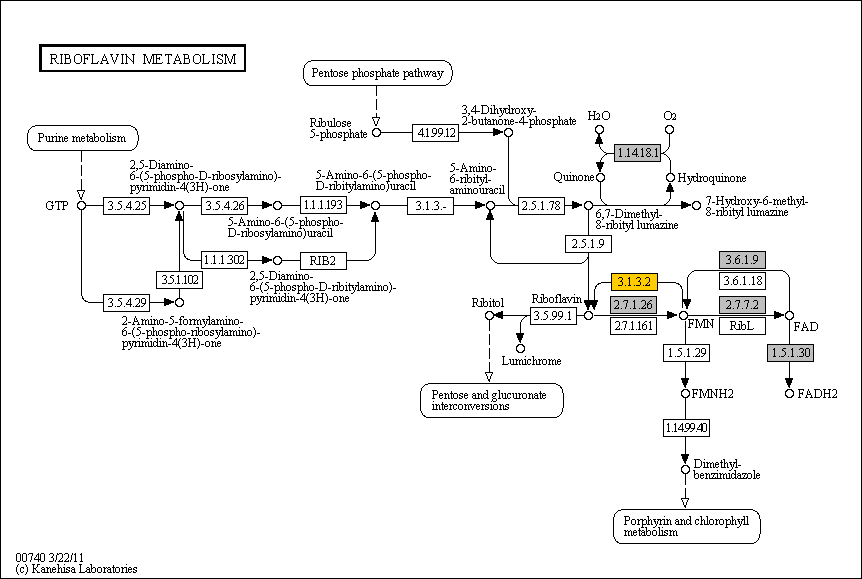


### Thiamine metabolism


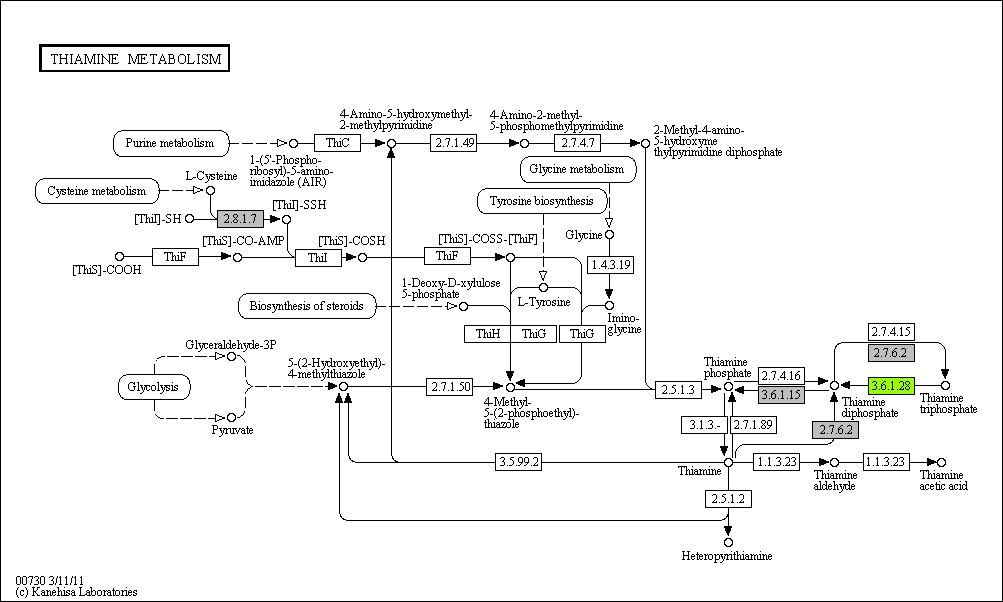


### Vitamin B6 metabolism


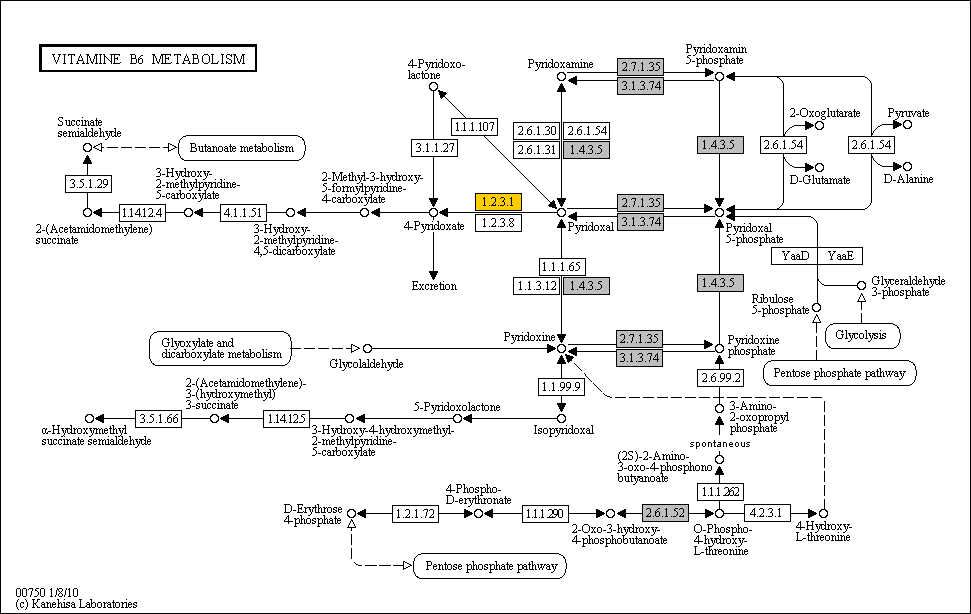


# 1. Metabolism

## 1.9 Metabolism of Terpenoids and Polyketides

### Terpenoid backbone biosynthesis


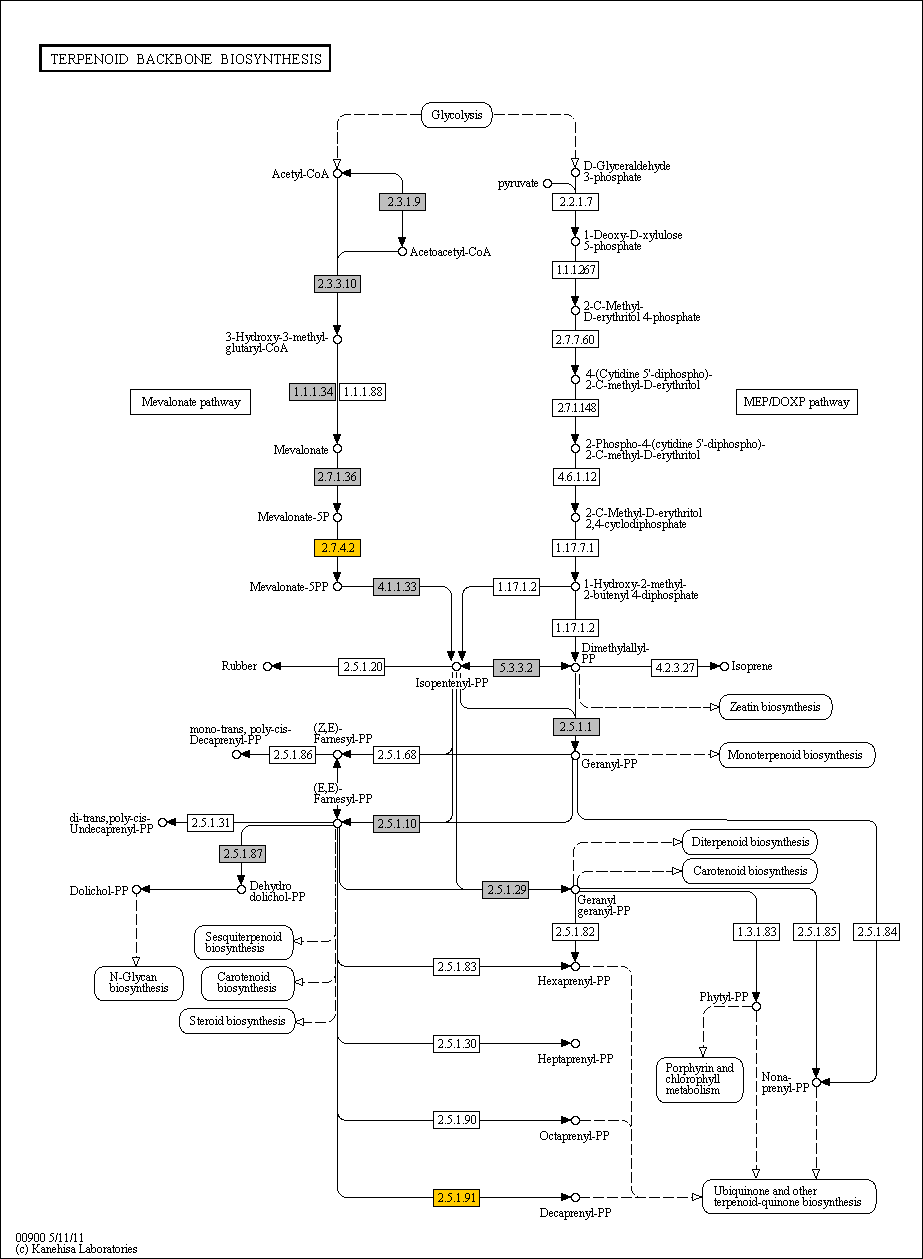


# 1. Metabolism

## 1.10 Biosynthesis of Other Secondary Metabolites

Caffeine metabolism
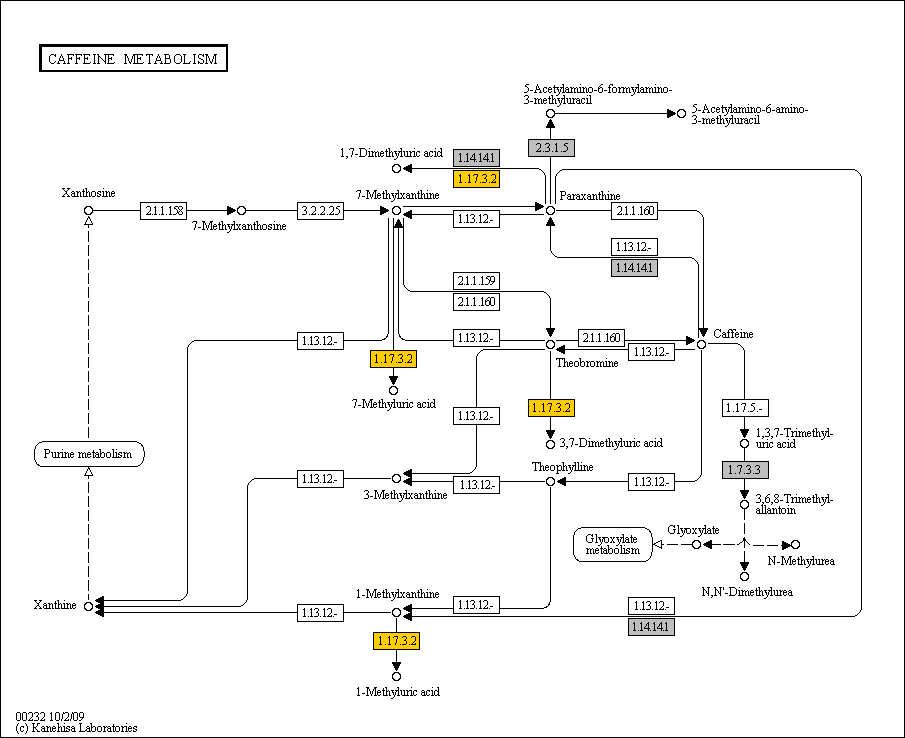


# 1. Metabolism

## 1.11 Xenobiotics Biodegradation and Metabolism

### Drug metabolism - cytochrome P450


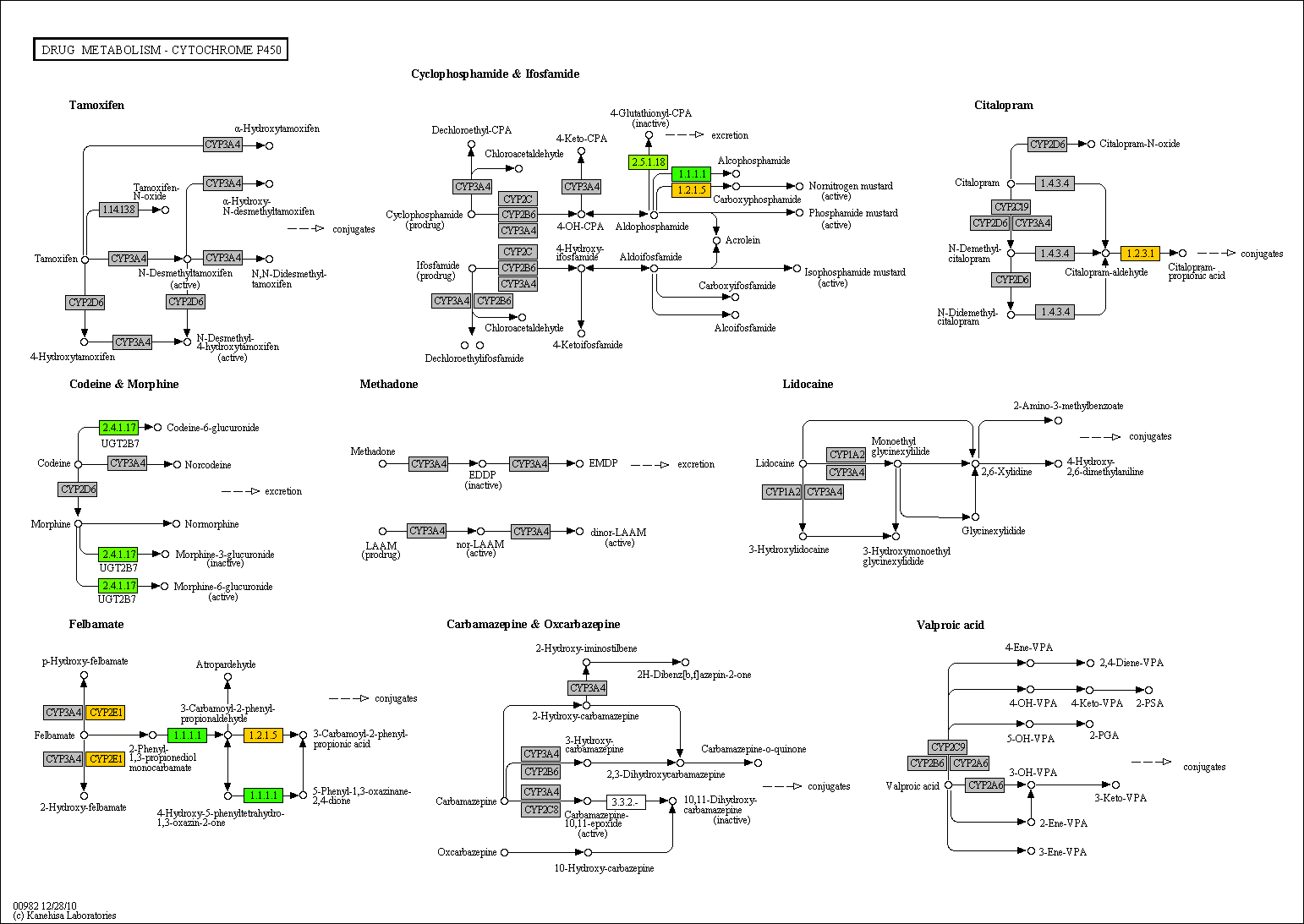


### Drug metabolism - other enzymes


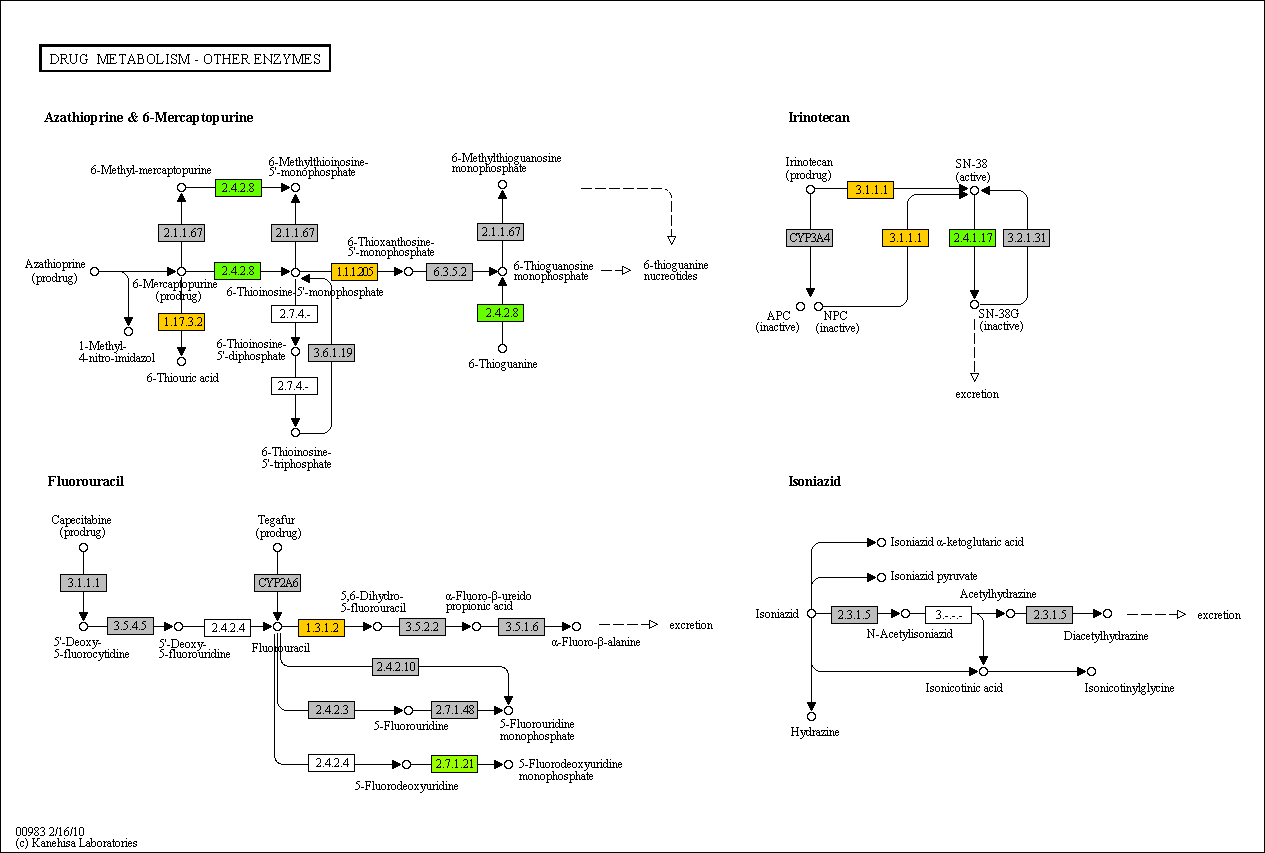


Metabolism of xenobiotics by cytochrome P450
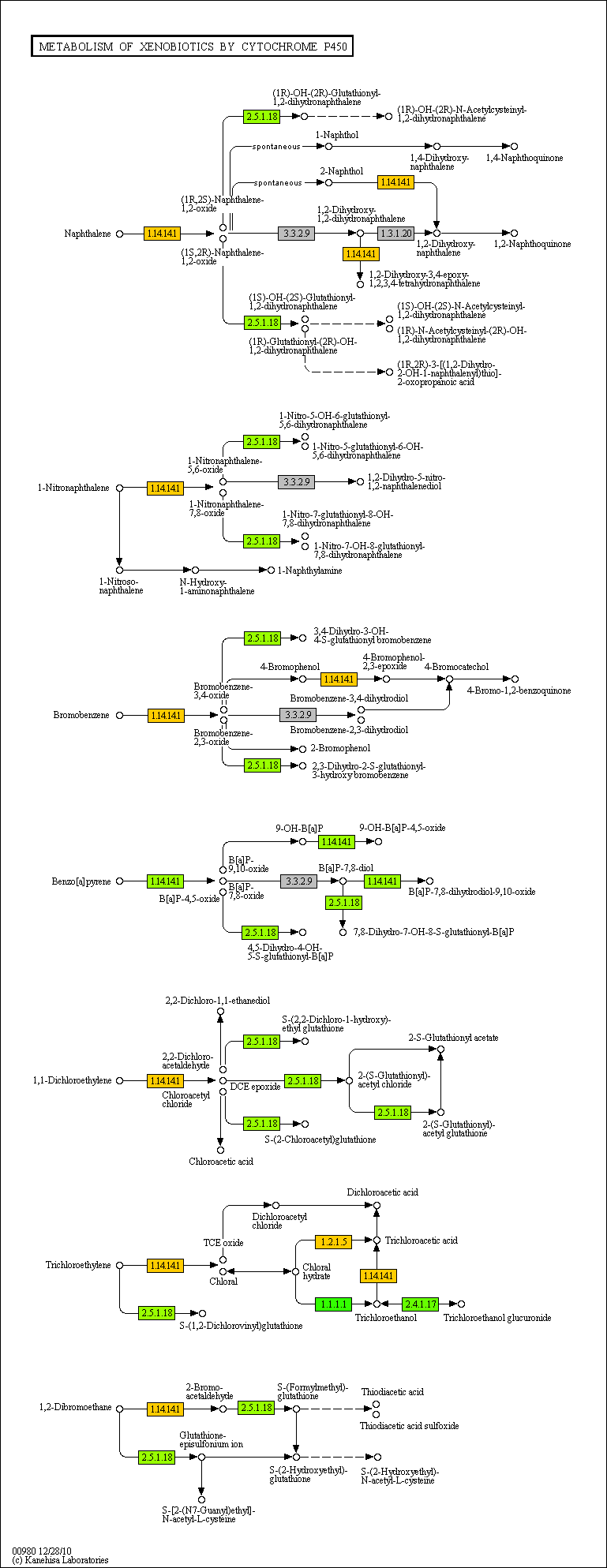


# 2. Genetic Information Processing

## 2.1 Transcription

### Basal transcription factors


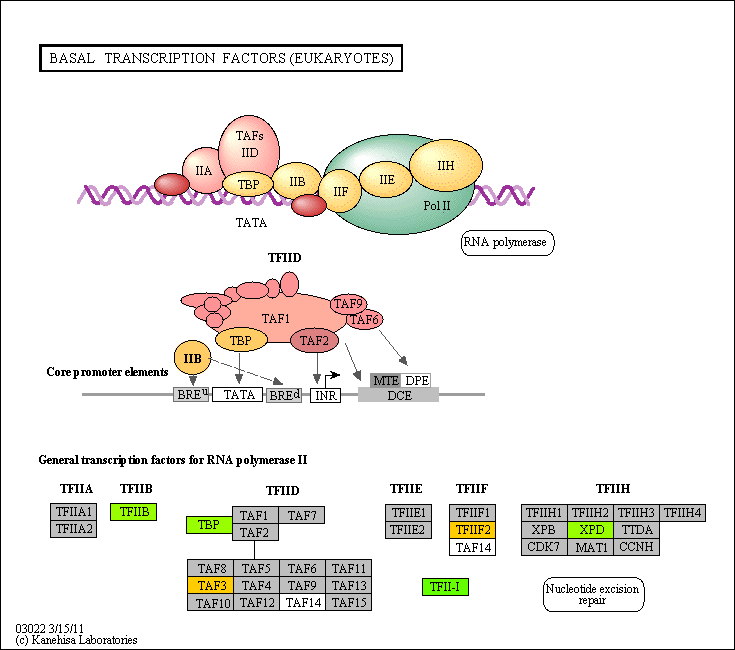


### RNA polymerase


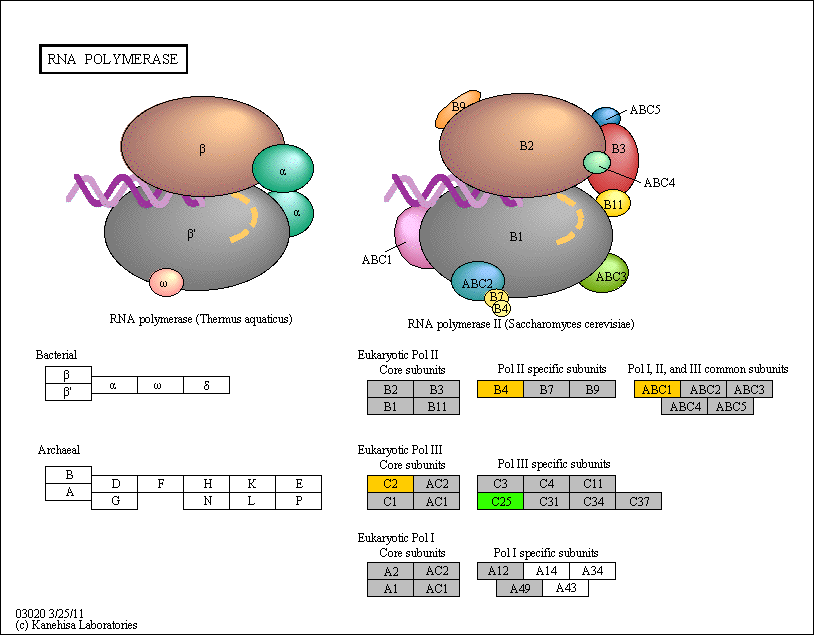


### Spliceosome


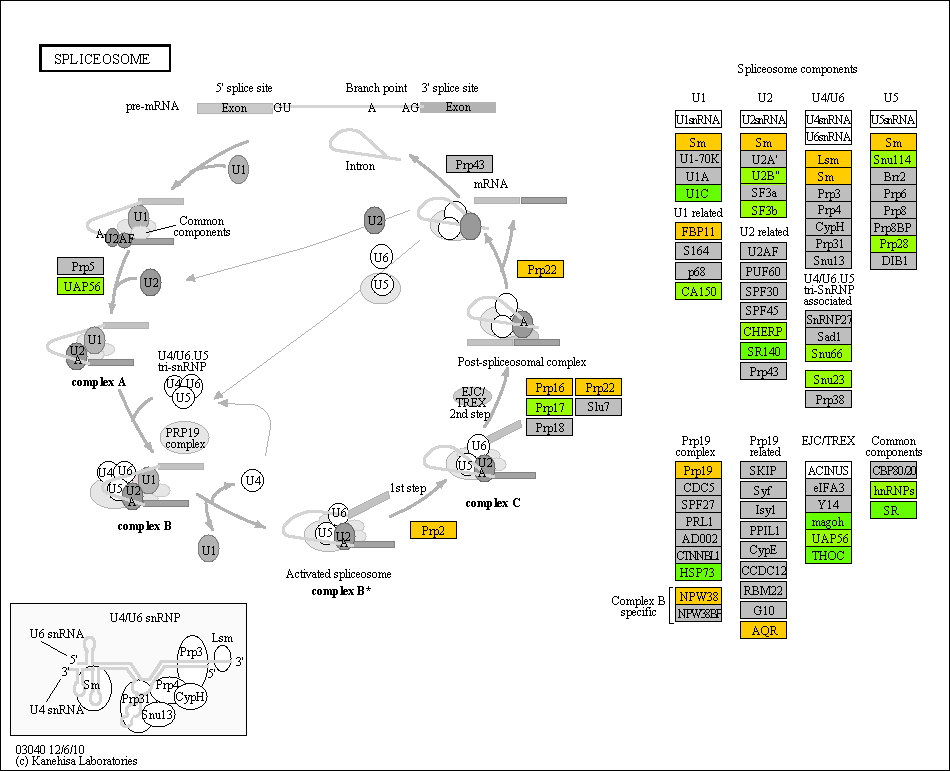


# 2. Genetic Information Processing

## 2.2 Translation

### Aminoacyl-tRNA biosynthesis


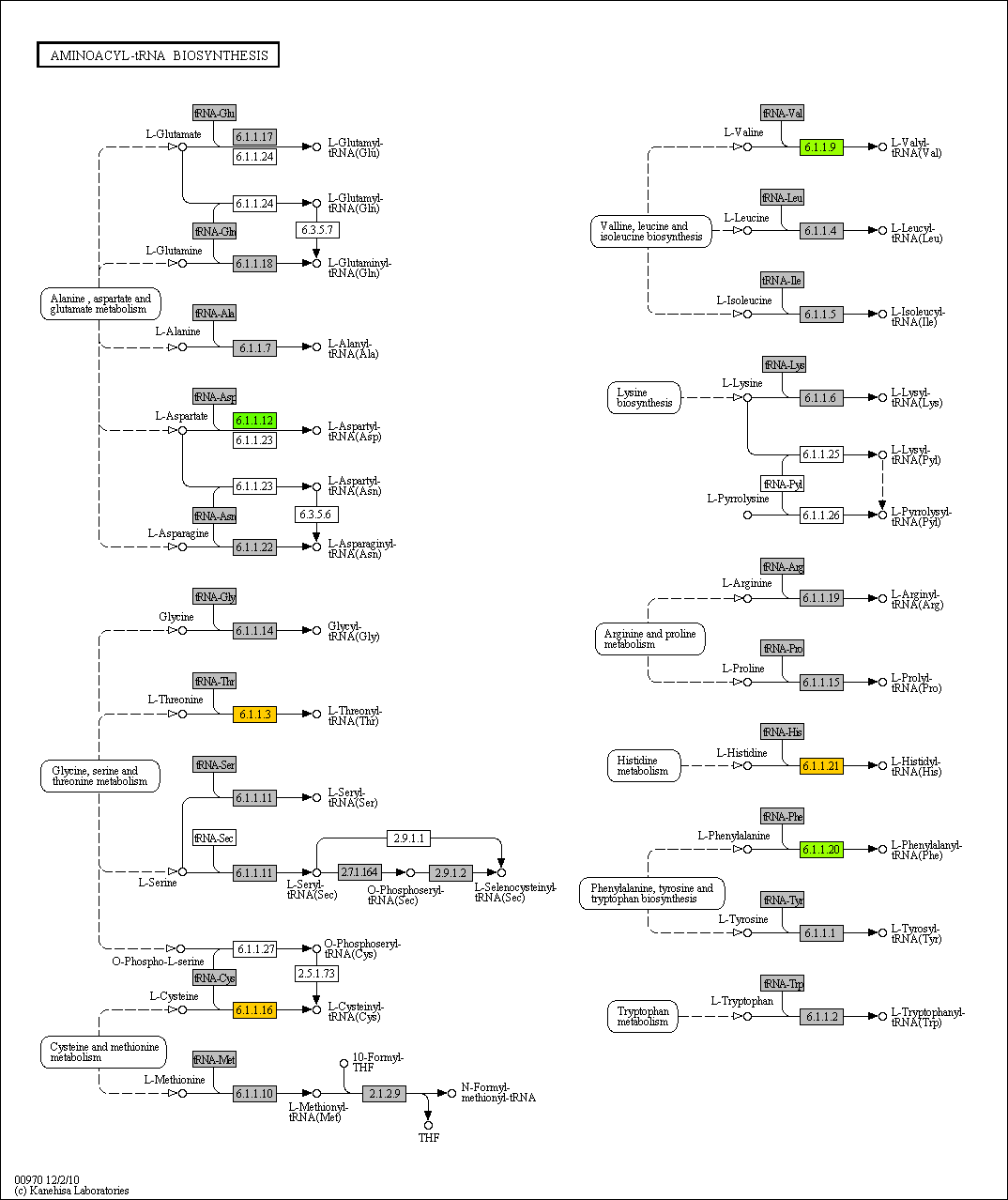


### mRNA surveillance pathway


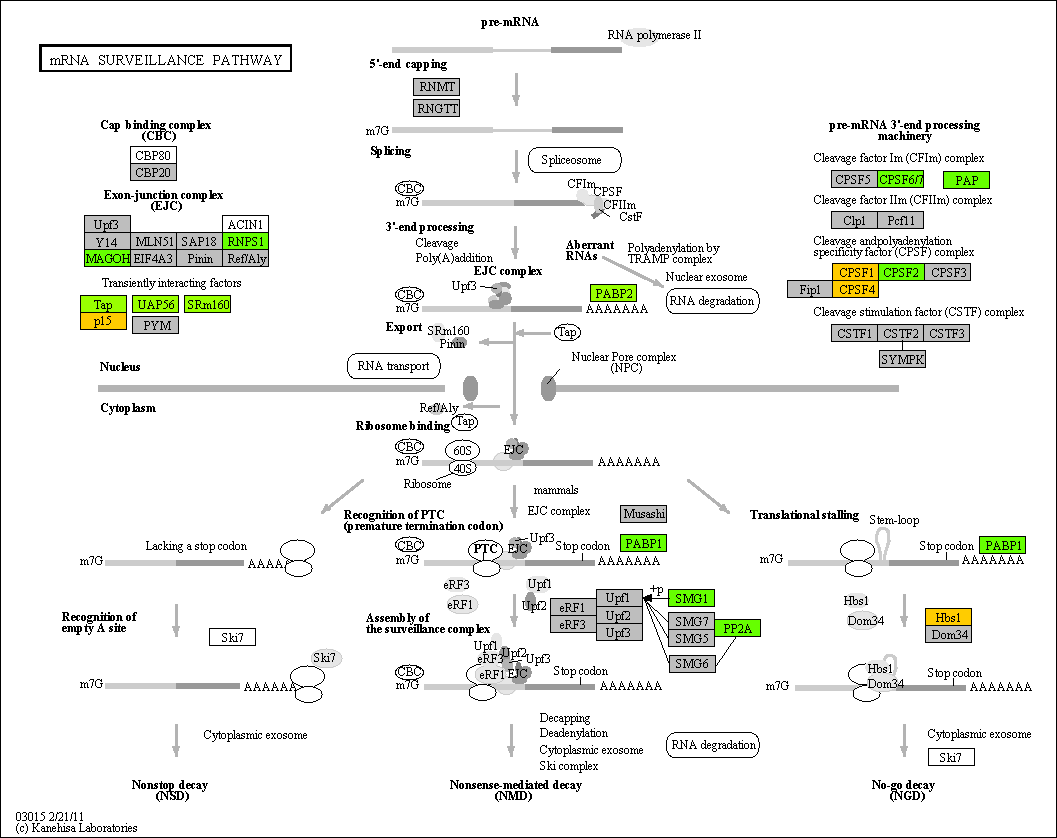


### Ribosome


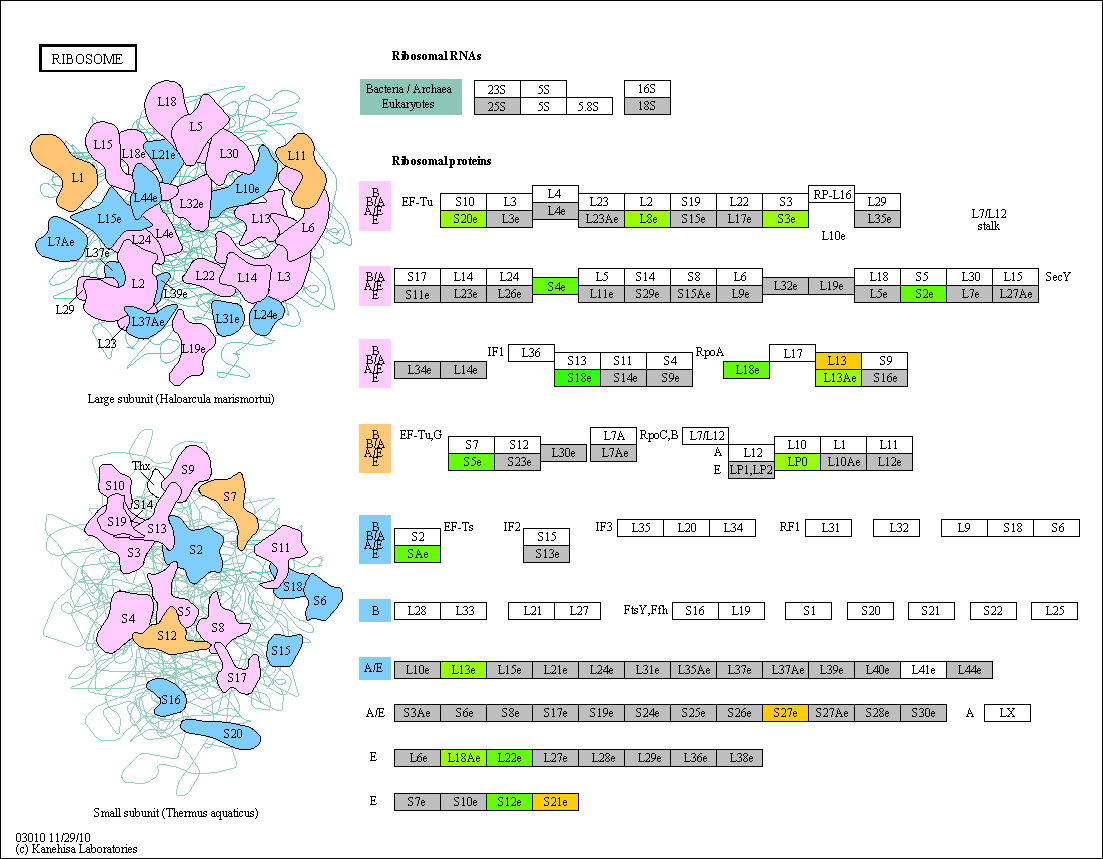


### Ribosome biogenesis in eukaryotes


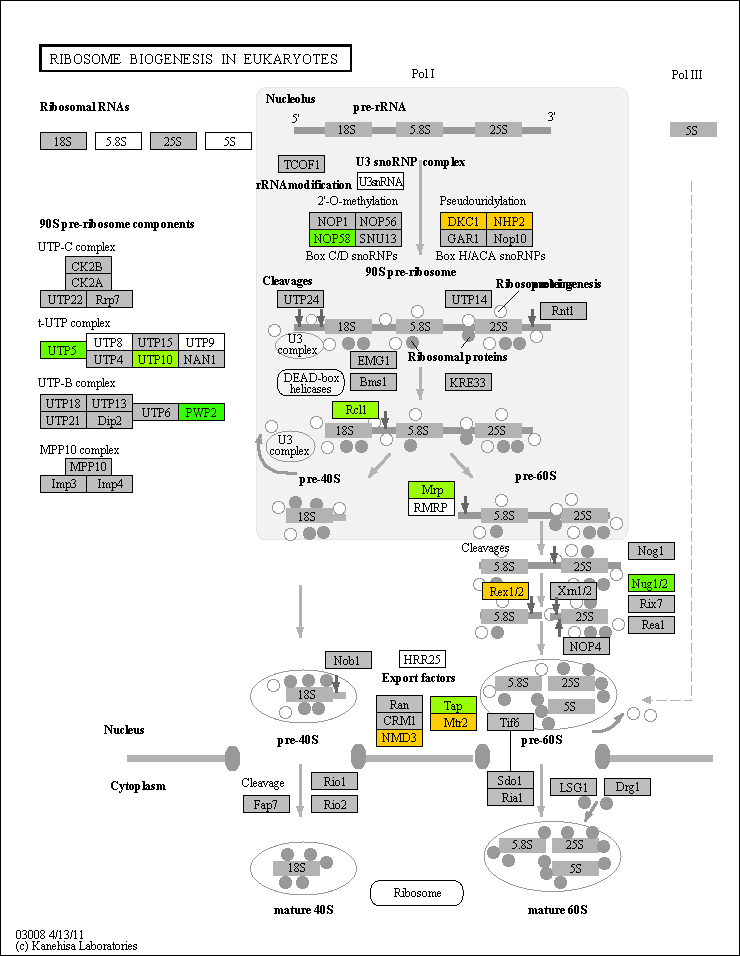


### RNA transport


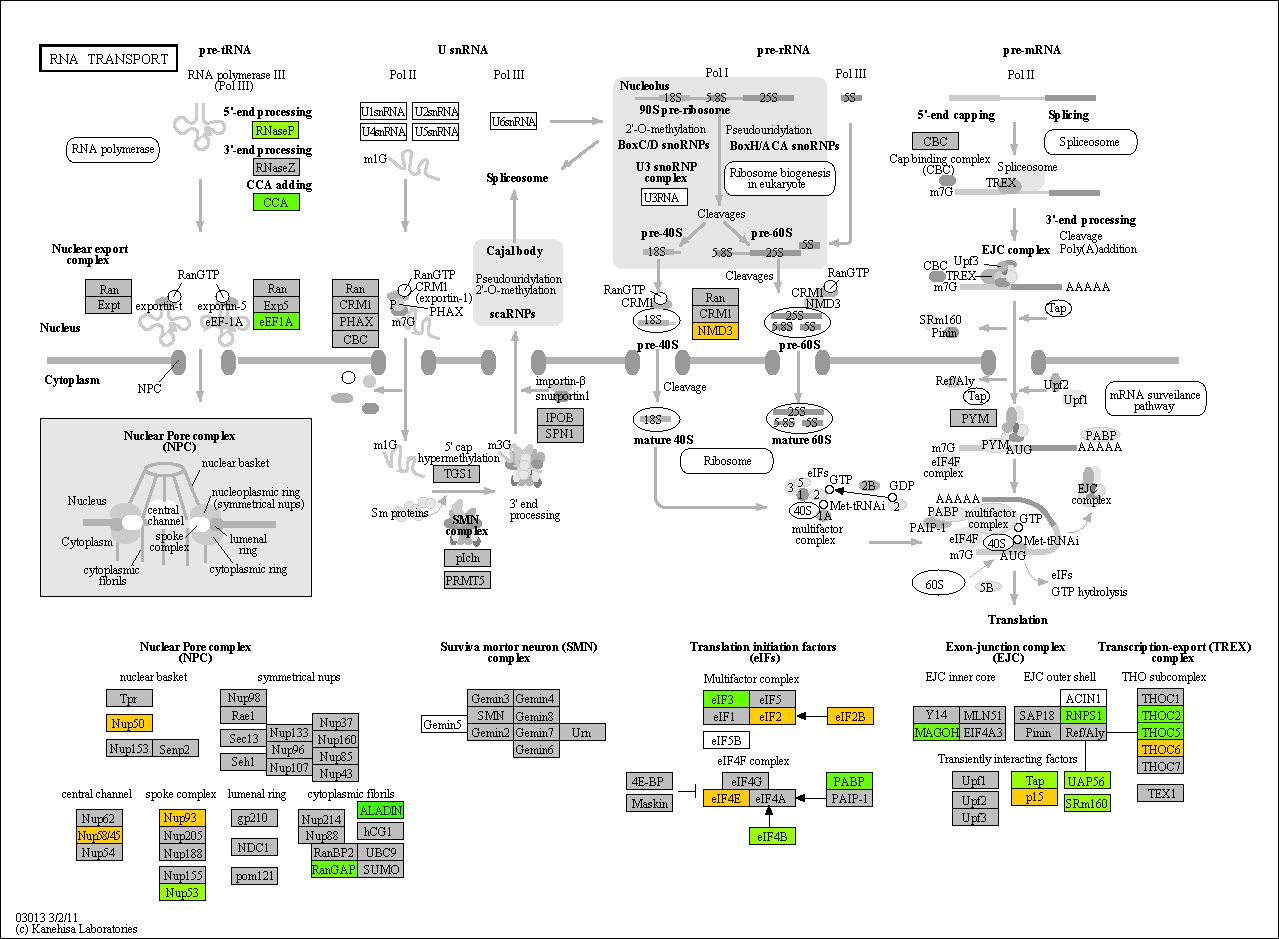


# 2. Genetic Information Processing

## 2.3 Folding, Sorting and Degradation

### Proteasome


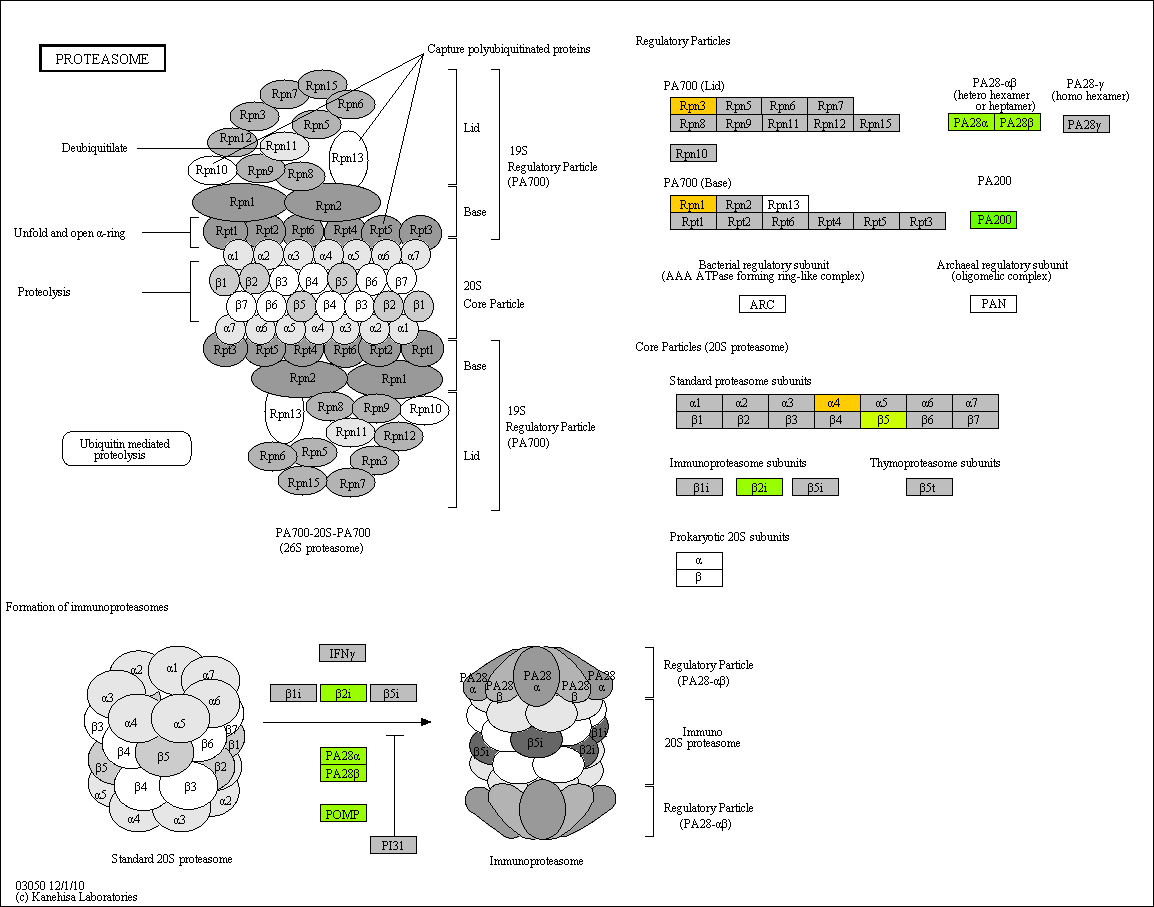


### Protein export


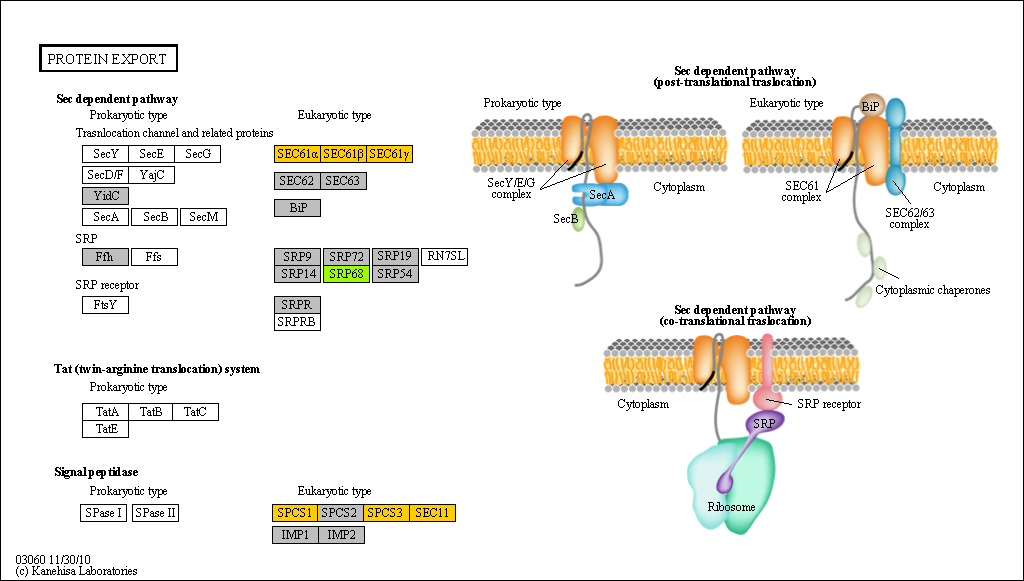


### Protein processing in endoplasmic reticulum


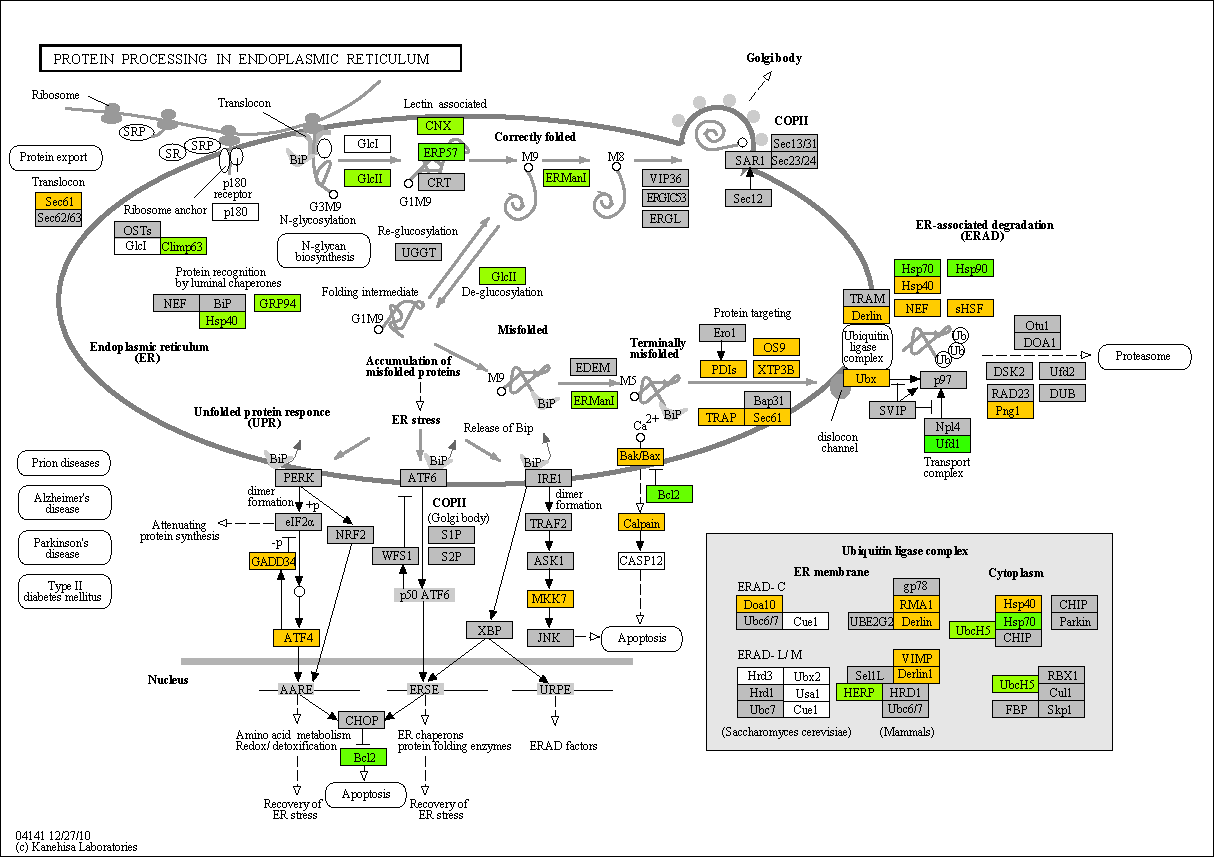


### RNA degradation


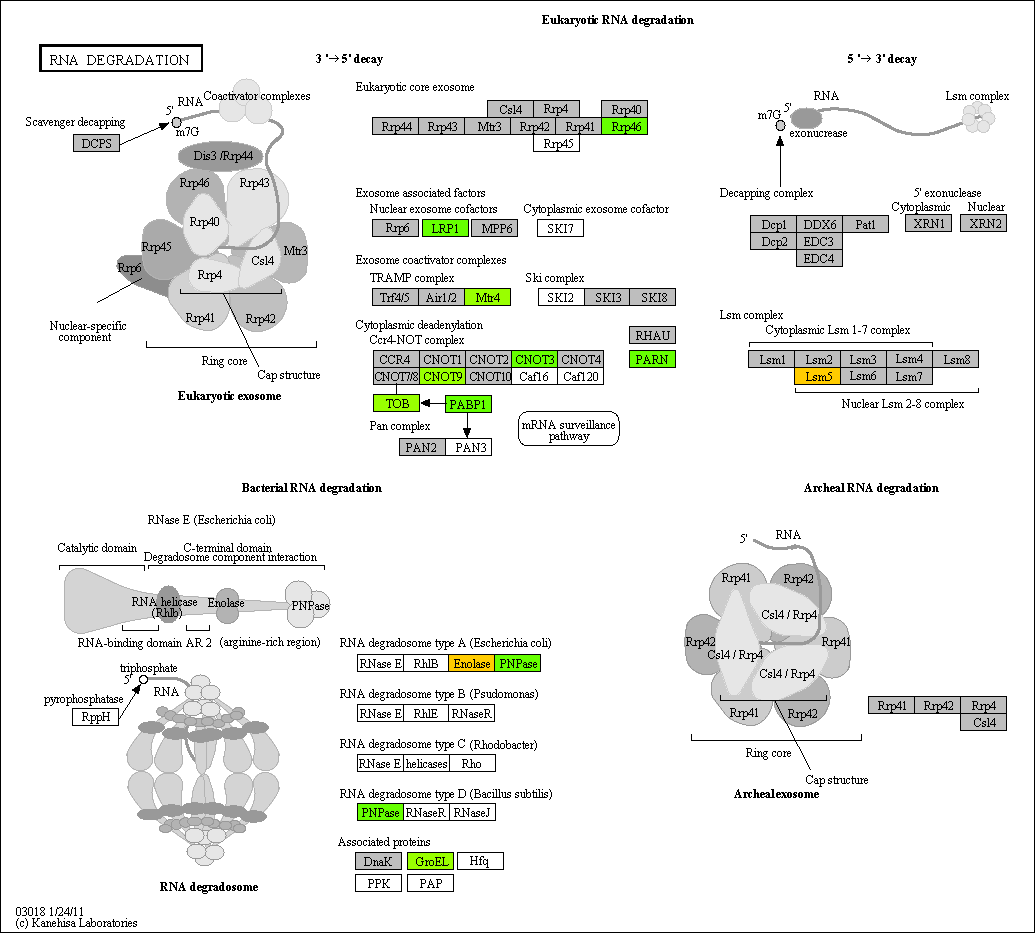


### SNARE interactions in vesicular transport


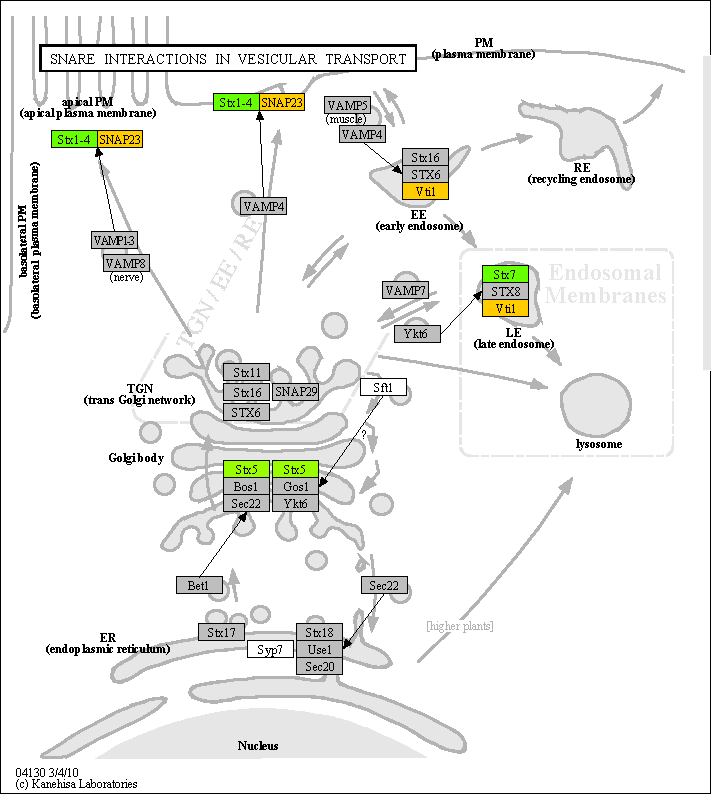


### Sulfur relay system


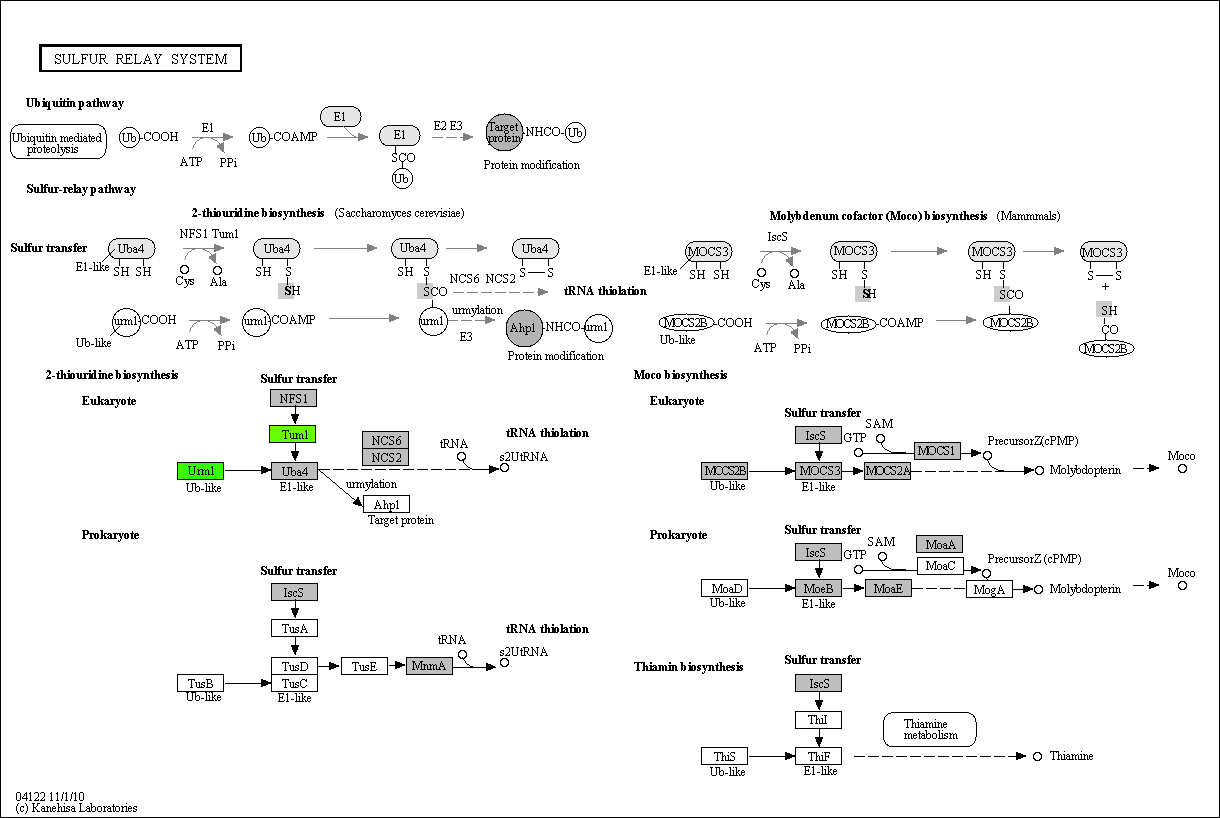


### Ubiquitin mediated proteolysis


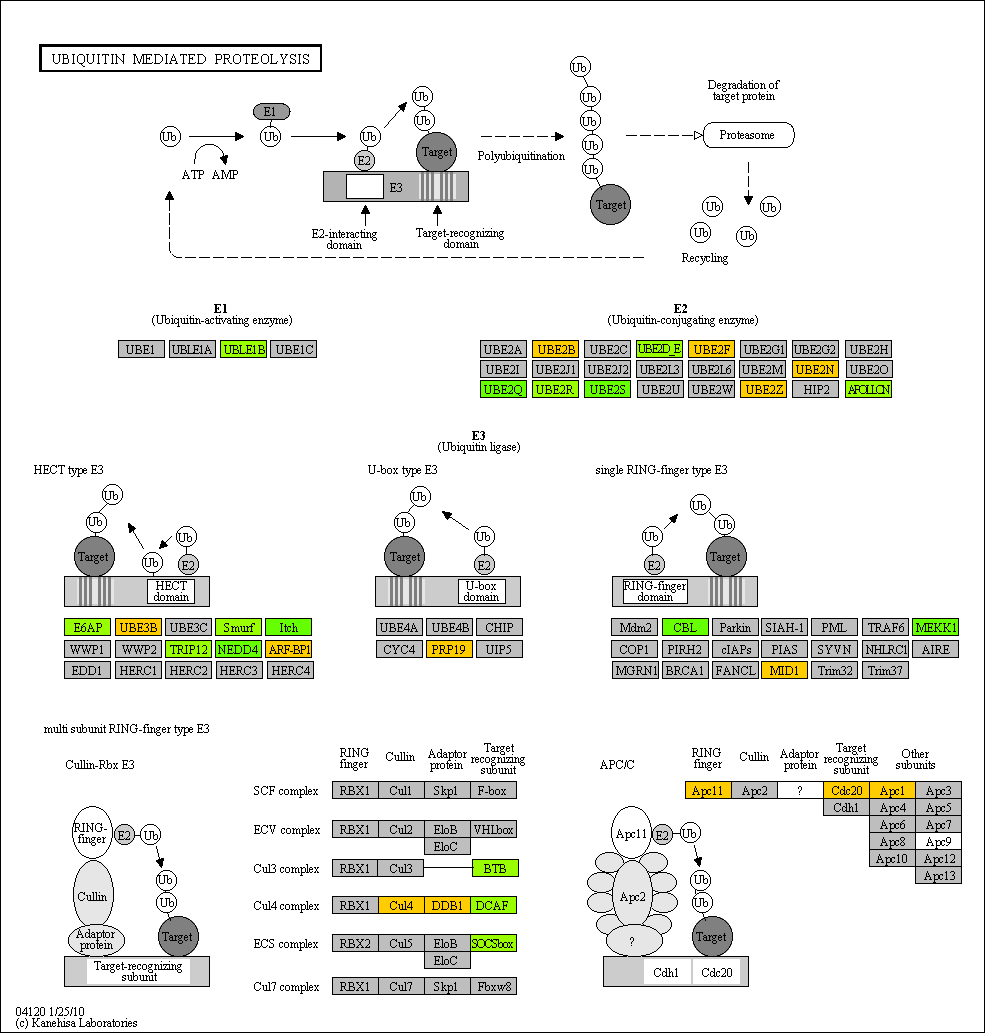


# 2. Genetic Information Processing

## 2.4 Replication and Repair

### Base excision repair


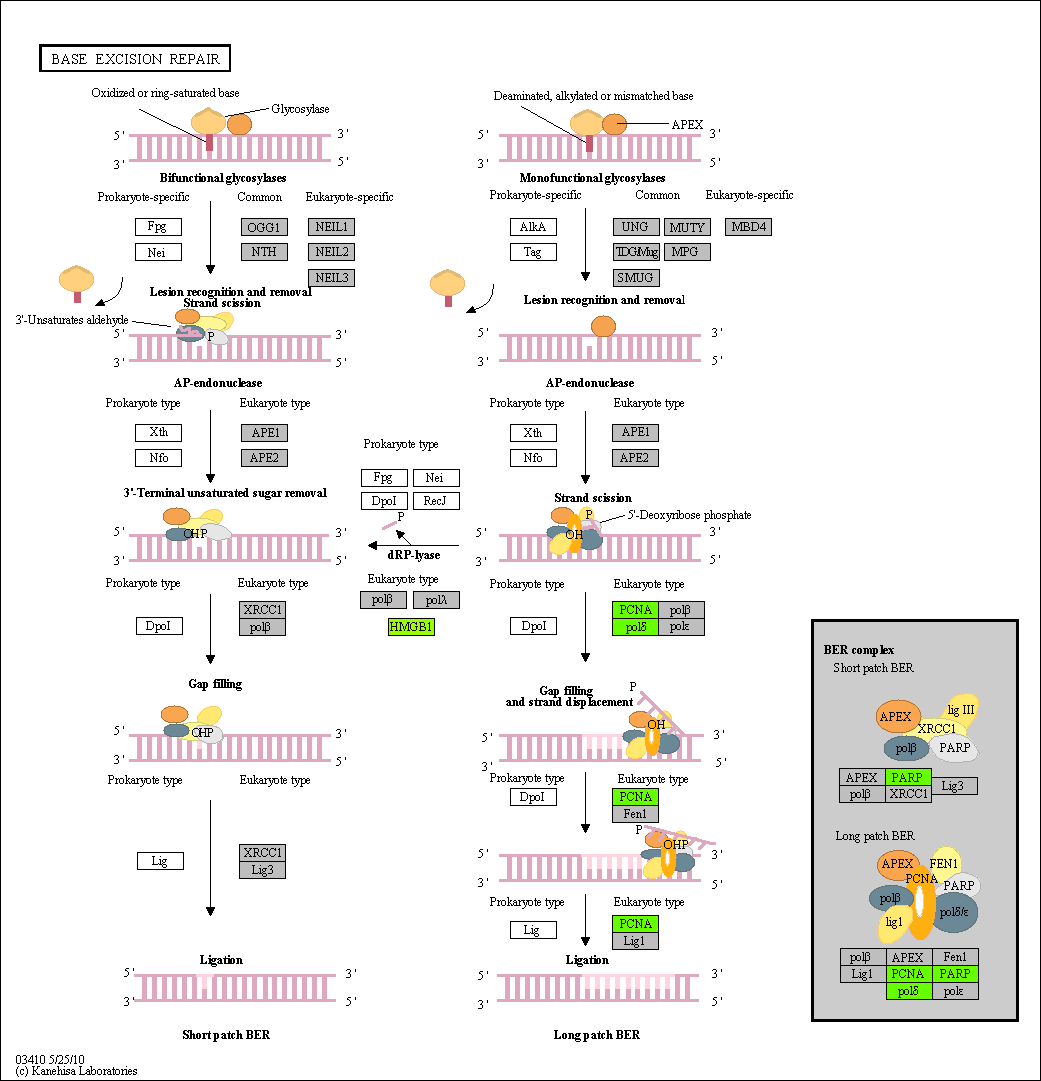


### DNA replication


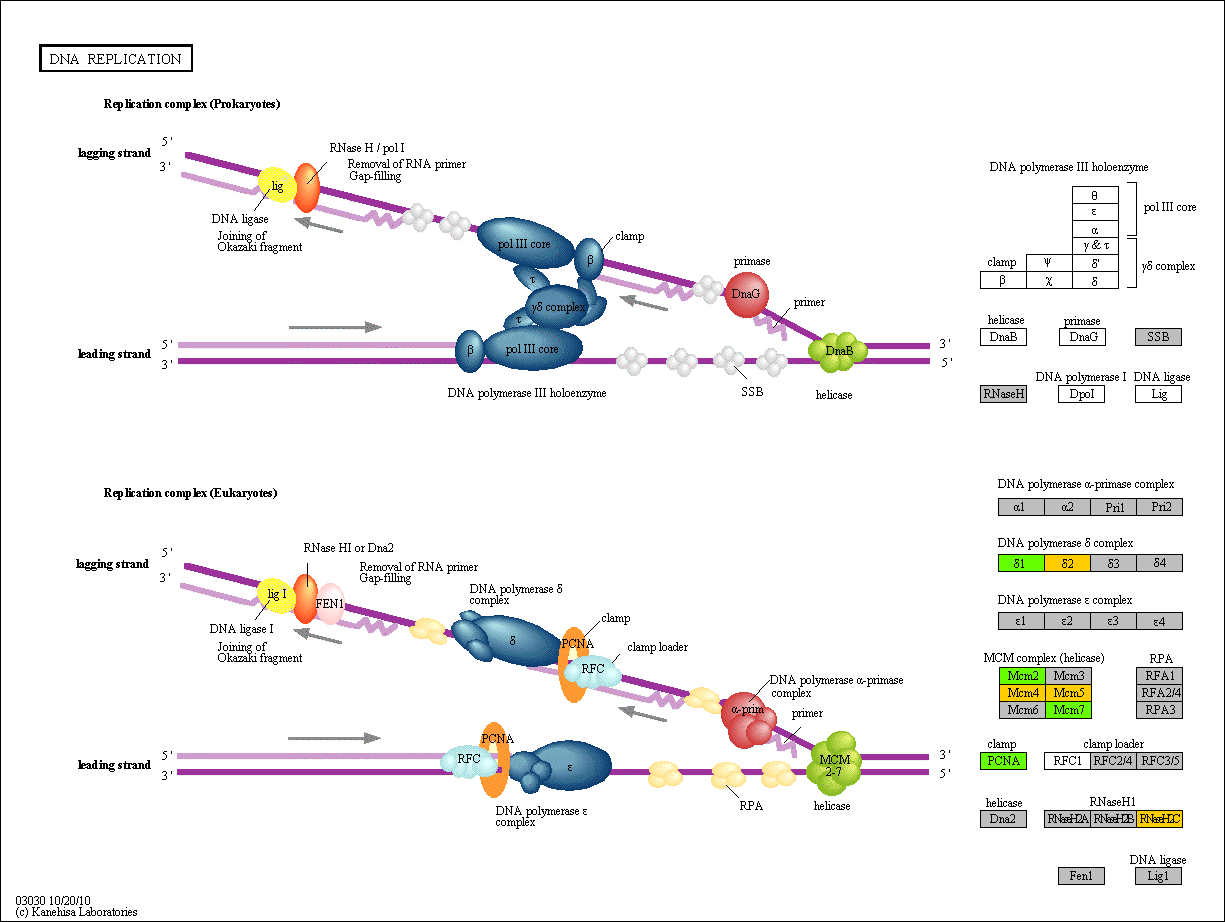


### Homologous recombination


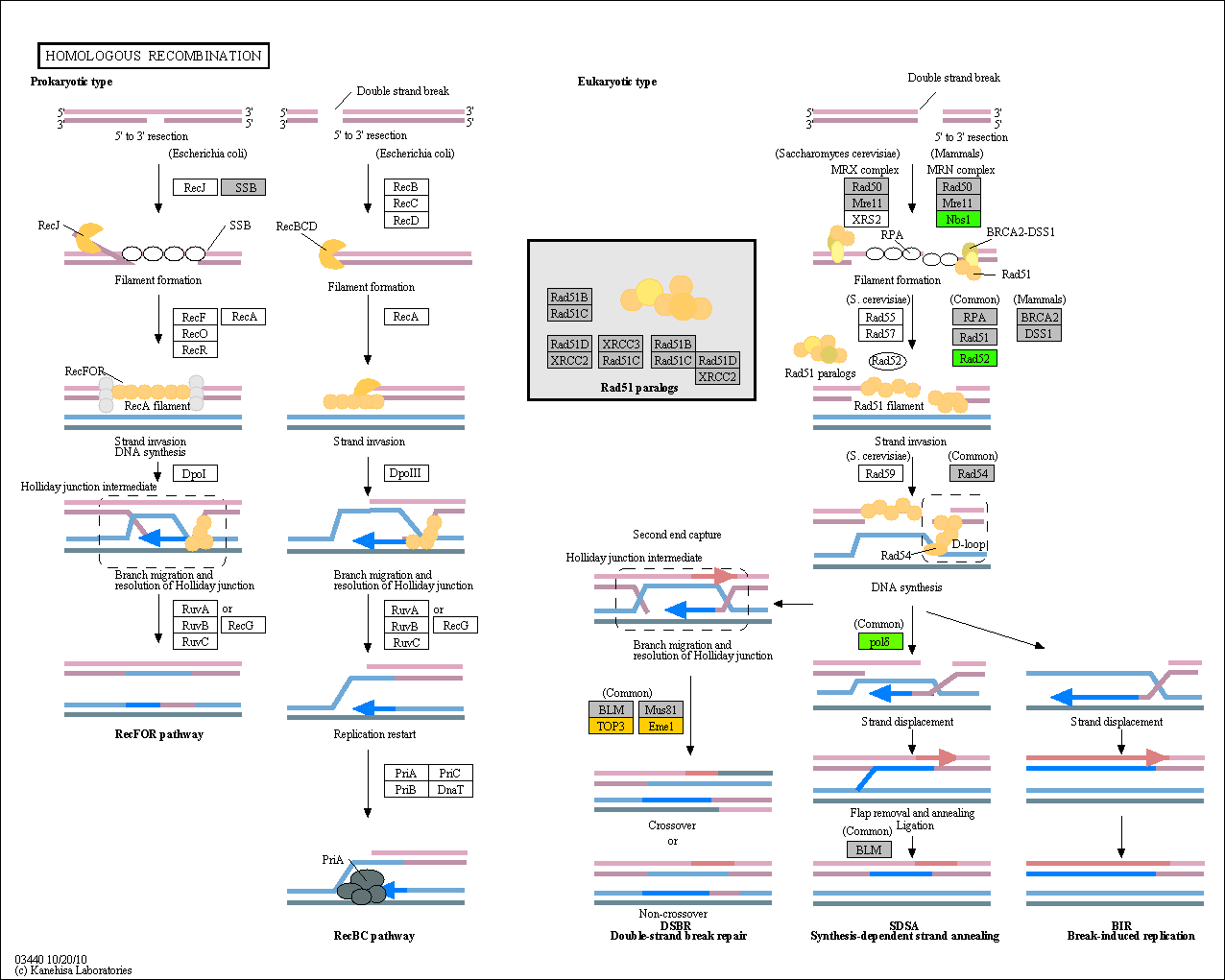


### Mismatch repair


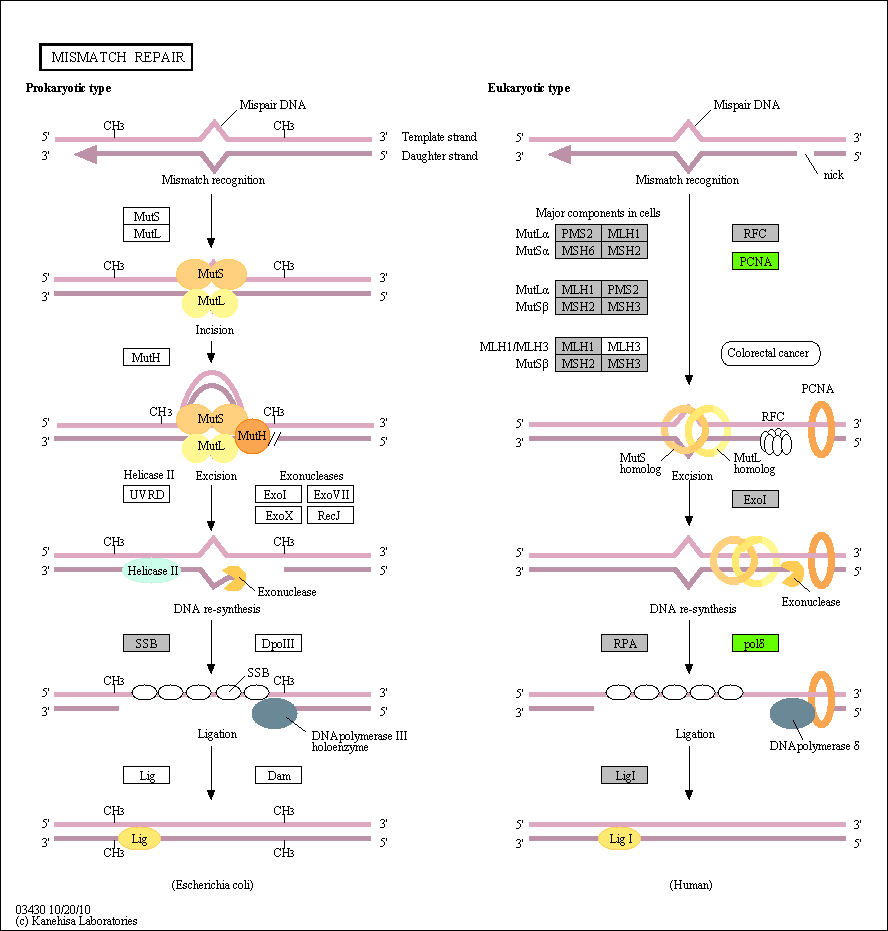


### Non-homologous end-joining


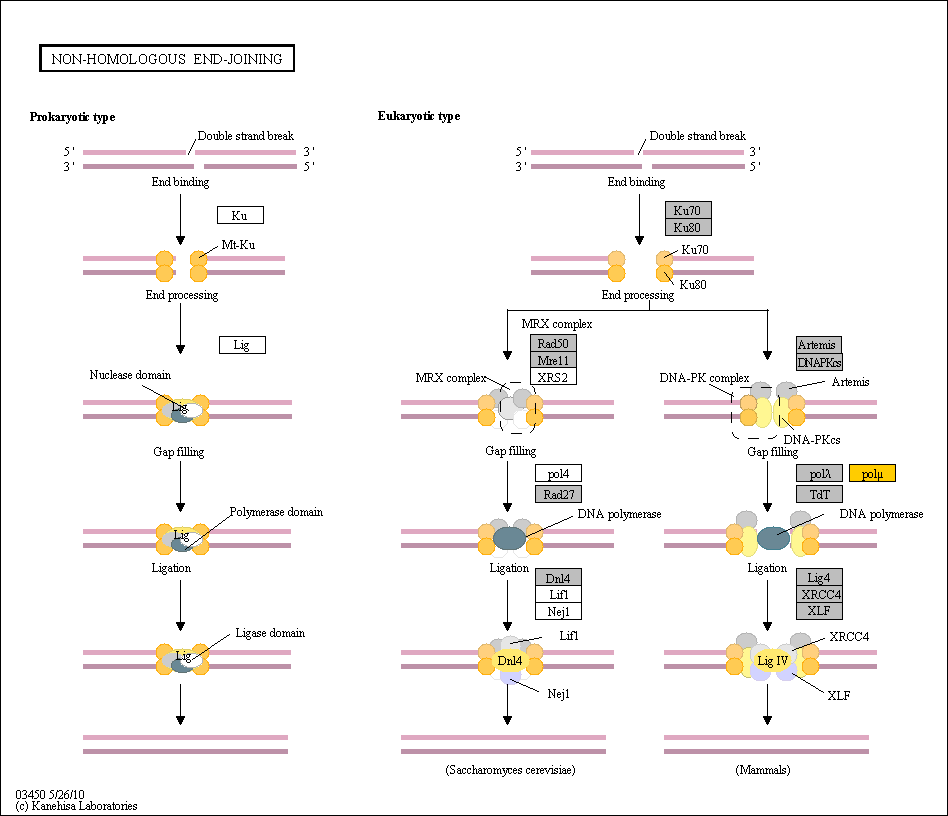


### Nucleotide excision repair


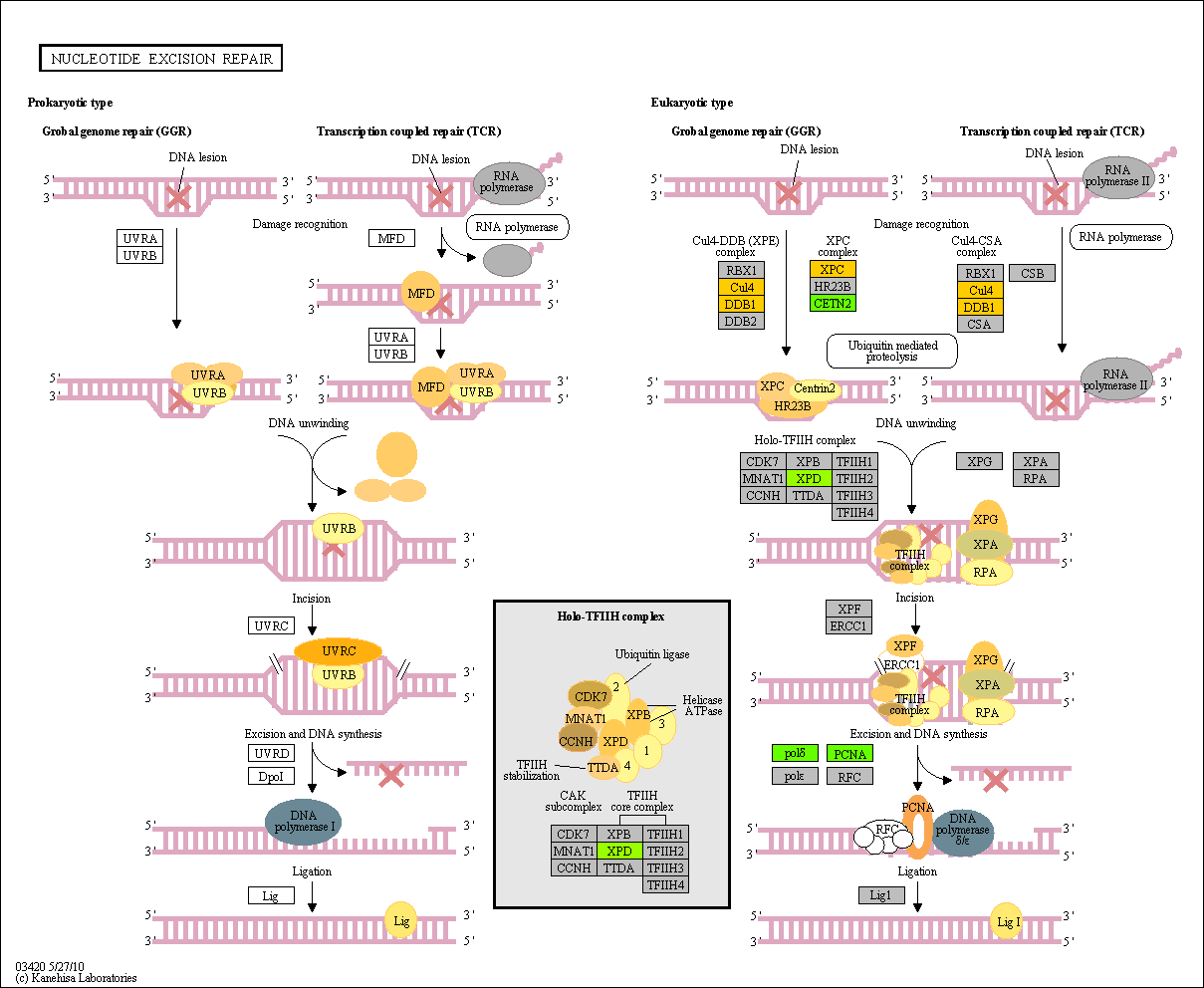


# 3. Environmental Information Processing

## 3.1 Membrane Transport

### ABC transporters


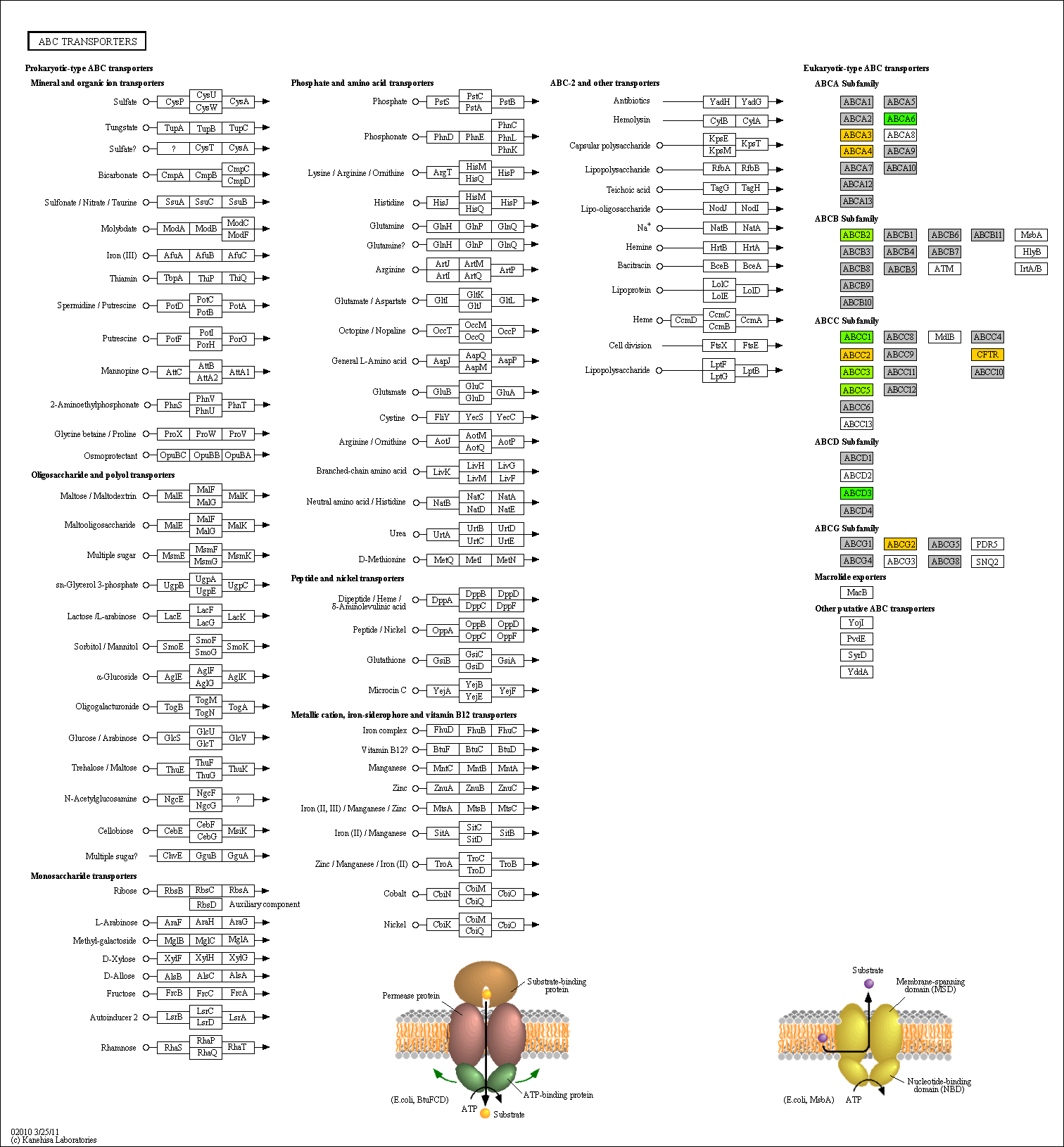


# 3. Environmental Information Processing

### 3.2 Signal Transduction

### Calcium signaling pathway


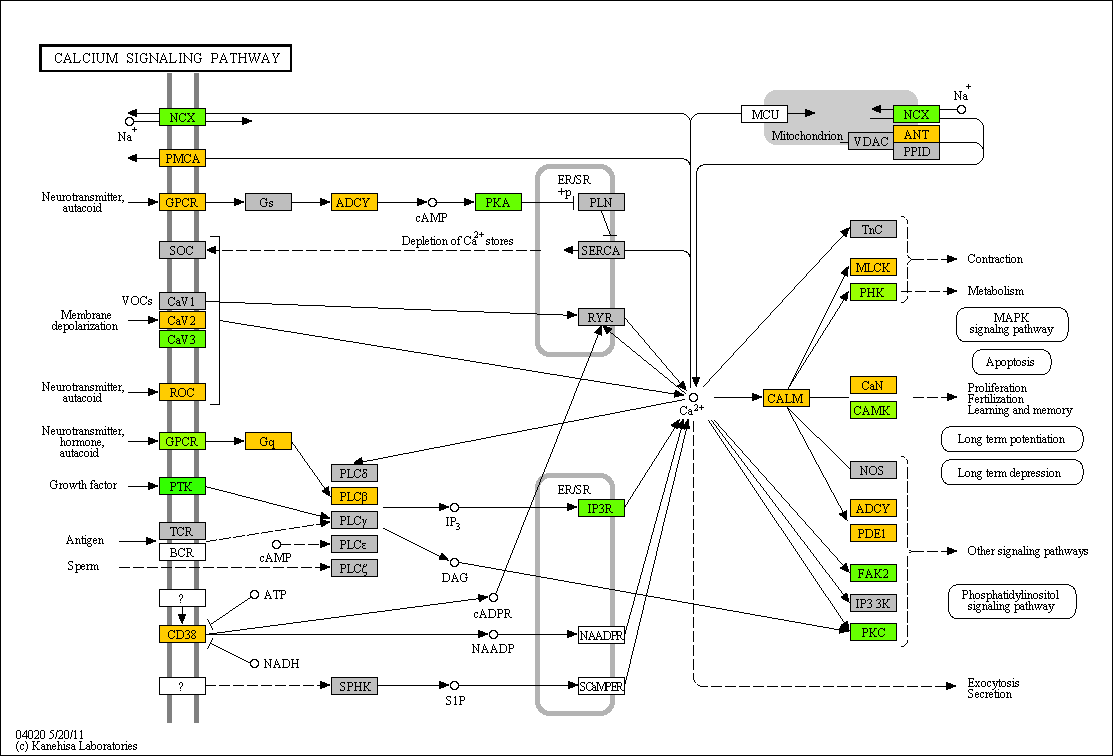


### ErbB signaling pathway


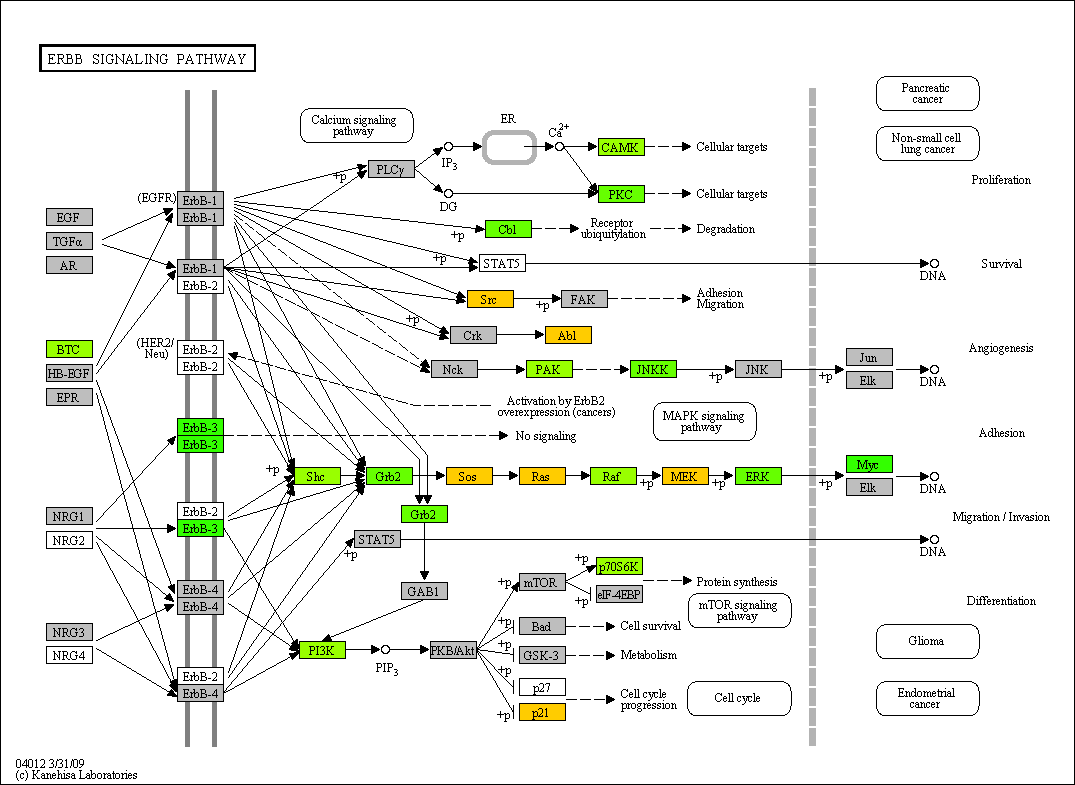


### Hedgehog signaling pathway


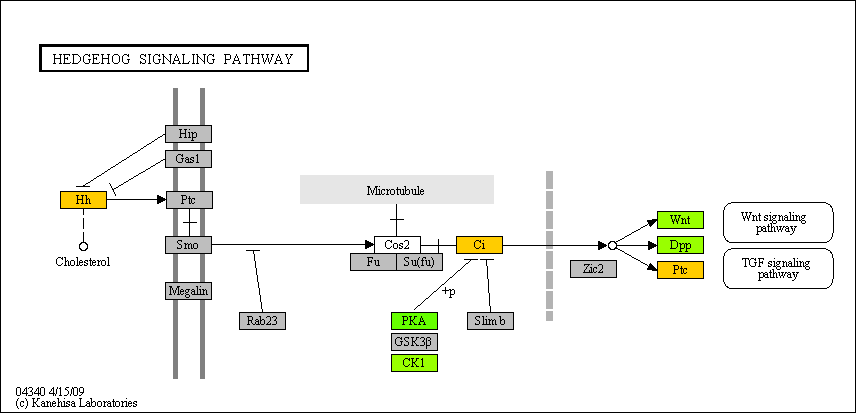


### Jak-STAT signaling pathway


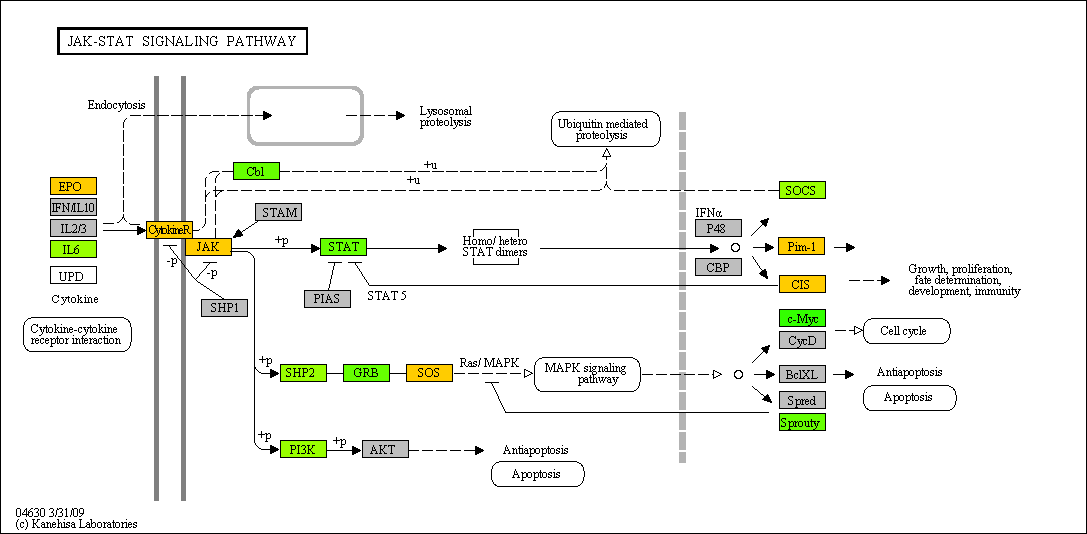


### MAPK signaling pathway

### mTOR signaling pathway

### Notch signaling pathway

### Phosphatidylinositol signaling system

### TGF-beta signaling pathway

### VEGF signaling pathway

### Wnt signaling pathway

# 3. Environmental Information Processing

## 3.3 Signaling Molecules and Interaction

Cell adhesion molecules (CAMs)

Cytokine-cytokine receptor interaction

### ECM-receptor interaction

# 4. Cellular Processes

## 4.1 Transport and Catabolism

### Endocytosis

### Lysosome

### Peroxisome

### Phagosome

### Regulation of autophagy

# 4. Cellular Processes

## 4.2 Cell Motility

### Regulation of actin cytoskeleton

# 4. Cellular Processes

## 4.3 Cell Growth and Death

### Apoptosis

### Cell cycle

### Oocyte meiosis

### p53 signaling pathway

# 4. Cellular Processes

## 4.4 Cell Communication

### Adherens junction

### Focal adhesion

### Gap junction

### Tight junction

# 5. Organismal Systems

## 5.1 Immune System

### Antigen processing and presentation

### B cell receptor signaling pathway

### Chemokine signaling pathway

### Complement and coagulation cascades

### Cytosolic DNA-sensing pathway

### Fc epsilon RI signaling pathway

### Fc gamma R-mediated phagocytosis

### Hematopoietic cell lineage

### Intestinal immune network for IgA production (the pathway shows many genes up-regulated, but in reality most of those genes are actually down-regulated. This was a problem with KEGG array that was not possible to solve)

### Leukocyte transendothelial migration

### Natural killer cell mediated cytotoxicity

### NOD-like receptor signaling pathway

### RIG-I-like receptor signaling pathway

### T cell receptor signaling pathway

### Toll-like receptor signaling pathway

# 5. Organismal Systems

## 5.2 Endocrine System

### Adipocytokine signaling pathway

### GnRH signaling pathway

### Insulin signaling pathway

### Melanogenesis

### PPAR signaling pathway

### Progesterone-mediated oocyte maturation

### Renin-angiotensin system

# 5. Organismal Systems

## 5.3 Circulatory System

### Cardiac muscle contraction

### Vascular smooth muscle contraction

# 5. Organismal Systems

## 5.4 Digestive System

### Bile secretion

### Carbohydrate digestion and absorption

### Fat digestion and absorption

### Gastric acid secretion

### Mineral absorption

### Pancreatic secretion

### Protein digestion and absorption

### Salivary secretion

### Vitamin digestion and absorption

# 5. Organismal Systems

## 5.5 Excretory System

### Aldosterone-regulated sodium reabsorption

### Collecting duct acid secretion

### Endocrine and other factor-regulated calcium reabsorption

### Proximal tubule bicarbonate reclamation

### Vasopressin-regulated water reabsorption

# 5. Organismal Systems

## 5.6 Nervous System

### Glutamatergic synapse

### Long-term depression

### Long-term potentiation

### Neurotrophin signaling pathway

# 5. Organismal Systems

## 5.7 Sensory System

### Phototransduction

# 5. Organismal Systems

## 5.8 Development

### Axon guidance

### Dorso-ventral axis formation

### Osteoclast differentiation

# 5. Organismal Systems

## 5.9 Environmental Adaptation

### Circadian rhythm - mammal
