# Supplementary material for: Old and New Stories: Revelations from Functional Analysis of the Bovine Mammary Transcriptome during the Lactation Cycle
Source: PLoS One. 2012 Mar 12;7(3):e33268. doi: 10.1371/journal.pone.0033268 (PMC3299771; doi:10.1371/journal.pone.0033268)
Supplement: File S3 — Additional Materials & Methods, results, and discussion. (DOC) [file pone.0033268.s013.doc]

**ADDITIONAL MATERIALS AND METHODS AND RESULTS AND DISCUSSION**

Contents

[MATERIALS AND METHODS 2](#__RefHeading___Toc316991838)

[RNA extraction 2](#__RefHeading___Toc316991839)

[Milk yield and Milk Composition Analysis 2](#__RefHeading___Toc316991840)

[Microarray protocol 2](#__RefHeading___Toc316991841)

[Production of labeled cDNA 2](#__RefHeading___Toc316991842)

[Microarray hybridization and image acquisition 3](#__RefHeading___Toc316991843)

[Quantitative PCR for microarray verification 4](#__RefHeading___Toc316991844)

[Protocol used for REVIGO 5](#__RefHeading___Toc316991845)

[RESULTS AND DISCUSSION 5](#__RefHeading___Toc316991846)

[Impact of DEG on Gene Ontology terms 5](#__RefHeading___Toc316991847)

[Most impacted Gene Ontology Biological process terms 5](#__RefHeading___Toc316991848)

[Most impacted Gene Ontology Cellular components terms 7](#__RefHeading___Toc316991849)

[Additional notes on Gene Ontology analyzed by DIA 8](#__RefHeading___Toc316991850)

[Additional databases analysis 8](#__RefHeading___Toc316991851)

# MATERIALS AND METHODS

## RNA extraction

Biopsy tissue was weighed (~0.5-1.0 g) and immediately subjected to RNA extraction using ice-cold Trizol (Invitrogen Corp., San Diego, CA) as previously described . RNA integrity was assessed by electrophoretic analysis of 28S and 18S rRNA subunits; all samples were deemed of good/excellent integrity. Genomic DNA was removed from RNA with DNAse (Qiagen, Valencia, CA) using RNeasy Mini Kit columns (Qiagen). RNA concentration was measured using a NanoDrop ND-1000 spectrophotometer (www.nanodrop.com). The purity of RNA (A_260_/A_280_) was above 1.9 for all samples.

## Milk yield and Milk Composition Analysis

Milk yield was electronically recorded twice daily (each milking) during the entire lactating period, i.e., over 300 individual measurements for each cow. Milk composition analyses, including protein % and yield, lactose % and yield, fat % and yield, and also a large fatty acid profile of milk fat throughout lactation have been previously published .

## Microarray protocol

### Production of labeled cDNA

We used an annotated bovine oligonucleotide microarray containing >10,000 unique elements . Hybridizations were performed in 120 microarrays in a dye-swap reference design. The reference was made by pooling RNA from several bovine tissues. The cDNA was obtained by reverse transcriptase in a 30 μL reaction adding 10 μg RNA, 2 µL of random hexamer primers (3 μg/μL; Invitrogen Corp., CA), 1 µg oligo dT18 (Operon Biotechnologies, Huntsville, AL), and DNase-RNase-free water to a volume of 17.78 μL. The mixture was incubated at 65°C for 5 min and kept on ice for 3 min. To the mixture were added 12.2 μL solution composed of 6 μL 5X First-Strand Buffer, 3 μL 0.1 M DTT, 0.6 μL 100 mM dNTP mix (Invitrogen Corp.), 0.12 μL of 50 mM 5-(3-aminoallyl)-dUTP (Ambion, CA), 2 μL (100 U) of SuperScriptTM III RT (Invitrogen Corp.), and 0.5 μL of RNase Inhibitor (Promega, Agora, WI). The reaction was performed at 23°C for 1 min and 46°C for 9 h. The cDNA obtained was then treated with 10 µL 1 M NaOH, and incubated for 15 min at 65°C to remove residual RNA. The solution was neutralized by adding 10 µL 1 M HCl. The unincorporated 5-(3-aminoallyl)-dUTP and free amines were removed using a Qiagen PCR Purification Kit (Qiagen). Clean cDNA was vacuum-dried and resuspended in 4.5 µL 0.1 M Na2CO3 buffer (pH 9.0) and 4.5 µL of Amersham CyDye™ fluorescent dyes diluted in 60 µL of DMSO (Cy3 or Cy5; GE Healthcare, Waukesha, WI, USA). Binding of Cy dyes with 5-(3-aminoallyl)-dUTP incorporated into cDNA was obtained by incubation at room temperature for 1 h. The unbound dyes were removed using a Qiagen PCR Purification Kit (Qiagen) and clean labeled cDNA was measured by means of a NanoDrop ND-1000 spectrophotometer. An average of 5.6 ± 1.6 μg of cDNA and 7.7 ± 2.6 μM of Cy dyes were obtained for each reaction. Sample and reference were then vacuum-dried in the dark.

### Microarray hybridization and image acquisition

Prior to hybridization, slides were re-hydrated, placed in an UV cross-linker, washed with 0.2% SDS solution, thoroughly rinsed with purified water to remove un-bound oligonucleotide, and pre-hybridized using a solution containing 1% albumin, 5 × SCC, and 0.1% SDS at 42 C° for ≥45 min with the aim of decreasing background. After pre-hybridization, slides were rinsed with abundant purified water and immersed in isopropanol for ~10s and spin-dried. Dried slides were immediately hybridized in a dye-swap-reference design (i.e., each sample was labeled twice with each of the two dyes and hybridized in each slide with the reference labeled with the opposite dye). Labeled cDNA of the sample was re-hydrated with 80 µL of hybridization buffer #1 (Ambion, Austin, TX) and mixed thoroughly. This solution was used to re-suspend the reference sample labeled with the opposite dye and mixed thoroughly in order to obtain a homogenous solution of the two labeled cDNA. Before hybridization, the labeled cDNA resuspension of the sample + reference was incubated at 90-95ºC for ca. 3 min to denature cDNA in order to increase the efficiency of oligo’s binding onto the slide.

Hybridizations were carried out using humidified slide chambers (Corning, Lowell, MA) with cover slips (LifterSlip; Thermo Scientific, Billerica, MA) at 42°C for ca. 40 hours in the dark. After hybridization, slides were removed from the chamber and washed for 5 min by agitation 3 times with wash buffers in the following order: 1×SSC and 0.2% SDS solution preheated at 42°C, 0.1×SSC and 0.2% SDS solution, and 0.1×SSC solution. Lastly, slides were inserted into a 50 mL tube, spin-dried and gassed with Argon to preserve dye from bleaching. Arrays were scanned with a ScanArray 4000 (GSI-Lumonics, Billerica, MA) dual-laser confocal scanner and images were processed and edited using GenePix 6.0 (Axon Instruments). Array quality was assessed using an in-house parser written in Perl language as previously described . Spots that received a -100 flag by GenePix 6.0 were removed from further analysis and background intensity was subtracted from the foreground intensity. Spots on the slide were considered “good” if the median intensity was ≥3×standard deviation above median background for each channel (i.e., dye). Spots were flagged “present” when both dyes passed the criteria, “marginal” if only one dye passed the criteria, or “absent” when both dyes failed to pass the criteria. Statistical analysis was conducted on oligos that were flagged as “present” and “marginal”. The microarray data presented in this manuscript have been deposited at NCBI's Gene Expression Omnibus and are accessible through GEO Series accession number GSE19055.

## Quantitative PCR for microarray verification

The protocols for cDNA synthesis, real time RT-PCR (qPCR), primer design and testing have been previously described in detail . The normalization of PCR data was obtained using the geometric mean of *UXT*, *RPS9* and *RPS15* as previously reported . More than 80% of the 90 genes tested whose protein products are mainly involved in lipid, lactose, and protein synthesis have been verified (Additional file S5).

## Protocol used for REVIGO

We uploaded the lists of GO ID with the average of impact or direction of the impact for the time point comparisons during full lactation (i.e., from 15 to 120d) compared to -30d. The list of GO with a positive direction (i.e., overall activated) and the list of GO with a negative direction (i.e., overall inhibited) as calculated by the DIA were analyzed separately. The analysis was run by selecting the medium size of the resulting list, with the numbers associated to GO categories “higher is better” (with the exception of the list of direction with an overall inhibition where the “lower is better” was chosen), with the default database GO category sizes (i.e., UniProt), and the SimRel as the semantic similarity measure . The software provides several visualizations of the results and one table with 300 less dispensable terms. The Scatterplot and the TreeMap were chosen to visualize the results.

# RESULTS AND DISCUSSION

## Impact of DEG on Gene Ontology terms

The KEGG pathways provided a suitable means to interpret the biological phenomena by relying on established metabolic and signaling pathways, but only 35.5% of the genes in our microarray platform were present in the KEGG dataset. A more holistic interpretation of our data could be provided by using Gene Ontology (GO). The GO is made of three categories: Biological process (BP), Molecular function, and Cellular components (CC). For this manuscript we will only discuss in detail the BP and CC. The BP and CC terms were associated with >55% of the annotated genes in our microarray.

### Most impacted Gene Ontology Biological process terms

The DIA analysis clearly highlighted as the most impacted and induced terms during lactation ‘Lactose biosynthesis’, followed by ‘Synthesis of phosphatidylcholine’, ‘Negative regulation of inflammatory response to antigenic stimulus’, ‘Regulation of mammary gland epithelial cell proliferation’, ‘Response to hydroperoxide’, ‘Angiogenesis involved in wound healing’, ‘Positive regulation of hypersensitivity and inflammatory response’, ‘Golgi organization’, and ‘Negative regulation of lipid catabolism’ (Figure 3 in main body and Additional file S1, sheet ‘GO Biological process’). Other terms induced and highly impacted were related to synthesis of glycerols such as ‘DAG biosynthesis’, ‘Regulation of STAT protein import into nucleus’, and GO terms related to transport of several components included amino acids (in particular aspartate and glutamate) and phosphate (Figure 3 in main body and Additional file S1, sheet ‘GO Biological process’). The most impacted and inhibited terms were positive regulation of leukocyte chemotaxis and monocyte activation (Additional file S1, sheet ‘GO Biological process’). The summary by REVIGO (Figure 3 in main body) allow to further emphasized the great impact of lactation on Golgi organization, regulation of inflammatory response, regulation of mammary proliferation and angiogenesis, and the negative regulation of lipid catabolism. Most of those processes can be easily ascribed to the mammary gland function during lactation and confirm previous results from KEGG analysis (see main body of the paper).

From a metabolic point of view the analysis indicated an increase of DAG and TAG biosynthesis, increase in TCA cycle, use of acetyl-CoA, and respiration (Figure 3 in main body). The latter also is probably associated with the large impact and induction in ‘Response to hydroperoxide’ (Figure 3 in main body). Those data supported the findings of metabolic increase in KEGG pathways discussed above. Few findings, however, appear to be in contradiction with the conclusion from the KEGG pathway analysis. One of those was the above mentioned activation of TGF-beta signaling and increase in peroxisomes. The former appears in GO BP as being inhibited by increased activity of proteins involved in the ‘Negative regulation of TGF-beta signaling pathway’ (Figure 3). For the latter only the import of the proteins into the peroxisomal membrane appeared to be inhibited, which is in accord with the overall visualization of the KEGG peroxisome pathway (Additional file S2). The significance of those findings is not readily apparent.

REVIGO clustered together several GO BP terms with an apparent greater inhibition during lactation (Figure 4). Those terms were related to immune response, with evident inhibition of immune cells (e.g., ‘Positive regulation of leukocytes chemotaxis’ and ‘Monocytes activation’) and MHC-I activity (i.e., ‘Antigen processing and presentation’), tissue remodeling (including neurons, e.g., ‘Regulation of epidermis differentiation’, ‘Regulation of glial cell differentiation’, ‘Regulation of cell fat differentiation’), tissue sensory capacity including sensing bacteria (e.g., ‘Response to mechanical stimulus’, ‘Detection of bacteria’), synthesis of several components including phospholipids and cysteine (e.g., ‘Phosphatidylserine biosynthesis’, ‘Cysteine biosynthesis’), catabolism of DAG and lipoproteins (e.g., ‘DAG catabolism’, ‘Lipoprotein catabolism’), transports of several components such as chloride, arginine, and ammonia, modification and metabolism of several RNA types (e.g., ‘tRNA modification’, ‘snRNA metabolism’), epigenetic modifications (e.g., ‘Regulation of histone methylation’, ‘Protein demethylation and dealkylation’), cell cycle (e.g., ‘Regulation of cytokinesis’, ‘Establishment of mitotic spindle location’), ‘Negative regulation of cell adhesion’, and ‘Lysosomal lumen acidification’ (e.g., see details in Additional file S1, sheet ‘GO Biological process’).

### Most impacted Gene Ontology Cellular components terms

The results for the GO Cellular component (GO CC) analysis run with DIA (Additional file S1, sheet ‘GO Cellular component' and Figures S6 and S7) indicated that the most impacted and induced cellular components were related with the ‘GPI-anchor transamidase complex’, supporting the findings with the KEGG analysis (see main body of the paper for discussion). Several other components were clustered by REVIGO with the GPI-anchor transamidase complex such as ‘Rough endoplasmic reticulum’, ‘Golgi stack’, vesicles (e.g., ‘Acrosomal vesicle’, ‘Recycling endosome’), and other membrane-bound organelles (e.g., ‘Peroxisomal part’ and ‘Lysosomal lumen’), the ‘Telomeric region of chromosomes’, the ‘PgC protein complex’ (involved in epigenetic repression of gene expression ), the ‘Basal lamina’ and the ‘Apical plasma membrane’ of the cell, and several cell junctions (e.g., ‘Focal adhesion’).

Among the most impacted and inhibited GO Cellular components during lactation, REVIGO uncovered as unique terms euchromatin components (e.g., ‘Euchromatin’, ‘Chromocenter’), particularly related to epigenetic modification (e.g., ‘Histone deacetylase complex’), the intramembrane components of peroxisome and mitochondria, the sarcoplasmic reticulum (SR) lumen, the trans-Golgi network (TGN), the MHC-I, components of the protein synthesis machinery (e.g., ‘Small ribosomal subunit’ and ‘Translation elongation factor 1 complex’), and cell division components (e.g., ‘Cell division site part’).

Most of the above findings support the results from the GO BP and the KEGG analyses. Among the relatively new findings by this analysis were the importance of the epigenetic regulation and the inhibition of the SR and the TGN during lactation. Overall, the results indicated an inhibition of the active components of the epigenetic modification and increase of long term epigenetic stabilizer (e.g., PcG protein complex, ). The TGN is involved in the later stages of protein secretion, but it appears in our data that it was inhibited during lactation. This finding is supported by a previous study where the TGN was observed to be absent in lactating mammary . The SR is important for intracellular accumulation and homeostasis of calcium. It is still not fully clear how the large quantity of calcium is transported through the mammary cell without disrupting the intracellular signaling . The role of SR in Ca homeostasis in lactating mammary remains unclear . The overall inhibition of the SR in our data indicated that this compartment is not essential for Ca transport during lactation in bovine.

### Additional notes on Gene Ontology analyzed by DIA

We have observed that most of the terms highly impacted and induced during full lactation (i.e., from 15 to 120 vs. -30d) were inhibited at 120 vs. 240d (Additional file S1), when the lactation evidently declined (Figure S1). This appears to be an indication that maintenance of lactation in bovine is highly-dependent on transcriptome adaptations of genes involved in the functions highlighted by the DIA analysis.

## Additional databases analysis

Many databases are available to be mined by DAVID . Similarly, with the DIA we have analyzed additional databases. Those include the GO Molecular function (GO MF), Swiss-Prot (SP) and Protein Information Resource (PIR) keywords (SP_PIR_Keywords), the integrative protein signature database (InterPro), the Clusters of Orthologous Groups (COG) ontology, the Protein Information Resource (PIR) superfamily, the Simple Modular Architecture Research Tool (SMART, that allows the identification and annotation of genetically mobile domains and the analysis of domain architectures), the protein Superfamily (SSF), and the UniProt sequence feature (UP_Seq_Feature). The complete results from the DIA analysis is reported in Additional file S1 and for the GO MF also Figures S8 and S9. From those additional datasets few unique observations can be made. The DIA results of almost all the additional databases uncovered as the most impacted terms during lactation those known to be related to milk synthesis (e.g., ‘Lactose synthesis’ in GO MF and SP_PIR_Keywords, ‘Milk fat globule protein among’ PIR superfamily, ‘Lactoalbumin’ and ‘Caseins’ in Interpro) and confirmed results from the GO BP and KEGG pathways discussed above.

Additional results from the DIA analysis using the supplementary databases were the large impact and induction in GO MF during lactation (Additional file S1, GO Molecular function) including: ‘Stearoyl-CoA 9-desaturase activity’ and terms related to transport of several components such as phosphate and amino acids (confirmed previous data ( and references therein) but also revealed ‘Copper transport’, probably associated with the increase in energy metabolism and angiogenesis in mammary during lactation (see main body of the paper and above); ‘Phosphatidylserine binding’ which is probably related to the large degree of up-regulation of milk fat globule-EGF factor 8 protein (see companion paper) to specifically bind such components of the inner leaflet of the plasma membrane, particularly during involution but in our case it appeared to be important during lactation; ‘Clathrin binding’, associated with the intracellular transfer of membrane by coated vesicles and in mammary appears to be involved in secretory pathways ; ‘Proteasome regulator activity’, indicating a decrease of inner cell protein degradation as suggested also by DIA analysis of KEGG pathways (Additional file S1, ‘KEGG pathway’ sheet) and observed previously in bovine and in mouse mammary during lactation; ‘Oxidoreductase activity’ mostly due to the large increase in expression of several oxidoreductase enzymes such as *XDH*, gluthatione peroxidase 1, or lactoperoxidase (see companion paper) but also activation of oxidative phosphorylation and energy metabolism in general (Additional file S1, ‘KEGG pathway’ sheet). Among the most inhibited GO MF there was ‘Phosphoenolpyruvate carboxykinase activity’ indicating a decrease of gluconeogenesis in bovine mammary (Additional file S1, ‘GO Molecular function’ sheet). It was reported earlier that bovine mammary is unable to perform gluconeogenesis . However, recent data in rat mammary revealed a decrease in expression of Pck1 during lactation (in our data *PCK2* expression was decreased, see companion paper) and also there is evidence for gluconeogenic capacity in mouse epithelial cells treated with prolactin . Together those recent data suggest that mammary epithelium can perform gluconeogenesis, thus, our data might indicate an overall reduction of gluconeogenesis to further increase the availability of glucose for lactose synthesis.

References

1. Loor JJ, Everts RE, Bionaz M, Dann HM, Morin DE, et al. (2007) Nutrition-induced ketosis alters metabolic and signaling gene networks in liver of periparturient dairy cows. Physiol Genomics 32: 105-116.

2. Bionaz M, Loor JJ (2008) Gene networks driving bovine milk fat synthesis during the lactation cycle. BMC Genomics 9: 366.

3. Bionaz M, Loor JJ (2011) Gene networks driving bovine mammary protein synthesis during the lactation cycle. Bioinform Biol Insights 5: 83-98.

4. Edgar R, Domrachev M, Lash AE (2002) Gene Expression Omnibus: NCBI gene expression and hybridization array data repository. Nucleic Acids Res 30: 207-210.

5. Bionaz M, Loor JJ (2007) Identification of reference genes for quantitative real-time PCR in the bovine mammary gland during the lactation cycle. Physiol Genomics 29: 312-319.

6. Supek F, Bosnjak M, Skunca N, Smuc T (2011) REVIGO Summarizes and Visualizes Long Lists of Gene Ontology Terms. PLoS One 6: e21800.

7. Bauman DE, Mather IH, Wall RJ, Lock AL (2006) Major advances associated with the biosynthesis of milk. J Dairy Sci 89: 1235-1243.

8. Davis CL, Bauman DE (1983) Mammary Gland Metabolism. In: Mepham TB, editor. Biochemistry of Lactation. Amsterdam: Elsevier Science Publishers B.V. pp. 3-30.

9. Schwartz YB, Pirrotta V (2008) Polycomb complexes and epigenetic states. Curr Opin Cell Biol 20: 266-273.

10. Clermont Y, Rambourg A, Hermo L (1995) Trans-Golgi network (TGN) of different cell types: three-dimensional structural characteristics and variability. Anat Rec 242: 289-301.

11. Shennan DB (2008) Calcium transport by mammary secretory cells: mechanisms underlying transepithelial movement. Cell Mol Biol Lett 13: 514-525.

12. Huang da W, Sherman BT, Lempicki RA (2009) Systematic and integrative analysis of large gene lists using DAVID bioinformatics resources. Nat Protoc 4: 44-57.

13. Tisato F, Marzano C, Porchia M, Pellei M, Santini C (2010) Copper in diseases and treatments, and copper-based anticancer strategies. Med Res Rev 30: 708-749.

14. Nakatani H, Aoki N, Nakagawa Y, Jin-No S, Aoyama K, et al. (2006) Weaning-induced expression of a milk-fat globule protein, MFG-E8, in mouse mammary glands, as demonstrated by the analyses of its mRNA, protein and phosphatidylserine-binding activity. Biochemical Journal 395: 21-30.

15. Pearse BM (1976) Clathrin: a unique protein associated with intracellular transfer of membrane by coated vesicles. Proc Natl Acad Sci U S A 73: 1255-1259.

16. Pauloin A, Tooze SA, Michelutti I, Delpal S, Ollivier-Bousquet M (1999) The majority of clathrin coated vesicles from lactating rabbit mammary gland arises from the secretory pathway. J Cell Sci 112 ( Pt 22): 4089-4100.

17. Finucane KA, McFadden TB, Bond JP, Kennelly JJ, Zhao FQ (2008) Onset of lactation in the bovine mammary gland: gene expression profiling indicates a strong inhibition of gene expression in cell proliferation. Funct Integr Genomics 8: 251-264.

18. Lemay DG, Neville MC, Rudolph MC, Pollard KS, German JB (2007) Gene regulatory networks in lactation: identification of global principles using bioinformatics. BMC Syst Biol 1: 56.

19. Scott RA, Beuman DE, Clark JH (1976) Cellular gluconeogenesis by lactating bovine mammary tissue. J Dairy Sci 59: 50-56.

20. Hsieh CW, Huang C, Bederman I, Yang J, Beidelschies M, et al. (2011) Function of phosphoenolpyruvate carboxykinase in mammary gland epithelial cells. J Lipid Res 52: 1352-1362.
